# Using Seroprevalence and Immunisation Coverage Data to Estimate the Global Burden of Congenital Rubella Syndrome, 1996-2010: A Systematic Review

**DOI:** 10.1371/journal.pone.0149160

**Published:** 2016-03-10

**Authors:** Emilia Vynnycky, Elisabeth J. Adams, Felicity T. Cutts, Susan E. Reef, Ann Marie Navar, Emily Simons, Lay-Myint Yoshida, David W. J. Brown, Charlotte Jackson, Peter M. Strebel, Alya J. Dabbagh

**Affiliations:** 1 Public Health England, 61 Colindale Ave, London NW9 5EQ, United Kingdom; 2 Aquarius Population Health, London, United Kingdom; 3 University of Bristol, University of Bristol, School of Social and Community Medicine, Canynge Hall, 39 Whatley Road, Bristol BS8 2PS, United Kingdom; 4 Duke University Medical Center, Durham, NC, United States of America; 5 Johns Hopkins Bloomberg School of Public Health, Baltimore, MD, United States of America; 6 London School of Hygiene & Tropical Medicine, Keppel Street, London WC1E 7HT, United Kingdom; 7 World Health Organization, 20 Ave Appia, 1211 Geneva 27, Switzerland; 8 Centers for Disease Control and Prevention, 1600 Clifton RD., NE, Atlanta, GA 30333, United States of America; 9 Institute of Tropical Medicine, Nagasaki University, Nagasaki, Japan; 10 University College London, London, United Kingdom; 11 Influenza and Measles Laboratory, IOC, Fiocruz, Rio de Janeiro, Brazil; University of Cambridge, UNITED KINGDOM

## Abstract

**Background:**

The burden of Congenital Rubella Syndrome (CRS) is typically underestimated in routine surveillance. Updated estimates are needed following the recent WHO position paper on rubella and recent GAVI initiatives, funding rubella vaccination in eligible countries. Previous estimates considered the year 1996 and only 78 (developing) countries.

**Methods:**

We reviewed the literature to identify rubella seroprevalence studies conducted before countries introduced rubella-containing vaccination (RCV). These data and the estimated vaccination coverage in the routine schedule and mass campaigns were incorporated in mathematical models to estimate the CRS incidence in 1996 and 2000–2010 for each country, region and globally.

**Results:**

The estimated CRS decreased in the three regions (Americas, Europe and Eastern Mediterranean) which had introduced widespread RCV by 2010, reaching <2 per 100,000 live births (the Americas and Europe) and 25 (95% CI 4–61) per 100,000 live births (the Eastern Mediterranean). The estimated incidence in 2010 ranged from 90 (95% CI: 46–195) in the Western Pacific, excluding China, to 116 (95% CI: 56–235) and 121 (95% CI: 31–238) per 100,000 live births in Africa and SE Asia respectively. Highest numbers of cases were predicted in Africa (39,000, 95% CI: 18,000–80,000) and SE Asia (49,000, 95% CI: 11,000–97,000). In 2010, 105,000 (95% CI: 54,000–158,000) CRS cases were estimated globally, compared to 119,000 (95% CI: 72,000–169,000) in 1996.

**Conclusions:**

Whilst falling dramatically in the Americas, Europe and the Eastern Mediterranean after vaccination, the estimated CRS incidence remains high elsewhere. Well-conducted seroprevalence studies can help to improve the reliability of these estimates and monitor the impact of rubella vaccination.

## Introduction

Congenital Rubella Syndrome (CRS) is a preventable cause of infant mortality and lifelong disability. Previous analyses concluded that approximately 110,000 (range: 14,000–308,000) children were born with CRS in 1996 in 78 (developing) countries which had not introduced rubella-containing vaccine (RCV) in their national programme[1]. Updated estimates are important, given growing activity in controlling and eliminating rubella and CRS. By 2010, 130 countries had introduced RCV nationally, compared with 83 reporting use by 1996[2] and three of the six WHO regions had established rubella control/elimination and CRS prevention/elimination goals[3]. The new Global Vaccine Action Plan has goals of establishing regional elimination of measles and rubella in at least five WHO regions by 2020[4].

In some countries, rubella and CRS outbreaks continue to occur regularly. For example, in Vietnam, over 400 newborns suspected to have CRS were identified during January 2011 –December 2012, at least 6 months following the peak in the preceding rubella epidemic[5]. Such outbreaks are consistent with the relatively high (30%) percentage of women who were found to be seronegative in a recent seroprevalence in Vietnam[6], and are preventable with sufficiently high levels of vaccination coverage. RCVs are given with measles vaccines and, given the high coverage of measles-containing vaccine (MCV) in many countries[7], WHO recommends that countries use opportunities offered by accelerated measles control and elimination activities to introduce RCVs[8]. In November 2011, the Global Alliance on Vaccines and Immunization (GAVI) released funding for eligible countries which were not currently using RCV in their routine programs to conduct a measles-rubella (MR) vaccine catch-up campaign and subsequently introduce RCV into their routine programs[9]. Countries applying for funding are requested to include data on disease burden and epidemiology of rubella.

CRS incidence is typically underestimated in routine surveillance, so that its magnitude is best estimated using seroprevalence data. Using datasets identified through a literature search in mathematical models, we update previous work to consider all countries and estimate the CRS incidence until 2010.

## Methods

### Data sources

#### Literature search

We conducted a systematic review of the literature using the criteria and databases in Table 1 to identify studies published between 1990 and December 2011 with age-specific seroprevalence data for rubella.

**Table 1:** Literature search strategy and inclusion criteria for datasets.

**Language**	English, French, Spanish, Portuguese, German, Italian or Korean.
**Search terms**	[“rubella” OR “rubeol*”] AND [“seroepidemiolog*” OR “seroprevalen*” OR “prevalen*” OR “seroimmun*” OR “rubella antibod*”].
**Databases**	Pubmed, Web of Science, Embase, CAB Abstracts, Global Health, African Healthline, BDENF, Scopus, CINAHL, EMRO (IMEMR), LILACS, MedCarib, WPRIM (WPRO), PAHO, WHOLIS, and AFRO.
**Criteria for extracting data identified in the literature review (“eligible” datasets)**	1. Population considered had no major biases (i.e. did not consider individuals with rash, health care workers, etc). 2. Included data for at least two age groups aged over 15 years. 3. There was no evidence that individuals included in the dataset had received rubella vaccination, for example, in the private sector. 4. The age-specific proportion susceptible decreased irregularly with increasing age.
**Criteria for using extracted data to estimate CRS burden**	1. At least one of the catalytic models used (see main text) gave a plausible fit to the data. 2. The best-fitting force of infection was non-zero. 3. The upper limit on the 95% CI was not 100%.

The search was conducted in two stages: the first was conducted in August 2008, considering the period 1990-August 2008; a subsequent search was conducted in December 2011 to identify studies that had been published since then. We identified additional datasets from citations in published papers, unpublished literature reviews (*WHO; AM Navar et al*), previous reviews[1] (which included data published before 1990) and unpublished datasets from representative populations in South India[10] and Kilifi (Kenya)[11]. The latter datasets are provided in Tables A and B in S1 File. An additional dataset from Central Vietnam was identified which had not been published at the time that the review was carried out, but has since been published[6]. One of the unpublished reviews was provided by co-authors (PMS and AJD) from the World Health Organization, who had commissioned that review to be carried out; the other unpublished review was provided by the reviewer (AMN), who is also a co-author on this paper. The unpublished data were provided by the investigators of the corresponding studies and co-authors of this paper (FTC and DWJB). Collection of the data from South India had IRB approval from the London School of Hygiene & Tropical Medicine (LSHTM), the Indian Council of Medical Research and the Christian Medical College in Vellore, South India. Collection of the data from Kenya had ethics approval from the Kenyan Medical Research Institute/National Ethical Review Committee and LSHTM. Both studies obtained informed consent from participants.

After reviewing the abstracts, potentially relevant articles were read in full and age-specific numbers of seropositive and seronegative individuals were extracted from eligible datasets, by gender where possible (see Table 1 for eligibility criteria and criteria for using extracted datasets). None of the selected datasets overlapped in their study populations. Unless otherwise indicated, individuals of “child-bearing” age were assumed to be aged 15–44 years. Where equivocal results were presented, they were interpreted as being seropositive (Text A in S1 File provides details of rubella immunity testing). In practice, most studies stated their definition of an equivocal titre without reporting the number of samples that were equivocal. For those datasets, we used the given study’s interpretation of equivocal.

#### Demographic data

The total population size, age-specific numbers of females for 1996 and 2000–2010 and age-specific fertility rates, were extracted for each country from UN population databases[12]. Fertility rates were available only for 5-year periods (1995–2000, 2000–2005 and 2005–2010), and annual values were assumed to equal the average for the corresponding 5-year period. Country-specific annual numbers of births, by maternal age group for 1996 and 2000–2010 were calculated from corresponding fertility rates and female population size.

#### Vaccination data

By 2010, all countries in Europe and the Americas, and 3/47, 15/22, 4/11 and 20/26 countries in the African, Eastern Mediterranean, SE Asian and Western Pacific Regions respectively had introduced RCV. Using the estimated annual number of live births in these countries and the total number of live births in each region, we then calculated the percentage of all the live births in each region which were occurring in countries which had introduced RCV by given years.

Countries have submitted annual vaccination coverage data to WHO since 1980[13]. Since 2000, WHO and the United Nations Children’s Fund (UNICEF) jointly review these and available special survey data to obtain the WHO-UNICEF coverage estimates (“WUENIC”)[13]. For countries reporting having national RCV policies, but lacking RCV coverage data, we assumed that RCV coverage equalled the WUENIC estimate[14] for the first and second doses of MCV (MCV1 and MCV2) and the reported coverage if this was unavailable. If neither was available, that for the most recent or earliest subsequent year was used instead.

Historical data on the target population and the estimated coverage for periodic mass RCV campaigns (“supplementary immunization activities”, or “SIAs”) available from WHO[15] and elsewhere (Text C in S1 File) were also used. For countries known to vaccinate adolescent girls (“selective vaccination”), we used published coverage data where possible (Text C in S1 File); otherwise, we assumed 50% coverage until the first cohort eligible for a second dose of RCV reached adolescence, when we used RCV2 coverage data. The effects of 10% and 90% selective coverage were also explored. Private sector and post-partum vaccination were not included, given the lack of reliable coverage data. Missing SIA or routine coverage data were supplemented from publications (Text C in S1 File).

### Estimating the CRS incidence per 100,000 live births

#### Overview

We use catalytic models[16] to analyse the seroprevalence data to estimate the average age-specific force of infection (rate at which susceptibles are infected) in different settings. These estimates were then used to define the force of infection (and therefore the age-specific proportion susceptible for each country) before the introduction of RCV. For countries which had not introduced RCV by 2010, the force of infection estimates were used in equations to calculate the incidence of CRS directly. For countries which had introduced RCV by 2010, the force of infection estimates were used to calculate age-dependent contact parameters, which were then included in an age-structured dynamic transmission Susceptible-Preinfectious-Infectious-Recovered (SEIR) model, which included vaccination. The transmission models were then used to calculate the incidence of CRS.

#### Analyses of the seroprevalence data

Four catalytic models, denoted A, B, C and D (Table 2) were fitted to each eligible serological dataset collected before RCV was introduced to estimate the average annual “force of infection” among <13 and ≥13 year olds (i.e. the rate at which susceptible <13 and ≥13 year olds are infected), denoted *λ*_*y*_ and *λ*_*o*_ respectively. The subscripts “y” and “o” refer to “younger” and “older” people. This age-stratification approximates changes in school attendance and therefore exposure to infection in many countries.

**Table 2:** Summary of the catalytic models used in the analyses of serological data. Note that the lower case letter “*a*” in the equations below refers to the single year band, whereas “*A*” (see Eq 1 in the main text) refers to those in the age group of interest, *A*.

Model	Assumption
A	The force of infection differs between younger and older individuals and was estimated, and the sensitivity of the assay was unknown and was estimated, together with the force of infection. The proportion of individuals of age *a* (*s*_*n*_*(a)*) that are seronegative are given by the equations: *s*_*n*_*(a)* = 1*-p(1-e*^*-λ*^_*y*_^*(a-0*.*5*^*)* for *a*<13 years and *s*_*n*_*(a)* = 1*-p(1-e*^*-λ*^_*y*_^*12*.*5*^*e*^*-λ*^_*o*_^*(a-13)*^*) a*≥13 years, where *p* is the sensitivity of the serological assay, and *λ*_*y*_ and *λ*_*o*_ are the average force of infection among younger and older individuals^+^ respectively. The equation for the proportion susceptible in a given age group is given in Text A in S1 File.
B^$^	The force of infection differs between younger and older individuals and was estimated, and the sensitivity of the assay was fixed at 100%. This model is similar to that used previously[1].
C^$^	The force of infection was identical for younger and older individuals, but the sensitivity of the assay could be <100% and was identical for all ages. Both the force of infection and the sensitivity of the assay were estimated. This model is equivalent to the variable asymptote model defined by Muench[16].
D^$^	The force of infection was identical for all age groups and was estimated; the sensitivity of the assay was fixed at 100%. This model is equivalent to the simple catalytic model[16].

^+^ We refer to younger and older individuals as those aged <13 and ≥13 years respectively.

%

^$^ The equation for the age-specific proportion susceptible for models B-D can be obtained by substituting for *p* = 1.00 and/or *λ*_*y*_ and *λ*_*o*_, as appropriate into the equation for Model A.

Each model incorporated maternally-derived immunity in infants, and in two models the sensitivity of the assay was estimated with the force of infection (Table 2). The models were fitted using maximum likelihood using an algorithm based on the simplex method of Nelder and Mead, written in the C programming language[17] (Text A in S1 File). 95% confidence intervals (CI) for the force of infection and (where applicable) the sensitivity of the assay for each dataset and model were calculated using non-parametric bootstrap for binary data, based on 1000 bootstrap datasets, following Shkedy et al[18] (Text A in S1 File). The force of infection estimates from one of the four catalytic models for each dataset were selected to define the age-specific proportion susceptible before the introduction of vaccination and then to calculate the national, regional and global CRS incidence (see below). The force of infection estimates were selected in decreasing order of biological plausibility of the model (referred to here as selection criterion 1), coming from model A, unless they met specific criteria (the force of infection was implausibly high (>600 per 1000 per year), zero in either age group, higher for older individuals than for children or its upper confidence limit was 100%). If this occurred, we used estimates from model B in preference to those from model C, and those from model C in preference to those from model D. If no model fitted the data convincingly, occurring when the best-fitting age-specific proportion seronegative passed through the 95% confidence limits of just one of the observed datapoints, the dataset was dropped from further analyses.

We assessed the sensitivity of selection criterion 1 by comparing the incidence of CRS predicted using the selected model against that calculated using an alternative selection criterion (referred to here as “selection criterion 2”). This criterion was based on the Akaike’s Information Criterion (AIC) score, corrected for small samples (AICc)[19], whereby the model with the lowest AICc score was selected, unless its estimates met the same exclusion criteria that were described above for criterion 1. If this occurred, the model with the next lowest AICc score without any of the exclusion criteria was selected. For large samples, the value for the AICc converges to that obtained using the AIC. Therefore, using the AICc for all datasets, irrespective of their sample size, will lead to the same conclusions as those obtained using the AICc and AIC for datasets with small and large samples respectively.

In sensitivity analyses, we explored the effect of excluding datasets by recalculating the burden of CRS (see below) after including any datasets that had been dropped because of their poor fit to the data. In further sensitivity analyses, we recalculated the burden of CRS (see below) after excluding each individual dataset one at a time from the calculations, in order to explore the contribution of each individual dataset to the estimates. To reduce computational burden, this analysis was restricted just to the four World Health Organization regions which made up the greatest contribution (>99%) to the global burden of CRS in 2010.

### Country-specific CRS incidence per 100,000 live births

#### Countries which had not introduced RCV by 2010

For countries for which only one serological dataset was available, the best-fitting force of infection was used to calculate the CRS incidence per 100,000 live births (*I*_*CRS*_*(A)*) by maternal age group A (15–19, 20–24, 25–29, 30–34, 35–39, 40–44 and 45–49 years) using Eq 1[1]:

$$I_{CRS}(A)=s(A)(1-e^{-16\lambda_{o}/52})\times 0.65\times 100000$$ Eq 1

where *s(A)* is the proportion of women of age group *A* that were susceptible. From previous work[1, 20–21], we assumed that 65% of babies born to mothers infected during the first 16 weeks of pregnancy had CRS. The CRS incidence per 100,000 live births among 15–44 year olds for a given year, *t*, was calculated as the corresponding average for 5-year age groups in this range, weighted by the number of live births in each 5-year maternal age group in that year using the following equation:

$$I_{CRS}^{B}(A_{15-44},t)=\frac{\sum_{i=1}^{6}I_{CRS}(A_{i})B_{o,i}(t)}{\sum_{i=1}^{6}B_{o,i}(t)}$$ Eq 2

where *A*_*i*_ refers to age group *i*, where *i* = 1, 2, …6 corresponds to the age groups 15–19, 20–24, 25–29, 30–34, 35–39 and 40–44 years respectively, and *B*_*o*,*i*_*(t)* is the observed number of livebirths occurring among women in age group *i* in year *t*. The 95% CI was approximated as the 95% range of the values obtained by repeating this calculation for each of the 1000 force of infection estimates obtained for the bootstrap datasets associated with the serological dataset for the country.

For countries for which multiple serological datasets were available, the calculation was similar, except that the force of infection estimates that were used came from equal numbers of randomly-sampled bootstrap datasets associated with each serological dataset, or, if the serological datasets were clearly described as being urban or rural, in proportion to the population living in urban and rural areas. For countries without serological datasets, the force of infection estimates came from equal numbers of bootstrap datasets from each country in the same WHO region as the country of interest. Tables C, D and E in S1 File include further details of the datasets used. The bootstrap estimates for the Western Pacific excluded estimates for China and Australia, since these countries were atypical of the region. In sensitivity analyses, we explored the effect of using 1000 force of infection estimates which came from equal numbers of the bootstrap datasets associated with each country in the corresponding Global Burden of Disease (GBD) region[22] (Tables D and E in S1 File).

#### Countries which had introduced RCV by 2010

We used a dynamic, age and sex-structured transmission model[23] (i.e. one that incorporated contact and transmission between different age groups), to calculate the median and 95% range of the CRS incidence per 100,000 live births for countries which had introduced RCV by 2010. Text C in S1 File includes further details and the equations in the model.

The model was first used to recreate the epidemiology of rubella in each country before vaccination had been introduced, using 1000 values of age-dependent contact parameters, which differed between countries. These contact parameters were calculated using established methods (Text C in S1 File) from 1000 bootstrap-derived estimates of the force of infection from before the introduction of vaccination for each country. The force of infection estimates were compiled using the same approach as that used for countries which had not introduced vaccination by 2010 (see above, Tables C, D and E in S1 File), namely by fitting catalytic models to seroprevalence data from the country concerned, if such data were available, or compiled from force of infection estimates from countries in the same region, if such data were not available. When calculating the contact parameters using force of infection estimates, we based our assumptions about contact between children and adults on data from the POLYMOD study[24]. Data on the age-specific coverage of RCV every year since its introduction were incorporated into the model, and the model was run for each of the 1000 sets of contact parameters to calculate the CRS incidence per 100,000 live births. We took the median of the resulting values and 95% CI were approximated by their 95% range. In sensitivity analyses, we explored the effect of basing the contact parameters on force of infection estimates which came from datasets from countries in the same GBD region as the country of interest[22] (Tables D and E in S1 File).

#### Regional estimates

The regional median and 95% CI of the CRS incidence per 100,000 live births was calculated from bootstrap-derived estimates for each country described above, weighted by the population size. The equation for the *j*^*th*^ bootstrap estimate of the average CRS incidence per 100,000 live births for the *N* countries in a given region was calculated using the following equation:

$$\frac{\sum_{c=1}^{N}I_{CRS,c,j}^{B}(A_{15-44},t)P_{c}(t)}{\sum_{c=1}^{N}P_{c}(t)}$$

where $I_{CRS,c,j}^{B}(A_{15-44},t)$ is the *j*^*th*^ bootstrap estimate of the CRS incidence per 100,000 live births among those aged 15–44 years for country *c* in year *t* (see Eq 2 above) and *P*_*c*_*(t)* is the population size of country *c* in year *t*.

#### Estimating the number of CRS cases born annually

The number and 95% range of CRS cases born annually in a given country, region and globally were also calculated by using the bootstrap-derived estimates. Specifically, we first multiplied each bootstrap-derived estimate of the CRS incidence per live birth among mothers in each 5-year age group in 1996 and annually between 2000 and 2010 by the total number of births among mothers of the corresponding age group. This calculation provided the total number of CRS cases by 5-year maternal age group in each country for the given bootstrap estimate. We then summed the annual numbers for the j^th^ bootstrap for each country in the region to obtain the corresponding regional totals, which were summed to obtain the global burden. These calculations were repeated for each of the 1000 bootstrap estimates. The 95% CI of the national, regional and global numbers of CRS cases were approximated by the 95% range of the corresponding 1000 values. In sensitivity analyses, the totals for 1996 for the countries included in previous analyses[1] were also calculated.

## Results

### Literature search and analyses of seroprevalence data

**Fig 1:**
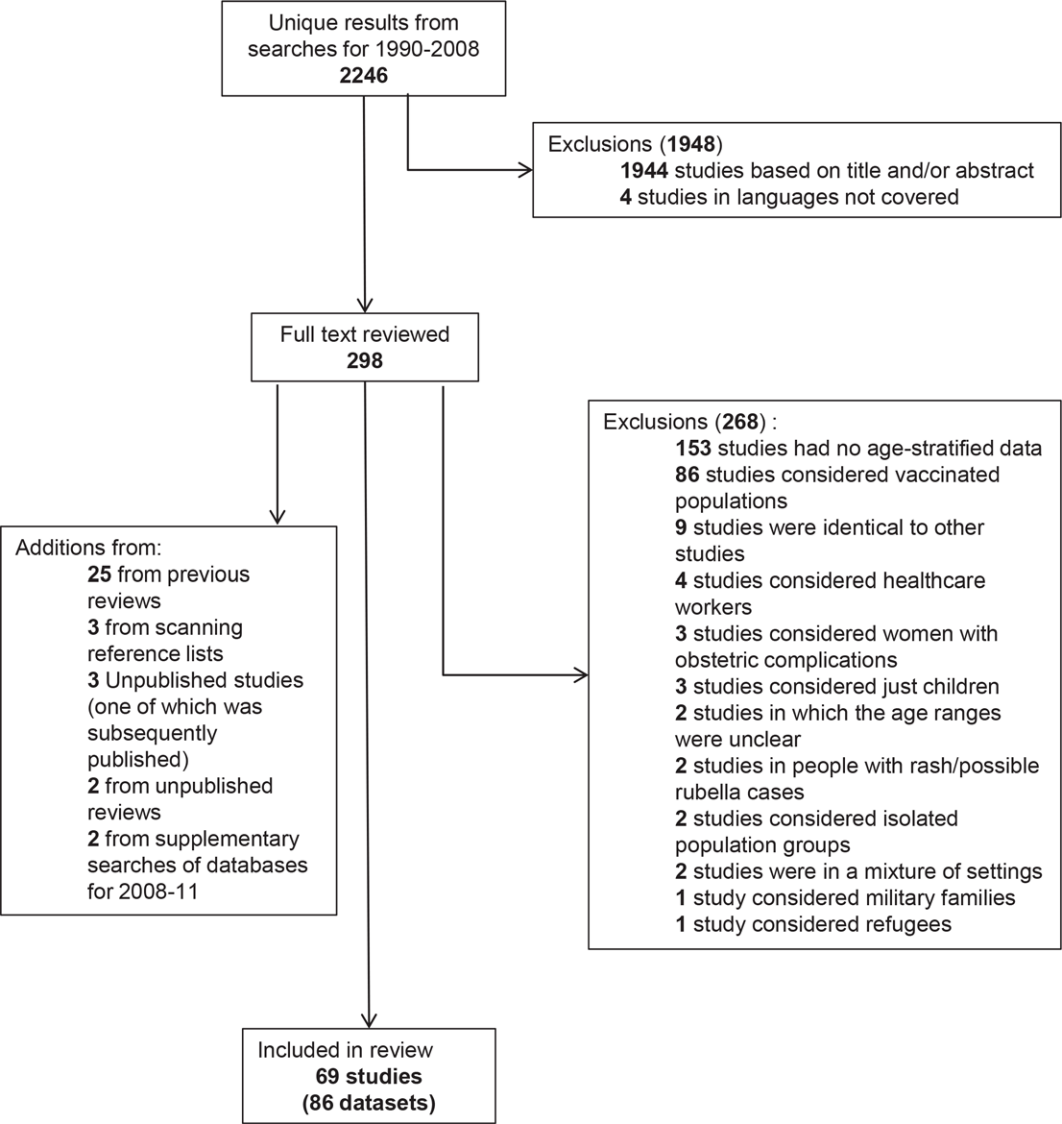
Results of the literature search for age-specific serological data collected before the introduction of rubella vaccination.

Fig 1 summarizes the results from the literature search. After de-duplication and excluding ineligible studies, 69 studies, comprising 86 usable serological datasets collected before RCV was introduced, from 69 papers were identified for calculating CRS incidence. Thirty two datasets came from 22 countries which had not introduced RCV by 2010 and 54 datasets came from 36 countries which had done so by 2010 (Table 3). In total, 17, 22, 13, 13, 11 and 10 datasets were available for the African, American, Eastern Mediterranean, European, South East Asian and Western Pacific regions respectively (Table 3). These were of variable size and quality. The Eastern Mediterranean had the largest percentage of datasets (54% or 7/13) which had a sample size exceeding 1000 individuals, as compared with 41% (7/17) for the African region, and decreasing to 18% (4/22) for the Americas. However, for each region, the datasets came from fewer than half of the constituent countries, ranging from 41% (9/22) in the Eastern Mediterranean and 36% (4/11) in South East Asia to 25–30% in the four other regions.

**Table 3:** Summary of the serological datasets collected before the introduction of vaccination which were used to estimate the global burden of CRS.

	Africa	Americas	E Mediterranean	Europe	SE Asia	W Pacific
**No. countries represented**	13	12	9	13	4	8
**No. of countries in region**	47	39	22	51	11	27
**Datasets from countries where RCV had not been introduced by 2010**	Benin (1993); Congo (<1991); Cote d’Ivoire (1975, 1985–6); Ethiopia (1981, 1994); Gabon (1985); Ghana (1997); Kenya (1996–7); Madagascar (1990–5); Mozambique (2002); Nigeria (<1978, <2002, 2007–8); Senegal (1996–2001); South Africa (2003); Zambia (1979–80)	-	Pakistan (<1997, 1999–2004); Yemen (1985, 2002–3)		Bangladesh (2004–5); India (1968, 1968, 1972–3, 1972–3, 1976, <1987, <1990, 1999–2000); Nepal (2008)	Vietnam (2009–10)
# datasets (# with SS^+^ >1000)	17 (7)	-	4 (2)	-	10 (3)	1 (1)
**Datasets from countries where RCV had been introduced by 2010**	-	Argentina (1967–8,1967–8); Brazil (1967–8,1987); Canada (<1967); Chile (1967–8,1967–8); Haiti (2002); Jamaica (1967–8,1967–8); Mexico (1987–8,1989); Panama (1967–8,1967–8); Peru (1967–8,2003); Trinidad (1966–7,1967–8); Uruguay (1967–8,1967–8); USA (<1967,<1967)	Bahrain (1981); Iran (1993–5); Jordan (1982–3); Kuwait (<1978); Lebanon (1980–1); Morocco (1969–70); Saudi Arabia (1989, 1992–3); Tunisia (<1970)	Czech Republic (<1967); Denmark (<1967,1983); Germany (1990); England (<1967,1986–7); Finland (1979); France (<1967); Kyrgyzstan (2001); Romania (<1989); Turkey (1998,2003–4)	Thailand (1978)	Australia (<1967?); China (1979–80); Fiji (<1973); Japan (<1967, <1967); Malaysia (<1972); Singapore (1975–9); Taiwan (1984,1984–6)
# datasets (# with SS^+^ >1000)	-	22 (4)	9 (5)	13 (4)	1 (0)	9 (4)

* The year in which the study was carried out is not known for several studies. For these studies, the table includes “<” followed by the year of publication.

^+^ SS = sample size.

**Fig 2:**
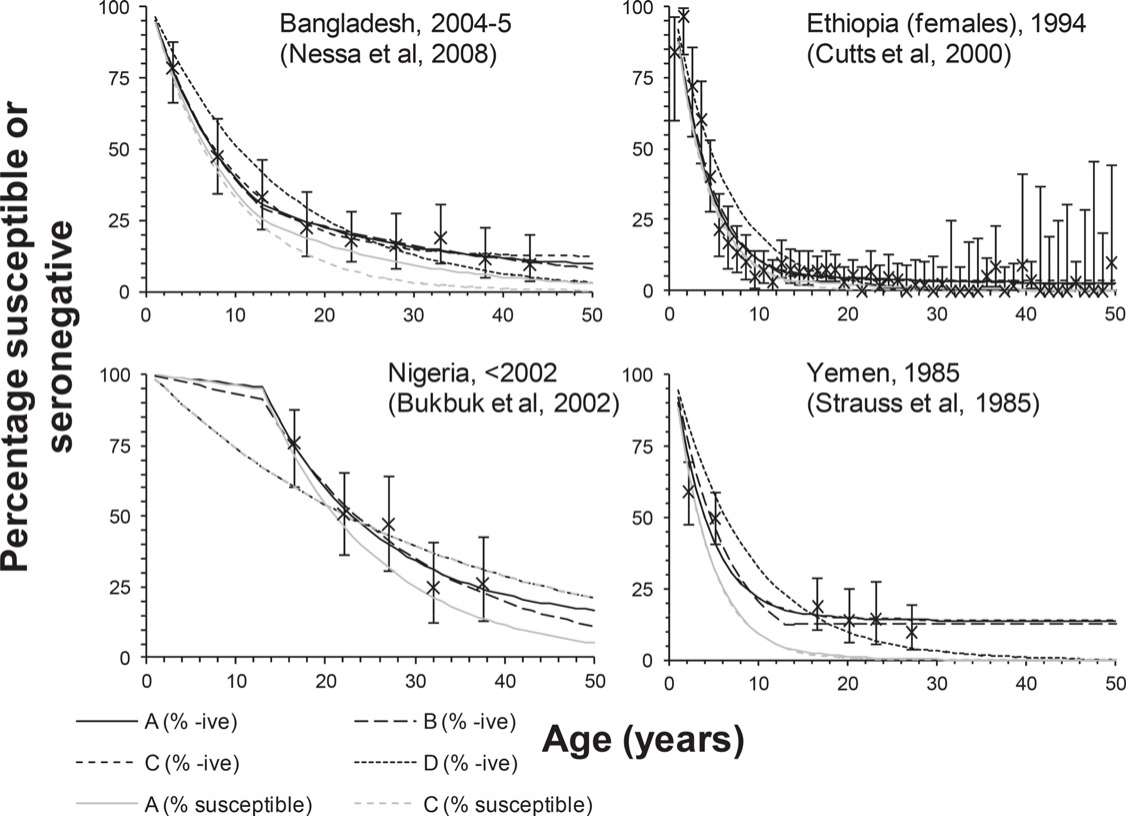
Examples of the fit of catalytic models to the data sets. Comparison between model predictions of the percentage susceptible and the percentage seronegative to rubella obtained using the four types of catalytic model (denoted by the lines labelled A, B, C and D), and that observed in various settings. The crosses show the observed percentage seronegative together with 95% (exact) confidence intervals.

Fig 2 and Table 4 show examples of the datasets identified and the fits of each catalytic model to the data; Table H and Table I and Figs B-E in S1 File provide details of the fits for all the datasets. After applying the selection criteria for the catalytic models, two datasets–one from Niger[25] and the other from Cote d’Ivoire[26]—were dropped from the main analysis due to the poor fit of the selected catalytic model to the data (Table H and Table I and Fig E in S1 File). For many datasets, the catalytic model selected as being the most appropriate, based on biological plausibility, was identical to that based on the AICc criterion (Table H and Table I in S1 File). For the datasets for which this did not occur, the 95% CI for the CRS incidence per 100,000 live births for the two selected models typically overlapped.

**Table 4:** Examples of the results obtained by fitting the catalytic models to data. The best-fitting values for the force of infection and (where appropriate) the sensitivity of the antibody assay, and the CRS incidence per 100,000 live births for each catalytic model The values in parentheses reflect the 95% confidence intervals, obtained by bootstrapping. To facilitate comparisons, the infection and CRS incidence is not weighted by the number of live births.

Country, year of study*	Catalytic model	Force of infection (/1000/year) for <13 yr olds	Force of infection (/1000/year) ≥13 yr olds	Sensitivity (%)	CRS/ 100,000 live births	Log-likelihood deviance (degrees of freedom)	AICc	Selected model by Criterion 1 (biological plausibility)	Selected model by AICc
Bangladesh, 2004–05[40]	A	110 (84,143)	59 (22,1000)	93 (82,100)	126 (18,168)	1.7 (6)	50	B	B
	B	99 (82,120)	35 (16,54)	—	118 (59,169)	1.8 (7)	45		
	C	117 (88,157)	117 (88,157)	88 (83,94)	121 (69,174)	2.0 (7)	45		
	D	70 (62,79)	70 (62,79)	—	215 (194,232)	16.6 (8)	56		
Ethiopia (Addis Ababa), 1994[41]	A	261 (230,295)	83 (23,164)	98 (96,100)	20 (12,28)	75.1 (47)	194	A	A
	B	233 (215,252)	26 (9,47)	—	19 (7,30)	78.4 (48)	195		
	C	269 (237,305)	269 (237,305)	96 (96,97)	13 (8,21)	79.3 (48)	196		
	D	169 (158,183)	169 (158,183)	—	57 (47,67)	206.9 (49)	321		
Nigeria, <2002 [42]	A	4 (0,23)	78 (41,168)	88 (68,100)	496 (334,526)	1.7 (2)	52	C	D
	B	7 (0,26)	57 (37,74)	—	449 (305,518)	1.9 (3)	32		
	C	32 (26,38)	32 (26,38)	100 (100,100)	260 (250,264)	8.0 (3)	38		
	D	32 (26,38)	32 (26,38)	—	260 (250,264)	8.0 (4)	31		
Yemen, 1985[43]	A	255 (179,364)	169 (104,1000)	85 (80,91)	96 (89,99)	13.3 (3)	57	C	C
	B	163 (139,185)	0 (0,40)	—	87 (82,90)	20.4 (4)	54		
	C	258 (183,365)	258 (183,365)	85 (80,90)	96 (90,99)	13.3 (4)	47		
	D	116 (100,134)	116 (100,134)	—	76 (71,81)	45.4 (5)	74		

*The year in which the study was carried out is not known for several studies. For these studies, the table includes “<” followed by the year of publication.

### Estimated CRS incidence per 100,000 live births

#### Countries which had not introduced RCV by 2010

**Fig 3:**
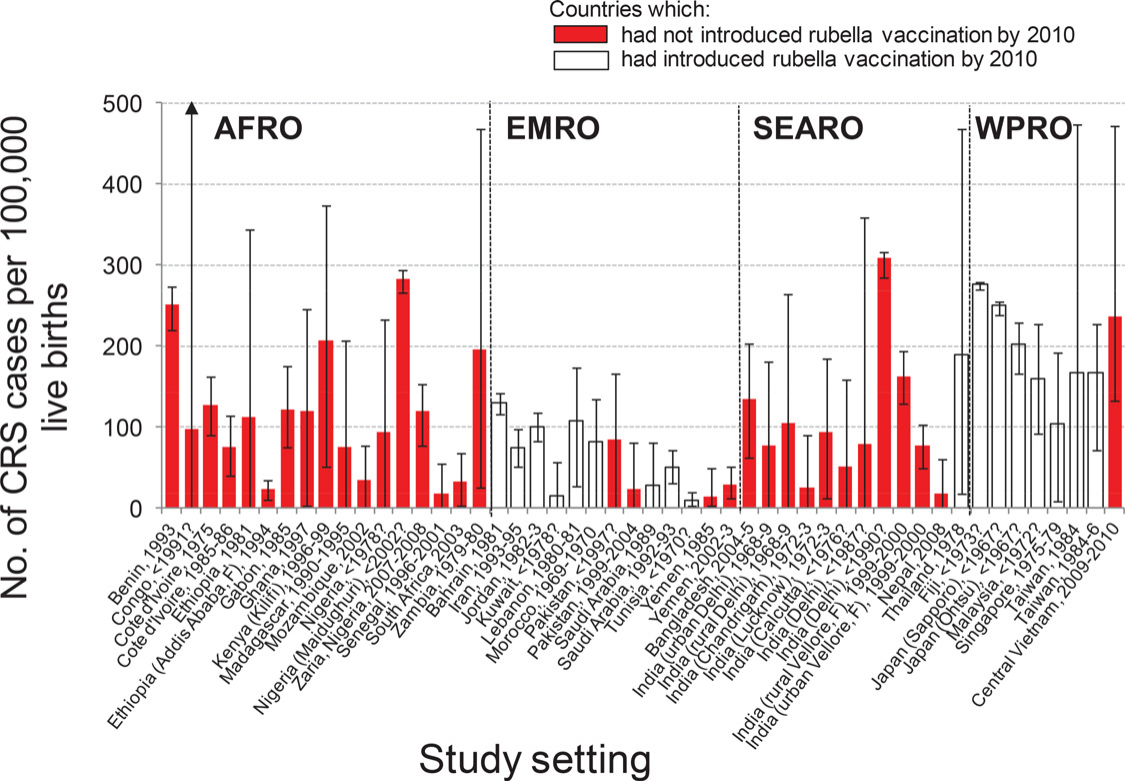
Estimates of the number of CRS cases per 100,000 live births among women aged 15–44 years obtained using datasets from countries in which RCV had not been introduced at the time of collection. The red bars reflect countries which had not introduced RCV by 2010; the white bars indicate countries which had introduced RCV by 2010. The estimates have been weighted by the number of live births in the corresponding country in 2010. Labels on the x-axis denote the year of data collection; uncertain dates of collection are indicated using a question mark. The countries are grouped by WHO regions (AFRO = African, EMRO = Eastern Mediterranean, SEARO = South East Asian, WPRO = Western Pacific).

The estimated CRS incidence varied greatly between and within regions and countries (Fig 3). In Africa, it ranged from 19 (95% CI: 0–56) per 100,000 live births in Senegal to 283 (95% CI: 266–293) per 100,000 live births in Maiduguri, Nigeria and from 24 (95% CI: 10–36) to 112 (95% CI: 0–344) per 100,000 live births in urban Addis Ababa and rural Ethiopia respectively (Fig 3). There were nearly five-fold variations in the Eastern Mediterranean, while in SE Asia, estimates ranged from 18 (95% CI: 0–60) per 100,000 live births in Nepal (2008) to 309 (95% CI: 285–317) per 100,000 live births in Delhi (1980s). Estimates from the Western Pacific ranged between 105 (95% CI: 10–193) and 276 (95% CI: 271–280) per 100,000 live births, with the most recent estimate (Central Vietnam, 2009–10) being 237 (95% CI: 133–472) per 100,000 live births.

#### Countries which had introduced RCV by 2010

**Fig 4:**
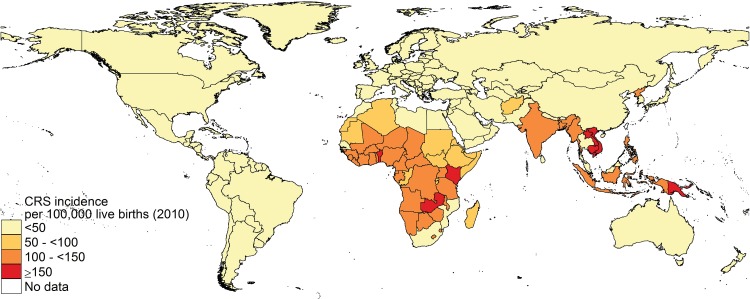
Estimates of the median incidence of CRS per 100,000 live births by country in 2010.

For most countries in the Americas, the estimated average CRS incidence was close to zero from 2000–10, with wide CI for several (Fig 4 and Fig F in S1 File). The estimated CRS incidence was also low by 2010 for countries in the Eastern Mediterranean, SE Asia and the Western Pacific which had introduced RCV by 2010 (Fig 4). Estimates for European countries were low after they introduced RCV (Fig F in S1 File), and were mostly close to zero by 2010.

#### Regional estimates

The estimated incidence was lowest in the Americas, Eastern Mediterranean and Europe, reaching <0.01 (95%CI: 0–1), 25 (95% CI: 4–61) and 1 (95%CI: 0–5) per 100,000 live births by 2010 respectively (Table 5). By 2010, 100% (Americas and Europe) and 42% (Eastern Mediterranean) of the regional live births occurred in countries which had introduced RCV.

**Table 5:** The median CRS incidence per 100,000 live births and number of CRS cases born in each WHO region and worldwide in 1996, 2000 and 2010 and the percentage of the regional birth cohort living in countries which had introduced RCV by these years.

	Year	% of the regional live births occurring in countries which had introduced RCV	CRS incidence per 100,000 live births	Total number of CRS cases
Africa	1996	0.09	115 (55,231)	28315 (13443,57421)
	2000	0.08	116 (55,232)	30464 (14411,61846)
	2010	0.09	116 (56,235)	38712 (18063,79852)
Americas	1996	61	56 (24,104)	10640 (4394,19867)
	2000	91	11 (6,23)	2514 (1160,4990)
	2010	100	<0.01 (0,1)	<1 (0,136)
Eastern Mediterranean	1996	21	56 (22,106)	7625 (2577,15290)
	2000	38	42 (16,82)	6216 (1927,12580)
	2010	42	25 (4,61)	5294 (827,12358)
Europe	1996	52	65 (14,133)	8155 (1839,15349)
	2000	69	45 (6,114)	6004 (1030,13266)
	2010	100	1 (0,5)	98 (1,507)
South East Asia	1996	3	130 (43,251)	50128 (14587,96435)
	2000	3	126 (39,246)	48252 (13196,93822)
	2010	3	121 (31,238)	49229 (11204,96976)
Western Pacific (excluding China)*	1996	11	118 (58,225)	11368 (5137,21938)
	2000	11	117 (60,206)	10922 (5020,20115)
	2010	92	90 (46,195)	8889 (4010,21118)
Western Pacific (including China)	1996	17	30 (15,55)	11541 (5268,21980)
	2000	24	30 (15,52)	11084 (5328,20167)
	2010	42	23 (12,50)	8889 (4010,21118)
Global	1996	**—**	**—**	119224 (72119,169107)
	2000	**-**	**-**	107156 (62121,154446)
	2010	**-**	**-**	105391 (53605,158041)

In Africa and SE Asia, where <0.1% and 3% respectively of the regional live births lived in countries which had introduced RCV by 2010, the average annual CRS incidence in 2010 was high: 116 (95% CI: 56–235) and 121 (95% CI: 31–238) per 100,000 live births respectively. For the Western Pacific including or excluding China, the CRS incidence in 2010 was 23 (95% CI: 12–50) and 90 (95% CI: 46–195) per 100,000 live births respectively.

#### Estimated numbers of CRS cases

Globally, the estimated average annual number of CRS cases (Table 5) decreased from 119,000 cases in 1996 (95% CI: 72,000–169,000) to 107,000 cases by 2000 (95% CI: 62,000–154,000), and approximately 105,000 cases by 2010 (95% CI: 54,000–158,000).

As expected, decreases were marked in regions where most countries had introduced RCV (Table 5 and Table J in S1 File). For the Americas, the average number of CRS cases decreased from 11,000 (95% CI: 4,400–20,000) to 2,500 (95% CI: 1,200–5,000) cases between 1996 and 2000, and <1 case (95% CI: 0–136) in 2010.

**Fig 5:**
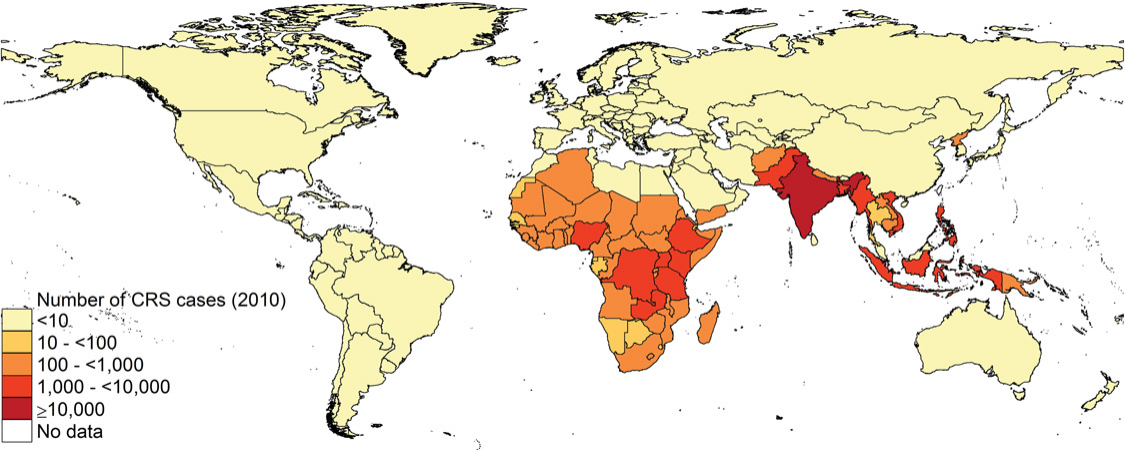
Estimates of the median numbers of CRS cases born by country in 2010.

In the Western Pacific, about 11,000 cases (95% CI for 1996: 5,000–22,000) were estimated annually between 1996 and 2009, and 9,000 cases (95% CI: 4,000–21,000) in 2010. For SE Asia, similar numbers of cases were predicted annually during 1996–2010, with about 49,000 cases (95% CI: range: 11,000–97,000) estimated in 2010 (Table 5), and India experiencing the highest incidence (40,000 (95% CI: 0–86,000) cases/year) (Fig 5).

For Africa, the estimated average CRS incidence increased by about 10,000 cases between 1996 and 2010, to reach about 39,000 cases (95% CI: 18,000–80,000), resulting largely from temporal increases in the number of births. More than 100 cases were estimated to have been born in 2010 in most countries in Africa (Fig 5), with seven countries (DR Congo, Ethiopia, Kenya, Nigeria, Tanzania, Zambia and Uganda) having >1000 cases/year.

#### Sensitivity analyses

Estimates of the burden of CRS in 2010 globally were relatively insensitive to the selective vaccination coverage of adolescents (Table K in S1 File), as were the estimates for all regions, except for Europe, where many countries had introduced selective coverage. For Europe in 2010, estimates obtained assuming high levels of selective coverage were about 80% lower than those based on low levels of coverage.

Including estimates obtained using the two datasets (from Niger[25] and Cote d’Ivoire[26]) for which the catalytic models fitted poorly led to slightly increased estimates of the number of CRS cases in Africa, compared to that in the base-case (Table L in S1 File), although the 95% CI overlapped (42,000 (95% CI: 21,000–81,000) compared to 39,000 (95% CI: 18,000–80,000) in the base-case). As a result, the global number of CRS cases was also slightly increased compared to the base case (Table L in S1 File), when the two datasets were included (108,000 (95%CI: 57,000–163,000) in 2010, compared to 105,000 (95% CI: 54,000–158,000) respectively).

Figs G-J in S1 File show the effects of excluding individual datasets from calculations of the country-specific CRS incidence. For countries which had no serological datasets from before the introduction of RCV (and whose serological profile was based on all datasets from the same region), the estimated CRS incidence changed little after excluding individual datasets (Figs G-J in S1 File). For many countries which had a dataset from before RCV was introduced, the estimated CRS incidence changed only slightly after excluding the dataset from the calculations, with the confidence intervals overlapping or remaining within those calculated when the dataset had been included. However, for some countries (e.g. Gabon, Ghana, Madagascar, Mozambique, Senegal, South Africa, Nepal, Tunisia, China and Malaysia), the confidence intervals widened when the dataset for their country was excluded from calculations.

The greatest uncertainty in the regional CRS incidence per 100,000 Iivebirths, weighted by the population size and the regional or global number of CRS cases in 2010 was associated with the dataset from China[27] (Figs K-M in S1 File). For example, excluding any single dataset, excepting that for China, scarcely affected these statistics. However, excluding the dataset from China led to a greatly increased upper 95% confidence limit for each statistic. Considering the global number of CRS cases, for example, the upper confidence limit increased from 158,000 in the basecase to 200,000 when the dataset was excluded (Fig M in S1 File).

The CRS incidence in 1996 in the countries considered previously[1], as calculated using the current approach (111,000, 95% CI: 64,000–161,000) was similar to previous estimates (110,000 cases (95% CI: 14,000–308,000). Text F in S1 File includes further details.

Regional estimates were insensitive to use of WHO or GBD groupings of countries when selecting datasets for countries without serological data (Table J in S1 File), except for SE Asia. Here, estimates using the GBD regional grouping greatly exceeded those obtained using the WHO regional grouping (56,000 vs. 49,000 cases, 95% CI: 14,000–100,000 and 11,000–97,000 respectively), leading to increased estimates of the global number of CRS cases in 2010, namely 113,000 (95% CI: 66,000–163,000) and 105,000 (95% CI: 54,000–158,000) respectively).

## Discussion

Our analyses suggest that the average CRS incidence per 100,000 live births decreased substantially between 1996 and 2010 in regions with high RCV coverage, but, given the large birth cohorts elsewhere, the estimated number of cases globally decreased modestly, from about 119,000 (95% CI: 72,000–169,000) cases in 1996 to about 105,000 (95% CI: 54,000–158,000) cases in 2010, with wide and overlapping confidence intervals.

High RCV coverage and campaigns in Latin American countries have reduced rubella incidence close to elimination. Most of the current estimated incidence is in Africa and SE Asia, where few countries have introduced vaccination. As many countries in these regions are GAVI-eligible, the situation may change quickly. Since spatial heterogeneity in vaccination coverage may lead to increases in CRS incidence[28], it is important to develop regional CRS control strategies and not to rely on the GAVI initiative alone, to avoid situations where one country introduces RCVs but its neighbour[29], failing to meet criteria for GAVI funding, does not prioritise rubella. For example, Nepal conducted a SIA in 2011[30] and is introducing RCV into its infant schedule. Successful CRS control in border areas may depend partly on India’s future rubella control activities. With many GAVI eligible and non-eligible countries sharing borders, this issue may become increasingly important in future[30].

As noted previously[1], the wide confidence intervals for our estimates reflect great variability in seroprevalence and quality of available data. Many datasets were convenience samples from antenatal clinics, which may not represent the general population. All of the datasets were from cross-sectional surveys, which means that, had an epidemic occurred just before the survey, the seroprevalence and therefore, the force of infection estimate would have been higher than that for a typical year, particularly for children. These factors relating to data quality would have contributed to the poor fit of the catalytic models to the observed data for some countries. Many countries lacked serological data, and data from elsewhere, according to GBD region or WHO region/geographical proximity, were used instead. Both methods led to similar estimates for all regions, except for SE Asia, where estimates based on GBD region were greatly increased. The latter estimates are unreliable, as for some GBD regions, serological datasets were available only from one country and, for some SE Asian groupings, only from settings with a high estimated CRS incidence (Table D and Table E in S1 File).

Our analyses rely on the assumption that the force of infection before the introduction of vaccination in a given country had remained unchanged over time. Secular changes in the force of infection could have occurred because of changes in population density, which would have led to an increased amount of contact between people, and therefore an increased opportunity for infection. It was not possible to test whether this might have occurred in reality, since none of the seroprevalence datasets came from the same population in different years. For example, whilst several seroprevalence datasets were available for some countries, they came from different parts of the country and therefore differences between the datasets could be due to regional differences in transmission rather than temporal differences.

In our analyses of the seroprevalence data, the overall fit of the selected catalytic model to the observed data was not good for some datasets, as reflected by the size of the loglikelihood deviance for the given number of degrees of freedom (Table 4 and Tables H-I in S1 File). The poor fit could be due to many of the reasons described above relating to data quality. However, we note that for the datasets for which the overall fit was not good, the selected model typically passed through the confidence intervals of over half of the observed seroprevalence datapoints. They also typically passed through those of the maternal age groups, which are most relevant for calculating the CRS incidence (see, for example, the plot for Yemen in Fig 2). Also, the confidence intervals associated with the estimated force of infection were usually wide, increasing the chance that the actual force of infection and CRS incidence in the population were within the range of those estimated.

Vaccination coverage data have several limitations[13]. Some countries introduced MCV2 or RCV2 before 2000, whereas corresponding coverage data were available only since 2000. Some countries lacked coverage data in some years and coverage was then assumed to equal that in adjacent years. Estimated trends in CRS incidence in regions with widespread RCV vaccination, however, mirror observed reductions in the reported CRS and rubella incidence[31–32].

Data on SIAs varied in quality, sometimes reporting unrealistically high (>100%) coverage. In such instances, we applied a level of coverage of 100% and the results would have been similar had the coverage been 99%. The model assumed that vaccination occurred on a single day, rather than over weeks or months, and could have slightly overestimated the rate at which SIAs affected rubella transmission. Estimates of the global burden were insensitive to the assumed selective vaccination coverage, since only a small proportion of the global birth cohort was eligible and for a limited period.

We did not assess the effect of heterogeneous vaccination coverage, which can potentially lead to increases in CRS incidence[23, 28, 33]. Likewise, we did not assess the stochastic effects of vaccination and local fade-outs. Modelling[34] has suggested that local extinction of rubella transmission could lead to increases both in the proportion of adults in isolated populations who are susceptible to infection, and CRS incidence. Vaccination programmes are most likely to miss such isolated populations. However, incorporating heterogeneous coverage would have scarcely affected our estimated global CRS incidence, given the small contribution to this estimate from countries which have introduced RCV. As further countries introduce RCV, however, it will be important to obtain good national and subnational coverage data, and to take action to ensure uniformly high coverage.

Our estimates did not account for the effect of rubella-related terminations of pregnancies, and therefore, we may have overestimated the CRS incidence per 100,000 live births. The size of the overestimate is unclear, due to the difficulty with measuring the proportion of all rubella infections during pregnancy that result in terminations of the pregnancy. It probably varies over time and between settings, depending on the availability of appropriate diagnostic and obstetric services. For example, studies have found that the proportion of all terminations in pregnancy that were rubella-related dropped with the reported rubella incidence, from 42% during the period 1970–74 to 20% and 0.5% during the periods 1975–9 and 1990–96 respectively[35].

In our analyses, equivocal serological results that were presented in datasets were interpreted as being seropositive. Since few studies presented equivocal results, the interpretation of equivocal results would not have greatly affected the estimated burden of CRS. Many factors may lead to equivocal results, including waning of antibody titres with time since infection. Since adults are more likely than young people to have been infected many years previously, such waning is consistent with increases in the proportion of antibody test results that are equivocal with increasing age, as seen in some studies[36]. In such instances, treating equivocal results as positive leads to a more reliable estimate of the force of infection in the past than that obtained by treating equivocal results as negative. Including equivocals as positive does not have a straightforward effect on the estimated CRS incidence. For example, it leads to a reduced estimated proportion of people that are susceptible, but the estimated force of infection is then slightly higher than it would be if equivocals are treated as seronegative. Since CRS incidence depends on both the force of infection and the proportion of people that are susceptible, these effects either balance themselves out or can lead to a slightly increased or decreased estimated CRS incidence.

Calculations of the CRS incidence for the Western Pacific excluded China, where the prevaccination incidence was considerably lower than elsewhere in the region, resulting from the high force of infection and high seropositivity (98%—see Table I in S1 File) among adolescents during 1979–80[27]. Even before RCV was introduced in 2008, the epidemiology of rubella in China may have changed since 1979–80, as suggested by high (25%) levels of susceptibility seen recently among female migrant workers in Shenzen in China[37], possibly resulting from demographic changes and vaccination in the private sector.

Our estimates suggest substantial under-reporting of CRS cases. For example, in 2000, the 54 countries submitting reports to WHO[38] reported only 181 CRS cases. In Europe, only 68 cases were reported during the period 2005–9[39], compared to about 6000 estimated for this period (Table J in S1 File). Several factors influence under-reporting, including lack of access or use of health services, poor or non-existent CRS surveillance systems and difficulty with detecting hearing impairment (a common defect associated with CRS) early in life.

CRS is associated with significant morbidity and remains a significant burden, particularly in Africa and SE Asia. Although the incidence is much lower than for major infectious diseases, including tuberculosis, malaria, and HIV in these regions, RCV is a simple intervention, and the opportunity cost of including rubella in measles control and elimination activities is relatively low. As momentum builds for measles elimination and RCV introduction, strong regional and sub-regional co-ordination and development of mechanisms is needed urgently to ensure that high coverage is reached and sustained in all communities[29]. Further surveillance and serological studies are needed both to improve the reliability of CRS incidence estimates and to monitor changes after vaccination is introduced.

## Supporting Information

S1 File Combined file contains the following: A: Analyses of the serological data collected before the introduction of RCV (text on: Unpublished datasets used in the analyses, Rubella immunity testing, Equations for the proportion susceptible, Fitting the seroprevalence data and calculating 95% CI; Tables A-B, which contain unpublished datasets). B: Sources of the bootstrap datasets (Tables C and D) and the bootstrap dataset used for each country (Table E). C: Description of the transmission model (text on: Model structure, Model equations, Contact parameters in the model, Vaccination coverage data; Tables F and G, which contain the model input parameters and variables, Fig A, which contains the model diagram). D: Results from fitting catalytic models to the serological data collected before the introduction of RCV (Figs B-E, Tables H-I). E: Estimates of the CRS incidence (Fig F and Table J). F: Sensitivity analyses (Table K on the effect of selective vaccination coverage; Table L on the effect of including additional datasets; Figs G-M on the effect of excluding individual datasets; text, Table SM and Fig N on “Comparison between the current and previous estimates obtained for 1996, for countries that had not introduced rubella-containing vaccine by 1996”.
(DOCX)

S2 File Prisma checklist. (DOC)

## Abbreviations

CRS: Congenital Rubella Syndrome
UNICEF: United Nations Children’s Fund
GBD: Global burden of disease
MCV: Measles-containing vaccine
RCV: Rubella-containing vaccine
SI: Supporting Information
