# Supplementary material for: Using Seroprevalence and Immunisation Coverage Data to Estimate the Global Burden of Congenital Rubella Syndrome, 1996-2010: A Systematic Review
Source: PLoS One. 2016 Mar 10;11(3):e0149160. doi: 10.1371/journal.pone.0149160 (PMC4786291; doi:10.1371/journal.pone.0149160)
Supplement: S1 File — Combined file contains the following: A: Analyses of the serological data collected before the introduction of RCV (text on: Unpublished datasets used in the analyses, Rubella immunity testing, Equations for the proportion susceptible, Fitting the seroprevalence data and calculating 95% CI; Tables A-B, which contain unpublished datasets). B: Sources of the bootstrap datasets (Tables C and D) and the bootstrap dataset used for each country (Table E). C: Description of the transmission model (text on: Model structure, Model equations, Contact parameters in the model, Vaccination coverage data; Tables F and G, which contain the model input parameters and variables, Fig A, which contains the model diagram). D: Results from fitting catalytic models to the serological data collected before the introduction of RCV (Figs B-E, Tables H-I). E: Estimates of the CRS incidence (Fig F and Table J). F: Sensitivity analyses (Table K on the effect of selective vaccination coverage; Table L on the effect of including additional datasets; Figs G-M on the effect of excluding individual datasets; text, Table SM and Fig N on “Comparison between the current and previous estimates obtained for 1996, for countries that had not introduced rubella-containing vaccine by 1996”. (DOCX) [file pone.0149160.s001.docx]

**Supporting Information**

**Using seroprevalence and immunisation coverage data to estimate the global burden of Congenital Rubella Syndrome, 1996-2010**

**Table of contents**

[A: Analyses of the serological data collected before the introduction of RCV 2](#_Toc443257680)

[1.1 Unpublished datasets used in the analyses 2](#_Toc443257681)

[1.2 Rubella immunity testing 3](#_Toc443257682)

[1.3 Equations for the proportion susceptible 3](#_Toc443257683)

[1.4 Fitting the seroprevalence data and calculating 95% CI 4](#_Toc443257684)

[1.4.1 The loglikelihood deviance 4](#_Toc443257685)

[1.4.2 Calculating 95% CI 4](#_Toc443257686)

[B: Sources of the bootstrap datasets 6](#_Toc443257687)

[C: Description of the transmission model 14](#_Toc443257688)

[3.1 Model structure 14](#_Toc443257689)

[3.2 Model equations 17](#_Toc443257691)

[3.3 Contact parameters in the model 17](#_Toc443257692)

[3.4 Vaccination coverage data 17](#_Toc443257693)

[D: Results from fitting catalytic models to the serological data collected before the introduction of RCV 17](#_Toc443257694)

[E: Estimates of the CRS incidence 17](#_Toc443257696)

[F: Sensitivity analyses 17](#_Toc443257697)

[6.1 The effect of selective vaccination coverage 17](#_Toc443257698)

[6.2 The effect of including additional datasets 17](#_Toc443257699)

[6.3 Effect of excluding individual datasets 17](#_Toc443257700)

[6.4 Comparison between the current and previous estimates obtained for 1996, for countries that had not introduced rubella-containing vaccine by 1996 17](#_Toc443257701)

# A: Analyses of the serological data collected before the introduction of RCV

## Unpublished datasets used in the analyses

Table : Numbers of females who were tested and positive for rubella antibodies in urban and rural areas in the study in Vellore, South India, 1999-2000 (Brown, Cutts, Samuel, Joseph, unpublished)[1].

|  | **Rural** | | **Urban** | |
| --- | --- | --- | --- | --- |
| **Age (years)** | **Number tested** | **Number positive** | **Number tested** | **Number positive** |
| 1 | 29 | 1 | 41 | 6 |
| 2 | 38 | 4 | 33 | 14 |
| 3 | 41 | 8 | 36 | 15 |
| 4 | 45 | 8 | 47 | 20 |
| 5 | 30 | 6 | 40 | 16 |
| 6 | 44 | 8 | 42 | 24 |
| 7 | 40 | 19 | 35 | 25 |
| 8 | 52 | 36 | 41 | 30 |
| 9 | 49 | 26 | 34 | 25 |
| 10 | 52 | 38 | 44 | 40 |
| 11 | 56 | 53 | 36 | 27 |
| 12 | 48 | 39 | 45 | 42 |
| 13 | 48 | 37 | 46 | 37 |
| 14 | 50 | 38 | 38 | 37 |
| 15 | 38 | 30 | 23 | 20 |
| 16 | 53 | 45 | 46 | 45 |
| 17 | 53 | 46 | 40 | 38 |
| 18 | 64 | 57 | 50 | 45 |
| 19 | 50 | 45 | 36 | 34 |
| 20 | 50 | 48 | 38 | 33 |
| 21 | 42 | 37 | 31 | 28 |
| 22 | 46 | 38 | 46 | 44 |
| 23 | 38 | 36 | 30 | 29 |
| 24 | 38 | 34 | 34 | 33 |
| 25 | 68 | 59 | 48 | 46 |
| 26 | 39 | 37 | 43 | 42 |
| 27 | 49 | 47 | 25 | 23 |
| 28 | 40 | 36 | 42 | 42 |
| 29 | 33 | 30 | 30 | 30 |
| 30 | 62 | 58 | 55 | 53 |
| 31 | 29 | 26 | 16 | 16 |
| 32 | 34 | 31 | 26 | 26 |
| 33 | 28 | 25 | 25 | 25 |
| 34 | 35 | 32 | 12 | 12 |
| 35 | 63 | 60 | 38 | 36 |
| 36 | 23 | 23 | 26 | 25 |
| 37 | 26 | 23 | 21 | 18 |
| 38 | 40 | 38 | 49 | 46 |
| 39 | 30 | 29 | 21 | 21 |

Table : Numbers of females who were tested and positive for rubella antibodies in the study in Kilifi, Kenya 1996-9 (Shulman et al, unpublished)[2-3]

| **Age group (years)** | **Number tested** | **Number positive** |
| --- | --- | --- |
| 14-19 | 57 | 37 |
| 20-24 | 106 | 78 |
| 25-29 | 67 | 53 |
| 30-34 | 28 | 24 |
| 35-43 | 18 | 15 |

## Rubella immunity testing

Rubella antibody screening for immunity has been widely used for 40 years. The test used has been refined over time. Initially screening was based on Haemagglutination Inhibition tests (HAI) and single Radical haemolysis (SRH) using a cut-off of 15 IU/ml, established to balance sensitivity of these tests against specificity (presence of low titre non-specific inhibition). Testing technology gradually changed to Enzyme-linked immunosorbent assay (EIA) through the 1980’s – 90’s initially using a cut-off of 10IU/ml and more recently to any detectible antibody level (4iu/ml). In parallel to these developments there has been a reduction in population antibody levels following widespread introduction of vaccine, which has been compensated for by improved sensitivity of assay.

## Equations for the proportion susceptible

For people in the age range *a_j_-a_k_*, denoted by the short-hand notation *A_j,k_*, the proportion susceptible in the catalytic model was given by the following equation:

The numerator in the first and last equation equals the difference in the proportion susceptible between the lower and upper ages in the age range of interest, and therefore the proportion newly infected in this age range and the denominator equals the force of infection (the rate at which they are infected) at this age, multiplied by the difference time spent in this age range. The equations can be derived by integrating the following expressions for the age-specific proportion susceptible over the age range of interest:

## Fitting the seroprevalence data and calculating 95% CI

### The loglikelihood deviance

The catalytic models were fitted using maximum likelihood by minimizing the following expression for the (binomial) loglikelihood deviance for each datasets comprising *D* datapoints:

where

*K_j_* is the number of individuals in the *j^th^* age group in the dataset who were seropositive;

*N_j_* is the number of individuals represented in the *j^th^* age group who were tested;

*p_j_* is the proportion of individuals in the *j^th^* age group in the dataset who were seropositive;

$\hat{p}_{j}$ is the model prediction of the proportion of individuals in the *j^th^* age group in the dataset who were seropositive, and equal to 1-proportion of people in the same age group who were seronegative (see SI 1.3 for the equations).

The age groups used were the ones provided in the corresponding publication describing the study.

### Calculating 95% CI

95% confidence intervals (CI) for the force of infection and (where applicable) the sensitivity of the assay for each dataset and model were calculated using non-parametric bootstrap for binary data, based on 1000 bootstrap datasets, following Shkedy et al[4].

With this approach, a single bootstrap dataset *B* (*B*=1..1000) comprised $K_{j}^{B}$ seropositive people in age group *j* among *N_j_* people in this age group who were tested. $K_{j}^{B}$ was obtained by first assigning the status “seropositive” or “seronegative” to each of the *N_i_* people in age group *j* in the dataset and drawing *N_j_* samples with replacement from this population. $K_{j}^{B}$ then equalled the number of people in the *N_j_* samples who were positive. The fitting was repeated for each of the bootstrap datasets and the 95% CI for the force of infection was calculated as the 95% range of the force of infection estimates obtained from the *B*=1..1000 bootstrap datasets.

# B: Sources of the bootstrap datasets

Table : Datasets used to set up bootstrap files for the WHO Regions. Note that these datasets had been accepted after performing the selection procedure described in the methods.

| **Region** | **Datasets** |
| --- | --- |
| African (AFRO) | Benin, 1993[5]; Congo, <1991[6]; Cote d'Ivoire, 1975[7] & 1985-6[8]; Ethiopia, 1981[9] & 1994[10]; Gabon, 1985[11]; Ghana, 1997[12]; Kenya, 1996-9 (Kilifi)[2-3]; Madagascar, 1990-1995[13]; Mozambique, 2002[14]; Nigeria, <1978[15], <2002[16] & 2007-8[17]; Senegal, 1996-2001[18]; South Africa, 2003[19], Zambia, 1979-80[20], |
| American, excluding Caribbean (AMRO, excl Caribbean) | Argentina, 1967-8 (urban & rural)[21], & 1981 (Mar de Plata)[22]; Brazil, 1967-8[21], 1987[23] & 1996-8[24]; Canada, <1967[25]; Chile 1967-8 (Santiago & rural)[21]; Mexico, 1987-88[26] & 1989[27]; Panama 1967-8 (Panama City & rural)[21]; Peru, 1967-8 (Lima & rural)[21] & 2003[28]; Uruguay, 1967-7 (urban and rural)[21]; USA <1967 (Atlanta & Houston)[25]. |
| Caribbean | Haiti, 2003[29], Jamaica, 1967-8 (Kingston & rural)[21], Trinidad 1966-7[30], 1967-8 (Port au Spain & rural)[21] |
| Eastern Mediterranean (EMRO) | Bahrain, 1981[31]; Iran, 1993-95[32]; Jordan, 1982-3[33]; Kuwait, <1978[34]; Lebanon, 1980-1[35]; Morocco, 1969-70[36]; Pakistan, <1997[37] & 1999-2004[38]; Saudi Arabia, 1989[39] & 1992-93[40], Tunisia, <1970[41]; Yemen, 1985[42] & 2002-03[43] |
| European (EURO) | Czech Republic, <1967[25]; Denmark, <1967[25] &1983[44]; East Germany, 1990[45]; England, <1967[25] & 1986-7[46]; Finland, 1979[47]; France, <1967[25]; Kyrgyzstan, 2001[48]; Romania, <1989[49]; Turkey, 1998[50], 2003-04[51] & 2005[52]. |
| South East Asian (SEARO) | Bangladesh, 2004-5[53]; India, 1968 (urban & rural Delhi)[54], 1972-3 (Chandrigarh & Lucknow)[54], 1976 (Calcutta)[55], <1987 (Delhi)[56], <1990 (Delhi)[57], 1999-2000 (urban and rural Vellore)[1]; Nepal, 2008[58], Thailand, 1978[59] |
| Western Pacific, excluding China & Australia (WPRO, excluding China & Australia) | Fiji, <1973[60]; Japan, <1967 (Sapporo &Ohtsu)[25]; Malaysia, <1972[61]; Singapore, 1975-79[62], Taiwan, 1984[63] & 1984-6[64]; Central Vietnam, 2009-2010[65] |

Table : Datasets used to set up bootstrap files for the Global Burden of Disease (GBD) regions. Note that these datasets had been accepted after performing the selection procedure described in the methods.

| **GBD Region** | **Setting from which dataset(s) were collected** |
| --- | --- |
| Sub-Saharan Africa, Central | Congo, <1991[6]; Gabon, 1985[11] |
| Sub-Saharan Africa, East | Ethiopia, 1981[9] & 1994[10]; Kenya (Kilifi), 1996-9[2-3]; Madagascar, 1990-1995[13]; Mozambique, 2002[14]; Zambia, 1979-80[20] |
| Sub-Saharan Africa, Southern | South Africa, 2003[19] |
| Sub-Saharan Africa, West | Benin, 1993[5]; Cote d'Ivoire, 1975[7] & 1985-6[8]; Ghana, 1997[12]; Nigeria, <1978[15], <2002[16] & 2007-8[17]; Senegal, 1996-2001[18] |
| Caribbean | Haiti, 2003[29], Jamaica, 1967-8 (Kingston & rural)[21], Trinidad 1966-7[30], 1967-8 (Port au Spain & rural)[21] |
| Latin America, Andean | Peru, 1967-8 (Lima & rural)[21] & 2003[28] |
| Latin America, Central | Mexico, 1987-88[26] & 1989[27], Panama 1967-8 (Panama City & rural)[21] |
| Latin America, Southern | Argentina, 1967-8 (urban & rural)[21], & 1981 (Mar de Plata)[22], Chile (Santiago & rural), 1967-8[21]; Uruguay, 1967-7 (urban and rural)[21] |
| Latin America, Tropical | Brazil, 1967-8[21], 1987[23] & 1996-8[24] |
| North America, High Income | Canada, <1967[25], USA <1967 (Atlanta & Houston)[25] |
| Asia Central | Kyrgyzstan, 2001[48] |
| North Africa / Middle East | Bahrain, 1981[31]; Iran, 1993-95[32]; Jordan, 1982-3[33]; Kuwait, <1978[34]; Lebanon, 1980-81[35]; Morocco, 1969-1970[36]; Saudi Arabia, 1989[39] & 1992-93[40] Tunisia, <1970[41]; Turkey, 1998[66], 2003-4[51] & 2005[52]; Yemen, 1985[42] & 2002-03[43] |
| Europe, Eastern | Taken to be identical to those for Europe Central (Romania, <1989[49]; Czech Republic, <1967[25]), as no datasets were available from the countries in this grouping |
| Europe Central | Romania, <1989[49]; Czech Republic, <1967[25] |
| Europe, Western | Denmark, <1967[25] &1983[44]; England, 1986-87[46] & <1967[25]; East Germany, 1990[45]; Finland, 1979[47]; France, <1967[25]. |
| Asia East | China, 1979-80[67]; Taiwan, 1984[63] & 1984-6[64] |
| Asia, South | Bangladesh 2004-5[53]; India, 1968 (urban & rural Delhi)[54], 1972-3 (Chandrigarh & Lucknow)[54], 1976 (Calcutta)[55], <1987 (Delhi)[56], <1990 (Delhi)[57], 1999-2000 (urban & rural Vellore)[1]; Nepal, 2008[58], Pakistan, <1997[37] & 1999-2004[38] |
| Asia Pacific, High Income | Japan, <1967 (Ohtsu & Sapporo)[25]; Singapore, 1975-9[62] |
| Asia, Southeast | Malaysia, <1972[61]; Thailand, 1978[59]; Central Vietnam, 2009-2010[65] |
| Australasia | Australia, <1967[25] |
| Oceania | Fiji, <1973[60] |

Table : Summary of the bootstrap datasets used to estimate the CRS incidence for each country using catalytic models (countries which had not introduced RCV by 2010) or the transmission model (countries which had introduced RCV by 2010), using either the WHO regional or GBD grouping to assign datasets for countries without serological datasets from before the introduction of RCV. See Table C and Table D for the datasets used to make up the bootstrap datasets. See Table H and Table I for estimates of the prevaccination force of infection and CRS incidence. Countries which had not introduced RCV by 2010 are shaded in grey.

| **Country** | **Bootstrap dataset used:** | |
| --- | --- | --- |
|  | **WHO regional grouping** | **GBD grouping** |
| **Africa** |  |  |
| Algeria | AFRO region | North Africa, Middle East |
| Angola | AFRO region | Sub-Saharan Africa, Central |
| Benin | Benin, 1993[5] | Benin, 1993[5] |
| Botswana | AFRO region | Sub-Saharan Africa, Southern |
| Burkina Faso | AFRO region | Sub-Saharan Africa, West |
| Burundi | AFRO region | Sub-Saharan Africa, East |
| Cameroon | AFRO region | Sub-Saharan Africa, West |
| Cape Verde | AFRO region | Sub-Saharan Africa, West |
| Central African Republic | AFRO region | Sub-Saharan Africa, Central |
| Chad | AFRO region | Sub-Saharan Africa, West |
| Comoros | AFRO region | Sub-Saharan Africa, East |
| Congo | AFRO region | Sub-Saharan Africa, Central |
| Côte d'Ivoire | Cote d'Ivoire, 1975[7] & 1985-6[8] | Cote d'Ivoire, 1975[7] & 1985-6[8] |
| Democratic Republic of the Congo | AFRO region | Sub-Saharan Africa, Central |
| Equatorial Guinea | AFRO region | Sub-Saharan Africa, Central |
| Eritrea | AFRO region | Sub-Saharan Africa, East |
| Ethiopia | Ethiopia, 1981[9] & 1994[10] | Ethiopia, 1981[9] & 1994[10] |
| Gabon | Gabon, 1985[11] | Gabon, 1985[11] |
| Gambia | AFRO region | Sub-Saharan Africa, West |
| Ghana | Ghana, 1997[12] | Ghana, 1997[12] |
| Guinea | AFRO region | Sub-Saharan Africa, West |
| Guinea-Bissau | AFRO region | Sub-Saharan Africa, West |
| Kenya | Kenya (Kilifi), 1996-9[2-3] | Kenya (Kilifi), 1996-9[2-3] |
| Lesotho | AFRO region | Sub-Saharan Africa, Southern |
| Liberia | AFRO region | Sub-Saharan Africa, West |
| Madagascar | Madagascar, 1990-1995 [13] | Madagascar, 1990-1995 [13] |
| Malawi | AFRO region | Sub-Saharan Africa, East |
| Mali | AFRO region | Sub-Saharan Africa, West |
| Mauritania | AFRO region | Sub-Saharan Africa, West |
| Mauritius | AFRO region | Asia, South East |
| Mozambique | Mozambique, 2002[14] | Mozambique, 2002[14] |
| Namibia | AFRO region | Sub-Saharan Africa, Southern |
| Niger | AFRO region | Sub-Saharan Africa, West |
| Nigeria | AFRO region | Sub-Saharan Africa, West |
| Réunion | AFRO region | Asia, South East |
| Rwanda | AFRO region | Sub-Saharan Africa, East |
| Sao Tome and Principe | AFRO region | Sub-Saharan Africa, West |
| Senegal | Senegal, 1996-2001 [18] | Senegal, 1996-2001 [18] |

| **Country** | **Bootstrap dataset used:** | |
| --- | --- | --- |
|  | **WHO regional grouping** | **GBD grouping** |
| Sierra Leone | AFRO region | Sub-Saharan Africa, West |
| South Africa | South Africa, 2003 [19] | South Africa, 2003 [19] |
| Swaziland | AFRO region | Sub-Saharan Africa, Southern |
| Togo | AFRO region | Sub-Saharan Africa, West |
| Uganda | AFRO region | Sub-Saharan Africa, East |
| United Republic of Tanzania | AFRO region | Sub-Saharan Africa, East |
| Western Sahara | AFRO region | North Africa, Middle East |
| Zambia | Zambia, 1979-80 [20] | Zambia, 1979-80 [20] |
| Zimbabwe | AFRO region | Sub-Saharan Africa, Southern |
| **Americas** |  |  |
| Argentina | Argentina, 1967-8 (urban & rural)[21], & 1981 (Mar de Plata)[22] | Argentina, 1967-8 (urban & rural)[21], & 1981 (Mar de Plata)[22] |
| Aruba | Caribbean | Caribbean |
| Bahamas | Caribbean | Caribbean |
| Barbados | Caribbean | Caribbean |
| Belize | Caribbean | Caribbean |
| Bolivia | AMRO region, excluding the Caribbean | Latin America, Andean |
| Brazil | Brazil, 1967-8[21], 1987[23] & 1996-8[24] | Brazil, 1967-8[21], 1987[23] & 1996-8[24] |
| Canada | Canada, <1967[25] | Canada, <1967[25] |
| Chile | Chile 1967-8 (Santiago & rural)[21] | Chile 1967-8 (Santiago & rural)[21] |
| Colombia | AMRO region, excluding the Caribbean | Latin America, Central |
| Costa Rica | AMRO region, excluding the Caribbean | Latin America, Central |
| Cuba | Caribbean | Caribbean |
| Dominican Republic | Caribbean | Caribbean |
| Ecuador | AMRO region, excluding the Caribbean | Latin America, Andean |
| El Salvador | AMRO region, excluding the Caribbean | Latin America, Central |
| French Guiana | Caribbean | Caribbean |
| Grenada | Caribbean | Caribbean |
| Guadeloupe | Caribbean | Caribbean |
| Guatemala | AMRO region, excluding the Caribbean | Latin America, Central |
| Guyana | Caribbean | Caribbean |
| Haiti | Haiti, 2003[29] | Haiti, 2003[29] |
| Honduras | AMRO region, excluding the Caribbean | Latin America, Central |
| Jamaica | Jamaica, 1967-8 (Kingston & rural)[21] | Jamaica, 1967-8 (Kingston & rural)[21] |
| Martinique | Caribbean | Caribbean |
| Mexico | Mexico, 1987-88[26] & 1989[27] | Mexico, 1987-88[26] & 1989[27] |
| Netherlands Antilles | Caribbean | Caribbean |
| Nicaragua | AMRO region, excluding the Caribbean | Latin America, Central |
| **Country** | **Bootstrap dataset used:** | |
|  | **WHO regional grouping** | **GBD grouping** |
| Panama | Panama 1967-8 (Panama City & rural)[21] | Panama 1967-8 (Panama City & rural)[21] |
| Paraguay | AMRO region, excluding the Caribbean | Latin America, Tropical |
| Peru | Peru, 1967-8 (Lima & rural)[21] & 2003[28] | Peru, 1967-8 (Lima & rural)[21] & 2003[28] |
| Puerto Rico | USA (Atlanta and Houston), <1967[25] | USA (Atlanta and Houston), <1967[25] |
| Saint Lucia | Caribbean | Caribbean |
| Saint Vincent and the Grenadines | Caribbean | Caribbean |
| Suriname | Caribbean | Caribbean |
| Trinidad and Tobago | Trinidad 1966-7[30], 1967-8 (Port au Spain & rural)[21] | Trinidad 1966-7[30], 1967-8 (Port au Spain & rural)[21] |
| USA | USA <1967 (Atlanta & Houston)[25] | USA <1967 (Atlanta & Houston)[25] |
| US Virgin Islands | USA <1967 (Atlanta & Houston)[25] | USA <1967 (Atlanta & Houston)[25] |
| Uruguay | Uruguay, 1967-7 (urban and rural)[21] | Uruguay, 1967-7 (urban and rural)[21] |
| Venezuela | AMRO region, excluding the Caribbean | Latin America, Central |
| **Eastern Mediterranean** |  |  |
| Afghanistan | EMRO region | Asia, South |
| Bahrain | Bahrain, 1981[31] | Bahrain, 1981[31] |
| Djibouti | EMRO region | Sub-Saharan Africa, East |
| Egypt | EMRO region | North Africa / Middle East |
| Iran | Iran, 1993-95[32] | Iran, 1993-95[32] |
| Iraq | EMRO region | North Africa / Middle East |
| Jordan | Jordan, 1982-3[33] | Jordan, 1982-3[33] |
| Kuwait | Kuwait, <1978[34] | Kuwait, <1978[34] |
| Lebanon | Lebanon, 1980-81[35] | Lebanon, 1980-81[35] |
| Libya | EMRO region | North Africa / Middle East |
| Morocco | Morocco, 1969-1970[36] | Morocco, 1969-1970[36] |
| Oman | EMRO region | North Africa / Middle East |
| Pakistan | Pakistan, <1997[37] & 1999-2004[38] | Pakistan, <1997[37] & 1999-2004[38] |
| Qatar | EMRO region | North Africa / Middle East |
| Saudi Arabia | Saudi Arabia, 1989[39] & 1992-3[40] | Saudi Arabia, 1989[39] & 1992-3[40] |
| Somalia | EMRO region | Sub-Saharan Africa, East |
| Sudan | EMRO region | Sub-Saharan Africa, East |
| Syrian Arab Republic | EMRO region | North Africa/ Middle East |
| Tunisia | Tunisia, <1970[41] | Tunisia, <1970[41] |
| United Arab Emirates | EMRO region | North Africa / Middle East |
| Yemen | Yemen, 1985[42] & 2002-3[43] | Yemen, 1985[42] & 2002-3[43] |
| **Europe** |  |  |
| Albania | EURO region | Europe, Central |
| Armenia | EURO region | Asia, Central |
| Austria | EURO region | Europe, Western |
| Azerbaijan | EURO region | Asia, Central |
| **Country** | **Bootstrap dataset used:** | |
|  | **WHO regional grouping** | **GBD grouping** |
| Belarus | EURO region | Europe, Eastern |
| Belgium | EURO region | Europe, Western |
| Bosnia and Herzegovina | EURO region | Europe, Central |
| Bulgaria | EURO region | Europe, Central |
| Channel Islands | EURO region | Europe, Western |
| Croatia | EURO region | Europe, Central |
| Cyprus | EURO region | Europe, Western |
| Czech Republic | Czech Republic, <1967[25] | Czech Republic, <1967[25] |
| Denmark | Denmark, <1967[25] &1983[44] | Denmark, <1967[25] &1983[44] |
| Estonia | EURO region | Europe, Eastern |
| Finland | Finland, 1979[47] | Finland, 1979[47] |
| France | France, <1967[25] | France, <1967[25] |
| Georgia | EURO region | Asia, Central |
| Germany | East Germany, 1990[45] | East Germany, 1990[45] |
| Greece | EURO region | Europe, Western |
| Hungary | EURO region | Europe, Central |
| Iceland | EURO region | Europe, Western |
| Ireland | EURO region | Europe, Western |
| Israel | EURO region | Europe, Western |
| Italy | EURO region | Europe, Western |
| Kazakhstan | EURO region | Asia, Central |
| Kyrgyzstan | Kyrgyzstan, 2001[48] | Kyrgyzstan, 2001[48] |
| Latvia | EURO region | Europe, Eastern |
| Lithuania | EURO region | Europe, Eastern |
| Luxembourg | EURO region | Europe, Western |
| Malta | EURO region | Europe, Western |
| Montenegro | EURO region | Europe, Central |
| Netherlands | EURO region | Europe, Western |
| Norway | EURO region | Europe, Western |
| Poland | EURO region | Europe, Central |
| Portugal | EURO region | Europe, Western |
| Moldova | EURO region | Europe, Eastern |
| Romania | Romania, <1989[49] | Romania, <1989[49] |
| Russia | EURO region | Europe, Eastern |
| Serbia | EURO region | Europe, Central |
| Slovakia | EURO region | Europe, Central |
| Slovenia | EURO region | Europe, Central |
| Spain | EURO region | Europe, Western |
| Sweden | EURO region | Europe, Western |
| Switzerland | EURO region | Europe, Western |
| Macedonia | EURO region | Europe, Central |
| Tajikistan | EURO region | Asia, Central |
| Turkey | Turkey, 1998[50], 2003-04[51] & 2005[52] | Turkey, 1998[50], 2003-04[51] & 2005[52] |
| Turkmenistan | EURO region | Asia, Central |
| Ukraine | EURO region | Europe, Eastern |
| United Kingdom | England, <1967[25] & 1986-87[46] | England, <1967[25] & 1986-87[46] |
| Uzbekistan | EURO region | Asia, Central |
| **Country** | **Bootstrap dataset used:** | |
|  | **WHO regional grouping** | **GBD grouping** |
| **South East Asia** |  |  |
| Bangladesh | Bangladesh, 2004-5[53] | Bangladesh, 2004-5[53] |
| Bhutan | SEARO region | Asia, South |
| India | India, 1968 (urban & rural Delhi)[54], 1972-3 (Chandrigarh & Lucknow)[54], 1976 (Calcutta)[55], <1987 (Delhi)[56], <1990 (Delhi)[57], 1999-2000 (urban and rural Vellore)[1] | India, 1968 (urban & rural Delhi)[54], 1972-3 (Chandrigarh & Lucknow)[54], 1976 (Calcutta)[55], <1987 (Delhi)[56], <1990 (Delhi)[57], 1999-2000 (urban and rural Vellore)[1] |
| Indonesia | SEARO region | Asia, Southeast |
| Maldives | SEARO region | Asia, Southeast |
| Myanmar | SEARO region | Asia, Southeast |
| Nepal | Nepal, 2008[58] | Nepal, 2008[58] |
| Sri Lanka | SEARO region | Asia, Southeast |
| Thailand | Thailand, 1978[59] | Thailand, 1978[59] |
| Timor-Leste | SEARO region | Asia, Southeast |
| **Western Pacific** |  |  |
| Australia | Australia, <1967[25] | Australia, <1967[25] |
| Brunei Darussalam | WPRO region, excluding China & Australia | Asia Pacific, High Income |
| Cambodia | WPRO region, excluding China & Australia | Asia, South East |
| China | China, 1979-80[67] | China, 1979-80[67] |
| China (Hong Kong) | China, 1979-80[67] | China, 1979-80[67] |
| China (Macao) | China, 1979-80[67] | China, 1979-80[67] |
| Fiji | Fiji, <1973[60] | Fiji, <1973[60] |
| French Polynesia | WPRO region, excluding China & Australia | Oceania |
| Guam | WPRO region, excluding China & Australia | Oceania |
| Japan | Japan, <1967 (Sapporo &Ohtsu)[25] | Japan, <1967 (Sapporo &Ohtsu)[25] |
| Laos | WPRO region, excluding China & Australia | Asia, Southeast |
| Malaysia | Malaysia, <1972[61] | Malaysia, <1972[61] |
| Micronesia | WPRO region, excluding China & Australia | Oceania |
| Micronesia (Fed. States) | WPRO region, excluding China & Australia | Oceania |
| Mongolia | WPRO region, excluding China & Australia | Asia, Central |
| New Caledonia | WPRO region, excluding China & Australia | Oceania |
| New Zealand | Australia, <1967[25] | Australia, <1967[25] |
| Papua New Guinea | WPRO region, excluding China & Australia | Oceania |
| Philippines | WPRO region, excluding China & Australia | Asia, Southeast |
| Polynesia | WPRO region, excluding China & Australia | Oceania |

| **Country** | **Bootstrap dataset used:** | |
| --- | --- | --- |
|  | **WHO regional grouping** | **GBD grouping** |
| Republic of Korea | WPRO region, excluding China & Australia | Asia Pacific, high income |
| Samoa | WPRO region, excluding China & Australia | Oceania |
| Singapore | Singapore, 1975-9[62] | Singapore, 1975-9[62] |
| Solomon Islands | WPRO region, excluding China & Australia | Oceania |
| Tonga | WPRO region, excluding China & Australia | Oceania |
| Vanuatu | WPRO region, excluding China & Australia | Oceania |
| Vietnam | Central Vietnam, 2009-2010[65] | Central Vietnam, 2009-2010[65] |

# C: Description of the transmission model

## Model structure

Figure A summarises the general structure of the model. The population is stratified into those who have maternal immunity, those who are susceptible, pre-infectious (infected but not yet infectious), infectious and immune. The demography in the model was described using a realistic age structured (RAS) population[68], with individuals stratified by sex and into 75 age strata, corresponding to the ages <1, 1-<2, 2-<3, …, 74-<75 years.

Single year age strata were used in preference to strata involving a wide age range, in order to avoid introducing inaccuracies when ageing the population from one age group into the next. For example, if the population were to be merged into compartments comprising 5 year age groups, we would need to apply an ageing rate to each group in order to move the population from one age group to the next. This is not ideal since we would be applying the ageing rate to those who had just moved into the compartment. This could result in some of those aged 30 years, for example, entering the 35-39 year old compartment within days of them becoming 30 years old. To increase consistency with the age grouping available for the mortality rates and therefore the age distribution in the population, we have considered all those aged over 75 years, as opposed to those aged over 70 years, as a single group.

For countries with a population growth rate of <2%/year, the age structure was assumed to be rectangular, with a constant birth rate over time, 10000 people in each single year age category and with individuals surviving until age 75 years before dying. For growing populations (growth rate >2%/year), we used the country-and age-specific mortality rate, calculated from survival data for the period 2005-2010 from UN population databases.[69] Therefore, the number of people of age *a* at a given time *t* *N_a_(t)* depends on the mortality rate. The number of births in the model was calculated by multiplying model predictions of the population size in the given year by the crude annual per capita birth rate for the period 2005-2010, obtained from UN population databases. Both the mortality and crude birth rates in the model were assumed to be fixed over time. Note that the absolute magnitude of the numbers of births in the model does not greatly influence the absolute numbers of CRS cases predicted for the global burden, since these were calculated by multiplying model predictions of the age-specific number of CRS cases per live birth by the observed numbers of live births by maternal age, as seen in UN population databases[69] and then summing the resulting numbers over all maternal ages.

Individuals are born into the first age stratum (stratum *a*=0) on the 31^st^ August of each year and are assumed to have maternal immunity for 6 months. Although this approach for introducing newborns into the population may be less “natural” than allowing newborns to enter the first age stratum continuously over time, it facilitates tracking the exact time when individuals in the model are aged 6 months, when they lose maternal immunity and can first be vaccinated. Following standard approaches[68] individuals in each age stratum move to the subsequent age stratum on the 31^st^ August of each year, at the same time as vaccination occurs (see below), and leave the model once they reach age 75 years.

The force of infection in the model at a given time *t* (*λ_y_(t)* and *λ_o_(t))* depends on age-specific contact between people and the prevalence of infectious people, with the contact parameters calculated from average annual force of infection estimates from seroprevalence data (see section 3.3).

For convenience, vaccination is implemented on a single day each year in the model, which is the simplest and least computationally intensive way of ensuring that the intended coverage is attained. For example, if we were to assume that vaccination campaigns are carried out over a period of a few weeks, we would need to keep track of how many individuals have been vaccinated in each time step in the model, and keep updating how many still need to be vaccinated in order to attain the required coverage. Estimates of the overall burden of CRS obtained by introducing vaccination on a single day should be similar to that obtained by implementing vaccination with the same overall coverage over a period of weeks or months.

We note that an SEIR model could have also been used to calculate the CRS burden for countries which had not introduced RCV by 2010. However, doing so would have increased the computational burden but would not have affected the results. For example, the force of infection estimated using the catalytic model was used to calculate the contact parameters which were included in the transmission model and the latter, in turn, would have reproduced the same force of infection that was used to calculate the contact parameters in the transmission model.

We also note that the model does not include any effects of seasonality or metapopulation dynamics. Data on these effects are limited and including their effects would have increased the computational burden without affecting the results. For example, the overall effect of seasonality would be to increase the predicted CRS incidence in approximately one half of the year and decrease it in the other half of the year but the overall average annual CRS incidence, which is the value that of interest in these analyses, is unaffected. Likewise, including metapopulation dynamics, would result in increased estimates in some parts of each country, and decrease them elsewhere, with the overall estimates remaining similar.

Table F and Table G give definitions of the variables and parameters respectively that are used in the model. Throughout the description, we use the subscript “*y”* to refer to younger individuals (aged <13 years) and the subscript “*o”* to refer to older individuals (aged ≥13 years). Where necessary, the subscript “w” is used to denote females.


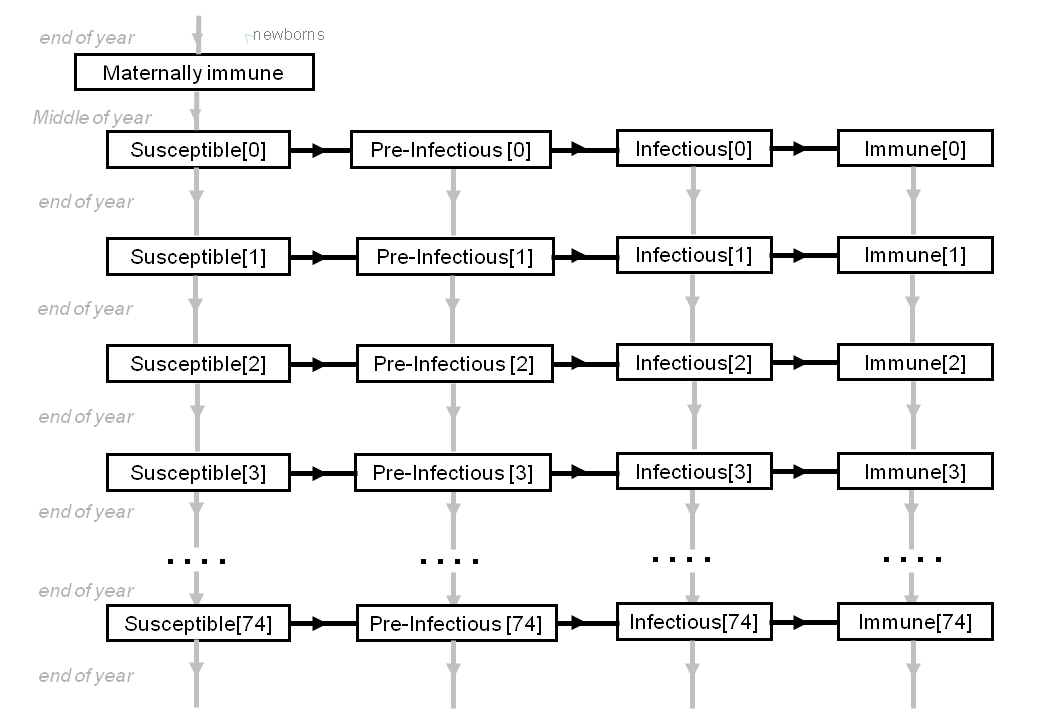


Figure : General structure of the transmission model used to recreate the epidemiology of rubella, before the introduction of vaccination.

Table F**:** Summary of the definitions of compartments and variables used in the model. Where necessary in the equations, the subscript “w” is used to denote females.

| **Variable** | **Definition** |
| --- | --- |
| *M_g_(t)* | Number of individuals of gender *g* with maternal immunity at time *t*. |
| *S_a,g_(t)* | Number of susceptible individuals of gender *g* aged *a* years at time *t.* |
| *E_a,g_(t)* | Number of individuals in the pre-infectious category (infected but not infectious) of gender *g* and age *a* years at time *t.* |
| *I_a,g_(t)* | Number of infectious individuals of gender *g* and aged *a* years at time *t.* |
| *I_y_(t), I_o_(t)* | Number of younger and older infectious individuals at time *t.* |
| $\overline{I}_{y}$, $\overline{I}_{o}$ | Average number of younger and older infectious individuals before the introduction of vaccination. |
| *R_a,g_(t)* | Number of individuals of gender *g* and aged *a* years at time *t* who are immune either as a result of vaccination or natural infection. |
| *N_a_(t)* | Total number of people (males and females combined) aged *a* at time *t*. |
| *N_y_(t), N_o_(t)* | Total number of younger and older individuals at time *t* (aged <13 and ≥13 years respectively). |

Table **:** Summary of the definitions of the transition-related parameters used in the analyses

| **Parameter** | **Definition** |
| --- | --- |
| *λ_a_(t), λ_y_(t), λ_o_(t)* | The force of infection for individuals in a given age group at time *t*. The subscript *a* refers to individuals of age *a;* the subscripts *y* (‘younger’) and *o* (‘older’) refer to individuals aged <13 and ≥13 years respectively. |
| $\overline{\lambda}_{y}$, $\overline{\lambda}_{o}$ | The average force of infection before the introduction of vaccination for individuals aged <13 years (‘younger’) and ≥13 years (‘older’), respectively. |
| *β_yo_* | The rate at which specific younger susceptible individuals come into effective contact with older infectious persons per unit time. An effective contact is defined as one which is sufficient to lead to transmission between an infectious and susceptible individual[70]. The definitions of *β_oy_, β_oo_, β_yy_* are analogous. |
| *c_yo_* | The number of younger susceptible individuals effectively contacted by each older infectious person per unit time. An effective contact is defined as one which is sufficient to lead to transmission between an infectious and susceptible individual[70]. The definitions of *c_oy_, c_oo_, c_yy_* are analogous. |
| *m_a_* | The mortality rate for individuals of age *a*. For populations which are assumed to be stable over time, the mortality rate is assumed to be zero until age 75 years, when all individuals leave the model. For growing countries, the rate is calculated using survival data for 2005-2010 from UN population databases[69]. |
| *v_a,g_(t)* | The proportion of individuals of age *a* of gender *g* who are vaccinated at time *t*. The coverage data are those estimated and/or reported to WHO and supplemented by the literature, where available. |
| *B_g_(t)* | The number of live births each year for males or females in the model. Assumed to be 2500 per year for populations which are assumed to remain the same size over time. For countries in which the population is increasing over time, the numbers of live births was calculated as the product of the predicted population size and the crude per capita birth rate, obtained from UN population databases[69]. Note that the absolute magnitude of the numbers of births in the model does not greatly influence the absolute numbers of CRS cases predicted for the global burden, since these were calculated by multiplying model predictions of the age-specific number of CRS cases per live birth by the observed numbers of live births by maternal age, as seen in UN population databases[69] and then summing the resulting numbers over all maternal ages. |
| *f* | The rate at which individuals in the pre-infectious category become infectious, taken to equal 0.1/day, equivalent to assuming an average pre-infectious period of 10 days. |
| *r* | The rate at which infectious individuals recover and become immune, taken to equal 0.909 per day, equivalent to assuming an average infectious period of 11 days. |
| *T_crs_* | Time period during which there is an increased risk of the child being born with CRS, if the mother is infected whilst pregnant. |
| *T_E_* | Last year of the model simulations, 2010 |

## Model equations

The equations used in the transmission model depend on whether the model described the transmission dynamics in a stable population with a rectangular age distribution or one in which the population size was assumed to increase over time. The differential equations describing the transmission of rubella in growing populations in age stratum *a* (a=0, 1, 2, ...,74 years) are provided below (see Table F and Table G for the definitions of variables and parameters). The corresponding equations considering a population which is assumed to remain stable over time are identical, except that the mortality rate for all age strata (*m_a_(t)*) is assumed to be zero until individuals reach age 75 years, when they leave the model.

$$\begin{matrix} \frac{dM_{g}(t)}{dt}=-m_{0}M_{g}(t) 0<t<182 \mathrm{mod} 365 \\ \frac{dS_{a,g}(t)}{dt}=-\lambda_{a}(t)S_{a,g}(t)-m_{a}S_{a,g}(t) \\ \frac{dE_{a,g}(t)}{dt}=\lambda_{a}(t)S_{a,g}(t)-m_{a}E_{a,g}(t)-fE_{a,g}(t) \\ \frac{dI_{a,g}(t)}{dt}=fE_{a,g}(t)-m_{a}I_{a,g}(t)-rI_{a,g}(t) \\ \frac{dR_{a,g}(t)}{dt}=rI_{a,g}(t)-m_{a}R_{a,g}(t) \end{matrix}$$

The equations for the transitions occurring on 31^st^ August (denoted by *T*) each year are as follows:

*M_g_(T) = B_g_(T)*

*S_a,g_(T) = S_a-1,g_(T-δt)(1-v_a,g_-m_a-1_-λ_a-1_(T-δt)) for 0< a <74* years

*E_a,g_(T) = E_a-1,g_(T-δt)(1-m_a-1_ -f)+λ_a-1_(T-δt)S_a-1,g_(T-δt) for 0< a <74* years

*I_a,g_(T) = I_a-1,g_(T-δt)(1-m_a-1_-r)+f E_a-1,g_(T-δt) for 0< a <74* years

*R_a,g_(T) = R_a-1,g_(T-δt)(1-m_a-1_-r)+rI_a-1,g_(T-δt)+ v_a,g_ S_a-1,g_(T-δt) for 0< a ≤75* years

The equations for the transitions occurring 6 months after the 31^st^ August (or equivalently, 28^th^ February), when individuals in the first year of life lose their maternal immunity are:

*S_0,g_(T) = M_g_(T-δt)(1-m_0_-v_0.5,g_)*

*M_g_(T) = 0*

The equations were solved using a specially written C-program, using the Euler method with a time step, *δt,* of 0.25 day. The Euler method was used in preference to the Runge-Kutta method to facilitate movement of each age stratum in the model into the next at the end of each year. For example, following the standard approach of Schenzle (1984)[68], individuals in each age stratum are moved into the next age stratum at the end of each year, which is straightforward to implement if the equations are solved using the Euler method. However it is not straightforward to implement using the Runge Kutta method, for which the average value over various steps (depending on the variant of the method used) is used when integrating the equations. Given the small time step used (0.25 days) predictions based on the Euler method will be very similar to those based on the Runge-Kutta method.

The model was run for 170 simulated years before the introduction of RCV, starting from the equilibrium numbers of individuals in each compartment, and with a population size of 750,000 (*N(T_0_)*), with equal numbers of males and females.

The number of CRS cases per 100,000 live births occurring among mothers in age group *A_j,k_* (spanning the age groups *a_j_-a_k_*) at time *t* was calculated using the model prediction of the daily force of infection at time *t* using the following equation:

| $I_{CRS}(A_{j,k},t)=\frac{\sum_{a=a_{j}}^{a_{k}} S_{a,w}(t)(1-e^{-T_{CRS}\lambda_{o}(t)})\times0.65\times100000}{\sum_{a=a_{j}}^{a_{k}} N_{a,w}(t)}$ |  |
| --- | --- |

The number of CRS cases per 100,000 live births in a given year occurring among mothers in a given age group was calculated by taking the average of the values in each time step in the year.

The equation can be derived by first assuming that both infection with rubella and the proportion of women at a given age that are susceptible are independent of whether or not a woman is pregnant. The proportion of pregnant women in a given age group A_j,k_ at a given time that are infected during the first 16 weeks of pregnancy is therefore the same as the proportion of women of that age that are susceptible ($\sum_{a=a_{j}}^{a_{k}} S_{a,w}(t)/\sum_{a=a_{j}}^{a_{k}} N_{a,w}(t)$) multiplied by the average risk of infection during a 16 week period $(1-e^{-T_{CRS}\lambda_{o}(t)})$. Multiplying the resulting expression by the risk that a child is born with CRS, if the mother is infected during the first 16 weeks of pregnancy (0.65), we obtain an estimate of the proportion of pregnant women of age *a* at time *t* whose pregnancies (or equivalently, live births) result in a child with CRS. Summing over all time steps in a year, we obtain the proportion of all pregnancies (or live births) at age *a* in that year that result in children born with CRS.

## Contact parameters in the model

The contact parameters in the model were assumed to differ between younger and older individuals according to the following matrix of “Who Acquired Infection From Whom”:

$$\begin{matrix} \leq13yrs >13yrs \\ \begin{matrix} \leq13yrs \\ >13yrs \end{matrix}\left(\begin{matrix} \beta_{1} & 0.7\beta_{2} \\ 0.7\beta_{2} & \beta_{2} \end{matrix} \right) \end{matrix}$$

For this matrix, the rate at which those aged <13 years come into effective contact with others of the same age (*β_1_*) differs from the rate at which older individuals come into effective contact with other older individuals (*β_2_*). An effective contact is defined as one that is sufficient to lead to transmission if it occurs between a susceptible and infectious person[70]. The rate at which younger and older individuals come into effective contact is assumed to be 70% of the rate at which older individuals effectively contact each other, which is consistent with empirical data from middle-income settings[71], which are typical of those which were likely to have introduced rubella-containing vaccination. Recent studies suggest that the rate at which children and adults contact each other estimated in these studies is likely to be similar elsewhere[72].

The contact parameters in the model for each country were calculated for each bootstrap estimate for the force of infection for younger and older individuals, before the introduction of vaccination using standard methods (see below)[73]. For a given assumption about contact between individuals, the force of infection at time *t* for individuals among younger and older individuals (*λ_y_(t)* and *λ_o_(t)* respectively*),* is given by the following equations:

| Stable populations with rectangular age distribution: | $\begin{matrix} \lambda_{y}(t)=\beta_{yy}I_{y}(t)+\beta_{yo}I_{o}(t) \\ \lambda_{o}(t)=\beta_{oy}I_{y}(t)+\beta_{oo}I_{o}(t) \end{matrix}$ |
| --- | --- |
| Growing populations: | $\begin{matrix} \lambda_{y}(t)=\frac{c_{yy}I_{y}(t)+c_{yo}I_{o}(t)}{N_{y}(t)} \\ \lambda_{o}(t)=\frac{c_{oy}I_{y}(t)+c_{oo}I_{o}(t)}{N_{o}(t)} \end{matrix}$ |

*c_yy_, c_yo_, c_oy_* and *c_oo_* are related to *β_yy_, β_yo_, β_oy_* and *β_oo_* through the following equations, where *T_0_* is the start of the model runs:

$$\begin{matrix} c_{yy}=\beta_{yy}N_{y}(T_{0}) \\ c_{yo}=\beta_{yo}N_{y}(T_{0}) \\ c_{oy}=\beta_{oy}N_{o}(T_{0}) \\ c_{oo}=\beta_{oo}N_{o}(T_{0}) \end{matrix}$$

The parameters, *β_1_* and *β_2_* in the WAIFW matrix for given values for the average force of infection before the introduction of vaccination among younger and older individuals for a given country (denoted by $\bar{\lambda}_{y}$ and $\bar{\lambda}_{o}$ respectively) were calculated using the following equations:

where $\overline{I}_{y}$ and $\overline{I}_{o}$ are the average numbers of infectious individuals (males and females combined) for younger and older individuals, respectively. These equations are obtained after rearranging the following equation, which relates the force of infection to the number of infectious younger and older individuals:

The calculations of the contact parameters implicitly assume that the seroprevalence data were representative of the seroprevalence in the general population at any given time, and therefore that the average annual force of infection and contact parameters estimated from these data are also representative of those in the general population. $\overline{I}_{y}$ and $\overline{I}_{o}$ are calculated using the approximations and , where *D* is the duration of infectiousness and $\overline{S}_{y}$ and $\overline{S}_{o}$are the average numbers of susceptible children and older individuals respectively. For populations with a rectangular age distribution, $\overline{S}_{y}$ and $\overline{S}_{o}$ are given by:

where *N* is the total population size, *a_y_* (=13 years) is the oldest age of young individuals and *L* is the life expectancy (75 years).

For growing populations, the equations are as follows:

Here, the number of people of age *a* was calculated using the following equation, namely by multiplying the population size at the start (*N_a_(T_0_)*) by the proportion of the population at equilibrium that was of age *a:*

$$N_{a}(T_{0})=N(T_{0})\frac{N_{a}(T_{E})}{\sum_{a=0}^{75} N_{a}(T_{E})}$$

The equilibrium numbers of people in each age group were obtained by running the model until it reached 2010 and therefore takes account of age-dependent mortality rates and the survival of people.

## Vaccination coverage data

The vaccination coverage data that were used in the model are described in the main text. Missing SIA or routine coverage data were further supplemented from publications[74-93]. Data on “selective vaccination” coverage came from publications where possible[94-96] (see main text for details).

# D: Results from fitting catalytic models to the serological data collected before the introduction of RCV

#

Table : Summary of the studies, best-fitting values for the force of infection and (where appropriate) the sensitivity of the antibody assay, and the CRS incidence per 100,000 live births for each catalytic model, for settings in which rubella vaccine had not been introduced by 2010. The values in parentheses reflect the 95% confidence intervals, obtained by bootstrapping. To facilitate comparisons, the CRS incidence is not weighted by the number of live births.

| **Country, year of study** | **Study population** | **Sample size (no. of age groups)** | **Lab test (cut-off)** | **Cata-lytic model** | **Force of infection (/1000/year)** | | **Sensitivity (%)** | **CRS/ 100,000 live births** | **Loglike-lihood deviance (deg of freedom)** | **AICc** | **Selected model based on criterion:** | |
| --- | --- | --- | --- | --- | --- | --- | --- | --- | --- | --- | --- | --- |
|  |  |  |  |  | **<13 yr olds** | **≥13 yr olds** |  |  |  |  | **1** | **2** |
| **Africa** | | | | | | | | | | | | |
| Benin, 1993[5] | Pregnant F | 211 (4) | HAI-? | A | 0 (0,968) | 652 (0,928) | 86 (82,100) | 170 (0,391) | 0.3 (1) | -- | D | D |
|  |  |  |  | B | 156 (91,181) | 0 (0,52) | - | 0 (0,149) | 0.4 (2) | 29 |  |  |
|  |  |  |  | C | 329 (89,985) | 329 (89,985) | 86 (81,94) | 6 (0,173) | 0.4 (2) | 29 |  |  |
|  |  |  |  | D | 69 (58,82) | 69 (58,82) | - | 217 (187,240) | 6.9 (3) | 23 |  |  |
| Congo, <1991[6] | Pregnant F | 100 (6) | HAI-1:20 | A | 0 (0,214) | 378 (59,833) | 89 (82,100) | 306 (6,420) | 3.6 (3) | 36 | B | D |
|  |  |  |  | B | 118 (12,184) | 32 (0,161) | - | 88 (0,408) | 7.2 (4) | 30 |  |  |
|  |  |  |  | C | 121 (76,769) | 121 (76,769) | 90 (82,100) | 113 (0,202) | 5.7 (4) | 28 |  |  |
|  |  |  |  | D | 75 (59,99) | 75 (59,99) | - | 205 (153,238) | 8.3 (5) | 26 |  |  |
| Cote d'Ivoire, 1975[7] | Pregnant F | 4952 (5) | HAI-1:10 | A | 131 (86,148) | 183 (39,504) | 89 (86,100) | 90 (62,127) | 2.2 (2) | 63 | B | D |
|  |  |  |  | B | 116 (105,127) | 48 (32,64) | -- | 110 (81,136) | 3.7 (3) | 44 |  |  |
|  |  |  |  | C | 135 (115,162) | 135 (115,162) | 90 (88,94) | 94 (63,123) | 2.3 (3) | 43 |  |  |
|  |  |  |  | D | 90 (87,94) | 90 (87,94) | -- | 171 (163,177) | 31.1 (4) | 65 |  |  |
| Cote d'Ivoire, 1984-6[97] | Pregnant F | 1143 (3) | HAI:1:20 | A | 447 (66,991) | 0 (0,860) | 60 (57,100) | 0 (0,56) | 1.3 (0) | -- | D  dropped –poor fit | D dropped –poor fit |
|  |  |  |  | B | 72 (65,77) | 0 (0,6) | -- | 0 (0,48) | 1.3 (1) | -- |  |  |
|  |  |  |  | C | 925 (174,999) | 925 (174,999) | 59 (57,62) | 0 (0,54) | 1.3 (1) | -- |  |  |
|  |  |  |  | D | 35 (32,37) | 35 (32,37) | -- | 263 (261,264) | 58 (2) | 82 |  |  |

| **Country, year of study** | **Study population** | **Sample size (no. of age groups)** | **Lab test (cut-off)** | **Cata-lytic model** | **Force of infection (/1000/year)** | | **Sensitivity (%)** | **CRS/ 100,000 live births** | **Loglike-lihood deviance (deg of freedom)** | **AICc** | **Selected model based on criterion:** | |
| --- | --- | --- | --- | --- | --- | --- | --- | --- | --- | --- | --- | --- |
|  |  |  |  |  | **<13 yr olds** | **≥13 yr olds** |  |  |  |  | **1** | **2** |
| Cote d'Ivoire, 1985-6[8] | Random sera samples M & F | 2524 (9) | HAI-1:10 | A | 156 (74,191) | 1000 (0,1000) | 61 (60,100) | 11 (0,44) | 12.3 (6) | 74 | C | C |
|  |  |  |  | B | 75 (70,79) | 0 (0,5) | -- | 0 (0,37) | 14.5 (7) | 72 |  |  |
|  |  |  |  | C | 170 (140,215) | 170 (140,215) | 62 (60,65) | 57 (29,87) | 16.5 (7) | 74 |  |  |
|  |  |  |  | D | 38 (36,40) | 38 (36,40) | -- | 264 (263,264) | 170 (8) | 224 |  |  |
| Ethiopia , 1981[9] | Pregnant F | 137 (6) | HAI-1:16 | A | 137 (0,264) | 99 (1,989) | 100 (92,100) | 95 (0,275) | 2.2 (3) | 33 | B | D |
|  |  |  |  | B | 137 (29,241) | 99 (0,295) | -- | 95 (0,253) | 2.2 (4) | 23 |  |  |
|  |  |  |  | C | 122 (100,997) | 122 (100,997) | 100 (91,100) | 113 (0,151) | 2.3 (4) | 23 |  |  |
|  |  |  |  | D | 122 (98,162) | 122 (98,162) | -- | 113 (64,155) | 2.3 (5) | 18 |  |  |
| Ethiopia (Addis Ababa), 1994[10] | Urban F population | 2809 (50) | RH, EIA & LA | A | 261 (230,295) | 83 (23,164) | 98 (96,100) | 20 (12,28) | 75.2 (47) | 194 | A | A |
|  |  |  |  | B | 233 (215,252) | 26 (9,47) | -- | 19 (7,30) | 78.4 (48) | 195 |  |  |
|  |  |  |  | C | 269 (237,305) | 269 (237,305) | 96 (96,97) | 13 (8,21) | 79.3 (48) | 196 |  |  |
|  |  |  |  | D | 169 (158,183) | 169 (158,183) | -- | 57 (47,67) | 206.9 (49) | 321 |  |  |
| Gabon, 1985[11] | Pregnant F | 1737 (4) | HAI-1:40 | A | 17 (0,82) | 546 (24,1000) | 78 (76,100) | 173 (69,268) | 5.6 (1) | -- | B | C |
|  |  |  |  | B | 79 (66,91) | 21 (14,29) | -- | 113 (71,159) | 17.4 (2) | 56 |  |  |
|  |  |  |  | C | 87 (70,109) | 87 (70,109) | 83 (79,87) | 177 (134,215) | 14.3 (2) | 53 |  |  |
|  |  |  |  | D | 44 (42,47) | 44 (42,47) | -- | 261 (257,263) | 52.3 (3) | 79 |  |  |

| **Country, year of study** | **Study population** | **Sample size (no. of age groups)** | **Lab test (cut-off)** | **Cata-lytic model** | **Force of infection (/1000/year)** | | **Sensitivity (%)** | **CRS/ 100,000 live births** | **Loglike-lihood deviance (deg of freedom)** | **AICc** | **Selected model based on criterion:** | |
| --- | --- | --- | --- | --- | --- | --- | --- | --- | --- | --- | --- | --- |
| Ghana, 1997[12] | Pregnant F | 404 (3) | SRH | A | 120 (58,200) | 60 (10,589) | 100 (93,100) | 112 (14,215) | 0.9 (0) | -- | B | D |
|  |  |  |  | B | 120 (71,196) | 60 (10,100) | -- | 112 (14,215) | 0.9 (1) | -- |  |  |
|  |  |  |  | C | 116 (78,993) | 116 (78,993) | 96 (91,100) | 122 (0,196) | 1.6 (1) | -- |  |  |
|  |  |  |  | D | 85 (75,99) | 85 (75,99) | -- | 181 (152,204) | 2.3 (2) | 19 |  |  |
| Kenya (Kilifi), 1996-9[2-3] | Pregnant F | 276 (5) | EIA | A | 79 (0,126) | 91 (22,678) | 89 (75,100) | 195 (70,333) | 0.3 (2) | 50 | B | D |
|  |  |  |  | B | 73 (42,107) | 45 (11,87) | -- | 183 (50,310) | 0.5 (3) | 30 |  |  |
|  |  |  |  | C | 80 (56,170) | 80 (56,170) | 90 (76,100) | 192 (57,244) | 0.3 (3) | 30 |  |  |
|  |  |  |  | D | 61 (51,71) | 61 (51,71) | -- | 235 (214,252) | 1.1 (4) | 24 |  |  |
| Madagascar, 1990-1995[13] | Pregnant F | 567 (6) | EIA, ELFA, HAI | A | 0 (0,176) | 501 (4,857) | 80 (77,100) | 235 (6,357) | 4.3 (3) | 48 | B | B |
|  |  |  |  | B | 104 (73,135) | 17 (0,42) | -- | 71 (0,181) | 5.2 (4) | 39 |  |  |
|  |  |  |  | C | 131 (72,935) | 131 (72,935) | 82 (77,92) | 99 (0,210) | 5.2 (4) | 39 |  |  |
|  |  |  |  | D | 55 (50,62) | 55 (50,62) | -- | 245 (232,254) | 13.3 (5) | 42 |  |  |
| Mozambique, 2002[14] | Pregnant F | 962 (3) | EIA | A | 211 (0,282) | 164 (11,1000) | 97 (95,100) | 34 (7,106) | 0 (0) | -- | B | D |
|  |  |  |  | B | 209 (173,251) | 39 (1,88) | -- | 31 (1,61) | 0.4 (1) | -- |  |  |
|  |  |  |  | C | 202 (160,885) | 202 (160,885) | 97 (95,99) | 36 (0,66) | 0 (1) | -- |  |  |
|  |  |  |  | D | 136 (123,153) | 136 (123,153) | -- | 92 (73,110) | 18.4 (2) | 38 |  |  |
| Niger, <1991[98] | Pregnant F | 345 (3) | ? | A | 957 (8,994) | 852 (0,997) | 71 (67,96) | 0 (0,75) | 4.7 (0) | -- | D  dropped – poor fit | D  dropped – poor fit |
|  |  |  |  | B | 98 (87,113) | 0 (0,0) | -- | 0 (0,0) | 4.7 (1) | -- |  |  |
|  |  |  |  | C | 991 (293,999) | 991 (293,999) | 71 (66,76) | 0 (0,9) | 4.7 (1) | -- |  |  |
|  |  |  |  | D | 50 (43,57) | 50 (43,57) | -- | 254 (242,261) | 32.8 (2) | 53 |  |  |
| **Country, year of study** | **Study population** | **Sample size (no. of age groups)** | **Lab test (cut-off)** | **Cata-lytic model** | **Force of infection (/1000/year)** | | **Sensitivity (%)** | **CRS/ 100,000 live births** | **Loglike-lihood deviance (deg of freedom)** | **AICc** | **Selected model based on criterion:** | |
| Nigeria, <1978[15] | Sera from F in Obs. & Gynae. | 500 (5) | HAI-? | A | 0 (0,100) | 455 (17,828) | 75 (71,100) | 259 (55,349) | 2.6 (2) | 55 | B | C |
|  |  |  |  | B | 87 (62,107) | 18 (0,48) | -- | 90 (0,208) | 5.1 (3) | 38 |  |  |
|  |  |  |  | C | 124 (72,401) | 124 (72,401) | 77 (70,90) | 109 (2,210) | 4.3 (3) | 37 |  |  |
|  |  |  |  | D | 55 (49,62) | 55 (49,62) | -- | 246 (233,255) | 12.7 (4) | 39 |  |  |
| Nigeria, <2002[16] | Pregnant F | 207 (5) | EIA | A | 4 (0,23) | 78 (41,168) | 88 (68,100) | 496 (334,526) | 1.725 (2) | 52 | C | D |
|  |  |  |  | B | 7 (0,26) | 57 (37,74) | -- | 449 (305,518) | 1.872 (3) | 32 |  |  |
|  |  |  |  | C | 32 (26,38) | 32 (26,38) | 100 (100,100) | 260 (250,264) | 7.986 (3) | 38 |  |  |
|  |  |  |  | D | 32 (26,38) | 32 (26,38) | -- | 260 (250,264) | 7.986 (4) | 31 |  |  |
| Nigeria, 2007-8 [17] | Pregnant F | 404 (4) | EIA | A | 312 (0,994) | 0 (0,979) | 100 (96,100) | 0 (0,241) | 3.8 (1) | -- | D | D |
|  |  |  |  | B | 296 (249,351) | 0 (0,35) | -- | 0 (0,13) | 3.8 (2) | 31 |  |  |
|  |  |  |  | C | 806 (206,949) | 806 (206,949) | 98 (96,99) | 0 (0,34) | 3.8 (2) | 31 |  |  |
|  |  |  |  | D | 131 (113,162) | 131 (113,162) | -- | 98 (63,127) | 15.3 (3) | 30 |  |  |
| Senegal, 1996-2001[18] | F (child-bearing age) | 3471 (6) | EIA | A | 173 (0,859) | 9 (0,1000) | 100 (90,100) | 18 (0,124) | 2 (3) | 53 | B | B |
|  |  |  |  | B | 173 (151,189) | 9 (0,26) | -- | 18 (0,52) | 2 (4) | 43 |  |  |
|  |  |  |  | C | 259 (153,979) | 259 (153,979) | 90 (89,92) | 15 (0,72) | 3.1 (4) | 44 |  |  |
|  |  |  |  | D | 81 (78,85) | 81 (78,85) | -- | 190 (181,197) | 75 (5) | 111 |  |  |
| South Africa, 2003[19] | Pregnant F | 1200 (3) | EIA | A | 213 (0,275) | 149 (16,989) | 98 (97,100) | 34 (9,153) | 0 (0) | -- | B | D |
|  |  |  |  | B | 222 (177,271) | 48 (4,106) | -- | 29 (3,57) | 0.3 (1) | -- |  |  |
|  |  |  |  | C | 197 (159,337) | 197 (159,337) | 98 (96,99) | 38 (5,67) | 0 (1) | -- |  |  |
|  |  |  |  | D | 146 (132,165) | 146 (132,165) | -- | 80 (61,98) | 13.9 (2) | 32 |  |  |
| **Country, year of study** | **Study population** | **Sample size (no. of age groups)** | **Lab test (cut-off)** | **Cata-lytic model** | **Force of infection (/1000/year)** | | **Sensitivity (%)** | **CRS/ 100,000 live births** | **Loglike-lihood deviance (deg of freedom)** | **AICc** | **Selected model based on criterion:** | |
| Zambia, 1979-80[20] | F post-partum | 100 (6) | RH & HAI-1:10 | A | 97 (26,178) | 84 (20,266) | 100 (96,100) | 157 (35,317) | 3.2 (5) | 32 | A | D |
|  |  |  |  | B | 97 (32,178) | 84 (19,192) | -- | 157 (33,317) | 3.2 (5) | 22 |  |  |
|  |  |  |  | C | 91 (72,996) | 91 (72,996) | 100 (87,100) | 169 (0,210) | 3.2 (5) | 22 |  |  |
|  |  |  |  | D | 91 (72,121) | 91 (72,121) | -- | 169 (113,211) | 3.2 (5) | 17 |  |  |
| **Eastern Mediterranean** | | | | | | | | | | | | |
| Pakistan, <1997 [37] | General pop. (M/F?) | 300 (6) | EIA | A | 178 (103,338) | 136 (25,1000) | 84 (74,100) | 54 (2,141) | 6.3 (3) | 49 | B | C |
|  |  |  |  | B | 118 (95,144) | 27 (0,74) | -- | 81 (0,151) | 8.1 (4) | 41 |  |  |
|  |  |  |  | C | 182 (110,344) | 182 (110,344) | 83 (74,94) | 48 (4,132) | 6.3 (4) | 39 |  |  |
|  |  |  |  | D | 93 (79,110) | 93 (79,110) | -- | 165 (131,194) | 16.1 (5) | 43 |  |  |
| Pakistan, 1999-2004[38] | Pregnant F | 1163 (5) | EIA | A | 144 (0,952) | 8 (0,1000) | 100 (84,100) | 24 (0,128) | 16.1 (2) | 70 | B | B |
|  |  |  |  | B | 144 (122,161) | 8 (0,28) | -- | 24 (0,77) | 16.1 (3) | 50 |  |  |
|  |  |  |  | C | 559 (137,995) | 559 (137,995) | 85 (83,89) | 0 (0,91) | 16.6 (3) | 51 |  |  |
|  |  |  |  | D | 74 (68,79) | 74 (68,79) | -- | 207 (194,218) | 45.8 (4) | 73 |  |  |
| Yemen, 1985[42] | Residual sera? | 476 (6) | HAI-? | A | 255 (179,364) | 169 (104,1000) | 85 (80,91) | 20 (2,52) | 13.3 (3) | 57 | C | C |
|  |  |  |  | B | 163 (139,185) | 0 (0,40) | -- | 0 (0,68) | 20.4 (4) | 54 |  |  |
|  |  |  |  | C | 258 (183,365) | 258 (183,365) | 85 (80,90) | 16 (3,47) | 13.3 (4) | 47 |  |  |
|  |  |  |  | D | 116 (100,134) | 116 (100,134) | -- | 122 (95,151) | 45.4 (5) | 74 |  |  |

| **Country, year of study** | **Study population** | **Sample size (no. of age groups)** | **Lab test (cut-off)** | **Cata-lytic model** | **Force of infection (/1000/year)** | | **Sensitivity (%)** | **CRS/ 100,000 live births** | **Loglike-lihood deviance (deg of freedom)** | **AICc** | **Selected model based on criterion:** | |
| --- | --- | --- | --- | --- | --- | --- | --- | --- | --- | --- | --- | --- |
| Yemen, 2002-03[43] | Unvaccinated pop. (M/F?) | 1253 (5) | EIA | A | 192 (174,216) | 22 (9,1000) | 100 (95,100) | 28 (6,45) | 11.1 (2) | 66 | B | B |
|  |  |  |  | B | 192 (174,212) | 22 (9,42) | -- | 28 (12,47) | 11.1 (3) | 46 |  |  |
|  |  |  |  | C | 206 (182,235) | 206 (182,235) | 96 (95,98) | 33 (22,48) | 12.8 (3) | 47 |  |  |
|  |  |  |  | D | 149 (135,166) | 149 (135,166) | -- | 76 (60,93) | 71.5 (4) | 99 |  |  |
| **South East Asia** | | | | | | | | | | | | |
| Bangladesh, 2004-05[53] | F aged 1-45 yrs | 582 (9) | HAI? – 1:10 | A | 110 (84,143) | 59 (22,1000) | 93 (82,100) | 126 (18,168) | 1.7 (6) | 50 | B | B |
|  |  |  |  | B | 99 (82,120) | 35 (16,54) | -- | 118 (59,169) | 1.8 (7) | 45 |  |  |
|  |  |  |  | C | 117 (88,157) | 117 (88,157) | 88 (83,94) | 121 (69,174) | 2 (7) | 45 |  |  |
|  |  |  |  | D | 70 (62,79) | 70 (62,79) | -- | 215 (194,232) | 16.6 (8) | 56 |  |  |
| India (urban Delhi), 1968[54] | 15-34+ year old F | 217 (5) | HAI | A | 160 (103,325) | 64 (0,1000) | 91 (80,100) | 69 (0,137) | 0.2 (2) | 49 | B | B |
|  |  |  |  | B | 129 (99,158) | 27 (0,85) | -- | 70 (0,140) | 0.4 (3) | 29 |  |  |
|  |  |  |  | C | 178 (108,349) | 178 (108,349) | 87 (79,98) | 50 (4,136) | 0.4 (3) | 29 |  |  |
|  |  |  |  | D | 97 (80,118) | 97 (80,118) | -- | 157 (118,193) | 7.5 (4) | 30 |  |  |
| India (rural Delhi), 1968[54] | 15-34+ year old F | 204 (5) | HAI | A | 85 (66,177) | 19 (0,1000) | 100 (68,100) | 96 (0,204) | 0.7 (2) | 51 | B | D |
|  |  |  |  | B | 85 (63,109) | 19 (0,60) | -- | 96 (0,218) | 0.7 (3) | 31 |  |  |
|  |  |  |  | C | 127 (68,217) | 127 (68,217) | 77 (67,97) | 105 (29,218) | 1.2 (3) | 31 |  |  |
|  |  |  |  | D | 64 (53,77) | 64 (53,77) | -- | 228 (199,249) | 6.3 (4) | 30 |  |  |

| **Country, year of study** | **Study population** | **Sample size (no. of age groups)** | **Lab test (cut-off)** | **Cata-lytic model** | **Force of infection (/1000/year)** | | **Sensitivity (%)** | **CRS/ 100,000 live births** | **Loglike-lihood deviance (deg of freedom)** | **AICc** | **Selected model based on criterion:** | |
| --- | --- | --- | --- | --- | --- | --- | --- | --- | --- | --- | --- | --- |
| India (Chandrigarh), 1972-3[54] | 15-34+ year old F | 365 (6) | HAI | A | 134 (115,518) | 0 (0,1000) | 100 (78,100) | 0 (0,47) | 3.3 (3) | 44 | C | C |
|  |  |  |  | B | 133 (114,151) | 0 (0,17) | -- | 0 (0,56) | 3.3 (4) | 34 |  |  |
|  |  |  |  | C | 244 (159,543) | 244 (159,543) | 81 (77,86) | 19 (0,66) | 4.5 (4) | 35 |  |  |
|  |  |  |  | D | 74 (65,85) | 74 (65,85) | -- | 205 (182,227) | 29.6 (5) | 55 |  |  |
| India (Lucknow), 1972-3[54] | 15-34+ year old F | 412 (6) | HAI | A | 133 (91,206) | 201 (69,635) | 89 (85,96) | 85 (25,145) | 2.6 (3) | 45 | B | C |
|  |  |  |  | B | 123 (97,154) | 32 (5,63) | -- | 83 (13,148) | 5.6 (4) | 38 |  |  |
|  |  |  |  | C | 140 (105,211) | 140 (105,211) | 90 (85,95) | 87 (31,141) | 2.9 (4) | 35 |  |  |
|  |  |  |  | D | 82 (71,94) | 82 (71,94) | -- | 189 (162,212) | 19 (5) | 46 |  |  |
| India (Calcutta), 1976[55] | Patients at skin clinics, mothers & babies | 344 (7) | ? | A | 226 (111,1000) | 460 (79,1000) | 65 (56,76) | 15 (0,111) | 17 (4) | 60 | B | B |
|  |  |  |  | B | 82 (66,98) | 8 (0,26) | -- | 49 (0,143) | 28.6 (5) | 65 |  |  |
|  |  |  |  | C | 231 (117,1000) | 231 (117,1000) | 65 (56,75) | 23 (0,120) | 17 (5) | 53 |  |  |
|  |  |  |  | D | 50 (43,59) | 50 (43,59) | -- | 254 (238,262) | 51.7 (6) | 84 |  |  |
| India (Delhi), <1987[56] | Pregnant F | 160 (5) | ? | A | 54 (18,1000) | 9 (0,1000) | 100 (47,100) | 77 (0,320) | 5.9 (2) | 54 | B | D |
|  |  |  |  | B | 54 (27,73) | 9 (0,41) | -- | 77 (0,306) | 5.9 (3) | 34 |  |  |
|  |  |  |  | C | 255 (29,1000) | 255 (29,1000) | 54 (46,100) | 17 (0,264) | 6.2 (3) | 34 |  |  |
|  |  |  |  | D | 33 (26,41) | 33 (26,41) | -- | 262 (250,264) | 8.1 (4) | 30 |  |  |

| **Country, year of study** | **Study population** | **Sample size (no. of age groups)** | **Lab test (cut-off)** | **Cata-lytic model** | **Force of infection (/1000/year)** | | **Sensitivity (%)** | **CRS/ 100,000 live births** | **Loglike-lihood deviance (deg of freedom)** | **AICc** | **Selected model based on criterion:** | |
| --- | --- | --- | --- | --- | --- | --- | --- | --- | --- | --- | --- | --- |
| India (Delhi), <1990[57] | Random selection of F | 99 (3) | RH & HAI | A | 34 (0,137) | 71 (0,740) | 100 (58,100) | 338 (0,508) | 0.1 (0) | -- | D | D |
|  |  |  |  | B | 34 (0,79) | 71 (0,145) | -- | 338 (0,512) | 0.1 (1) | -- |  |  |
|  |  |  |  | C | 47 (36,992) | 47 (36,992) | 100 (57,100) | 258 (0,264) | 0.4 (1) | -- |  |  |
|  |  |  |  | D | 47 (36,61) | 47 (36,61) | -- | 258 (235,264) | 0.4 (2) | 18 |  |  |
| India (rural Vellore), 1999-2000[1] | rural F | 1693 (39) | EIA (serum & saliva): <4IU/ml for serum | A | 116 (102,127) | 224 (60,553) | 93 (91,100) | 100 (49,138) | 87.8 (36) | 220 | B | C |
|  |  |  |  | B | 112 (102,123) | 72 (55,92) | -- | 127 (107,147) | 90.9 (37) | 221 |  |  |
|  |  |  |  | C | 117 (105,130) | 117 (105,130) | 96 (94,99) | 120 (100,141) | 89.6 (37) | 219 |  |  |
|  |  |  |  | D | 99 (93,106) | 99 (93,106) | -- | 153 (140,165) | 99.2 (38) | 227 |  |  |
| India (urban Vellore), 1999-2000 [1] | urban F | 1409 (39) | EIA (serum & saliva): <4IU/ml for serum | A | 175 (154,197) | 206 (60,1000) | 97 (95,100) | 50 (8,69) | 51.3 (36) | 155 | B | C |
|  |  |  |  | B | 170 (153,190) | 67 (39,104) | -- | 61 (42,77) | 54.8 (37) | 156 |  |  |
|  |  |  |  | C | 176 (156,200) | 176 (156,200) | 97 (96,99) | 52 (37,70) | 51.4 (37) | 153 |  |  |
|  |  |  |  | D | 140 (129,154) | 140 (129,154) | -- | 87 (71,102) | 77.3 (38) | 177 |  |  |

| **Country, year of study** | **Study population** | **Sample size (no. of age groups)** | **Lab test (cut-off)** | **Cata-lytic model** | **Force of infection (/1000/year)** | | **Sensitivity (%)** | **CRS/ 100,000 live births** | **Loglike-lihood deviance (deg of freedom)** | **AICc** | **Selected model based on criterion:** | |
| --- | --- | --- | --- | --- | --- | --- | --- | --- | --- | --- | --- | --- |
| Nepal, 2008[58] | outpatient F aged 15 -39 yrs | 2224 (5) | EIA: <10IU | A | 0 (0,308) | 988 (0,1000) | 91 (90,100) | 82 (0,139) | 4.8 (2) | 62 | B | B |
|  |  |  |  | B | 180 (157,199) | 9 (0,31) | -- | 17 (0,52) | 7.6 (3) | 45 |  |  |
|  |  |  |  | C | 225 (168,939) | 225 (168,939) | 91 (90,93) | 25 (0,58) | 6.3 (3) | 44 |  |  |
|  |  |  |  | D | 94 (89,100) | 94 (89,100) | -- | 163 (151,174) | 82.4 (4) | 113 |  |  |
| **Western Pacific** | | | | | | | | | | | | |
| Central Vietnam, 2009-2010[65] | pregnant F | 1988 (23) | EIA: <4 IU/ml | A | 51 (0,67) | 40 (28,215) | 100 (75,100) | 227 (158,479) | 29.7 (20) | 141 | A | D |
|  |  |  |  | B | 52 (36,69) | 40 (25,53) | -- | 227 (146,308) | 29.7 (21) | 138 |  |  |
|  |  |  |  | C | 52 (43,77) | 52 (43,77) | 93 (81,100) | 250 (199,261) | 29.8 (21) | 138 |  |  |
|  |  |  |  | D | 45 (43,48) | 45 (43,48) | -- | 260 (257,262) | 30.4 (22) | 136 |  |  |

**Notes:**

**Types of catalytic model:** A- full model (force of infection among younger and older individuals and test sensitivity are estimated; B-similar to version used in previous analyses[99] (force of infection among younger and older individuals estimated, sensitivity=100%); C-force of infection is assumed to be age-independent, and is estimated, as is the test sensitivity; D- force of infection is assumed to be age-independent, and is estimated, test sensitivity=100%

**Population:**

F-females, M-males;

**Type of assay used**

LA: latex agglutination; EIA: enzyme-based immunoassay; MEIA: micro enzyme immunoassay; HAI: hemagglutination inhibition; RH: Radial haemolysis

Table : Summary of the studies, best-fitting values for the force of infection predating the introduction of vaccination and (where appropriate) the sensitivity of the antibody assay, and the CRS incidence per 100,000 live births for each catalytic model, for settings in which rubella vaccine had not been introduced by 2010. The values in parentheses reflect the 95% confidence intervals, obtained by bootstrapping. To facilitate comparisons, the infection and CRS incidence is not weighted by the number of live births.

| **Country, year of study, reference** | **Study population** | **Sample size (no. of age groups)** | **Lab test (cut-off)** | **Cata-lytic model** | **Force of infection (/1000/year)** | | **Sensitivity (%)** | **CRS/ 100,000 live births** | **Loglike-lihood deviance (deg of freedom)** | **AICc** | **Selected model according to criterion:** | |
| --- | --- | --- | --- | --- | --- | --- | --- | --- | --- | --- | --- | --- |
|  |  |  |  |  | **<13 yr olds** | **≥13 yr olds** |  |  |  |  | **1** | **2** |
| **America** | | | | | | | | | | | | |
| Argentina (urban), 1967-68[21] | Sera from urban & rural M&F | 491 (5) | HAI-1:10 | A | 213 (123,415) | 40 (0,395) | 86 (80,100) | 31 (0,90) | 2 (2) | 55 | A | C |
|  |  |  |  | B | 137 (113,154) | 5 (0,35) | -- | 16 (0,97) | 3.2 (3) | 36 |  |  |
|  |  |  |  | C | 246 (154,442) | 246 (154,442) | 83 (79,88) | 19 (1,71) | 2.7 (3) | 35 |  |  |
|  |  |  |  | D | 90 (79,102) | 90 (79,102) | -- | 172 (147,194) | 27.5 (4) | 53 |  |  |
| Argentina (rural), 1967-68[21] | Sera from urban & rural M&F | 129 (5) | HAI-1:10 | A | 156 (92,1000) | 54 (0,131) | 94 (81,100) | 69 (0,163) | 5.1 (2) | 50 | B | D |
|  |  |  |  | B | 130 (92,178) | 36 (0,103) | -- | 81 (0,163) | 5.2 (3) | 30 |  |  |
|  |  |  |  | C | 190 (91,1000) | 190 (91,1000) | 88 (79,100) | 42 (0,169) | 5.8 (3) | 31 |  |  |
|  |  |  |  | D | 97 (78,127) | 97 (78,127) | -- | 156 (104,196) | 8 (4) | 26 |  |  |
| Argentina (Mar de Plata), 1981[22] | Gen popn F going to health checks | 769 (5) | HAI-1:8 | A | 695 (44,977) | 0 (0,1000) | 88 (87,100) | 0 (0,67) | 1 (2) | 52 | D | D |
|  |  |  |  | B | 172 (147,186) | 0 (0,28) | -- | 0 (0,56) | 1 (3) | 32 |  |  |
|  |  |  |  | C | 970 (199,999) | 970 (199,999) | 88 (86,91) | 0 (0,37) | 1 (3) | 32 |  |  |
|  |  |  |  | D | 99 (91,110) | 99 (91,110) | -- | 152 (133,170) | 28.3 (4) | 52 |  |  |
| Brazil, 1967-68[21] | Sera from urban & rural M&F | 295 (6) | HAI-1:10 | A | 163 (132,229) | 12 (0,1000) | 100 (90,100) | 26 (0,81) | 4.6 (3) | 44 | B | B |
|  |  |  |  | B | 163 (133,193) | 12 (0,51) | -- | 26 (0,83) | 4.6 (4) | 34 |  |  |
|  |  |  |  | C | 208 (145,301) | 208 (145,301) | 90 (86,95) | 33 (8,81) | 6.9 (4) | 36 |  |  |
|  |  |  |  | D | 103 (88,124) | 103 (88,124) | -- | 144 (109,175) | 26.1 (5) | 50 |  |  |

| **Country, year of study, reference** | **Study population** | **Sample size (no. of age groups)** | **Lab test (cut-off)** | **Cata-lytic model** | **Force of infection (/1000/year)** | | **Sensitivity (%)** | **CRS/ 100,000 live births** | **Loglike-lihood deviance (deg of freedom)** | **AICc** | **Selected model according to criterion:** | |
| --- | --- | --- | --- | --- | --- | --- | --- | --- | --- | --- | --- | --- |
|  |  |  |  |  | **<13 yr olds** | **≥13 yr olds** |  |  |  |  | **1** | **2** |
| Brazil, 1987[23] | Residual sera (children), & cord blood (adults) | 1729 (11) | EIA | A | 131 (117,154) | 88 (50,278) | 97 (91,100) | 102 (62,120) | 16.8 (8) | 80 | A | B |
|  |  |  |  | B | 126 (116,136) | 68 (43,100) | -- | 107 (86,122) | 16.9 (9) | 77 |  |  |
|  |  |  |  | C | 137 (121,157) | 137 (121,157) | 95 (91,99) | 91 (69,114) | 17.2 (9) | 77 |  |  |
|  |  |  |  | D | 118 (110,127) | 118 (110,127) | -- | 118 (105,132) | 25.7 (10) | 82 |  |  |
| Brazil (Parana), 1996-8[24] | Pregnant F | 1348 (3) | EIA | A | 203 (139,996) | 1000 (0,1000) | 90 (88,99) | 6 (0,50) | 0.2 (0) | - | B | D |
|  |  |  |  | B | 172 (149,189) | 6 (0,26) | -- | 13 (0,51) | 1.3 (1) | - |  |  |
|  |  |  |  | C | 238 (177,999) | 238 (177,999) | 90 (88,92) | 21 (0,52) | 0.4 (1) | - |  |  |
|  |  |  |  | D | 89 (83,97) | 89 (83,97) | -- | 172 (157,185) | 70.5 (2) | 93 |  |  |
| Canada, <1967 [25] | Residual sera | 198 (5) | HAI-? | A | 111 (72,154) | 179 (47,1000) | 93 (85,100) | 116 (13,201) | 0.3 (2) | 48 | B | D |
|  |  |  |  | B | 100 (71,135) | 76 (30,145) | -- | 148 (77,208) | 0.6 (3) | 28 |  |  |
|  |  |  |  | C | 108 (81,162) | 108 (81,162) | 96 (87,100) | 136 (64,191) | 0.5 (3) | 28 |  |  |
|  |  |  |  | D | 93 (78,114) | 93 (78,114) | -- | 165 (125,196) | 1.1 (4) | 22 |  |  |
| Chile (Santiago), 1967-68[21] | Sera from urban M&F | 281 (5) | HAI-1:10 | A | 1000 (389,1000) | 104 (24,163) | 97 (95,99) | 0 (0,4) | 12.8 (2) | 51 | B | B |
|  |  |  |  | B | 303 (240,408) | 27 (0,86) | -- | 8 (0,25) | 19.7 (3) | 38 |  |  |
|  |  |  |  | C | 1000 (448,1000) | 1000 (448,1000) | 97 (95,99) | 0 (0,1) | 12.8 (3) | 31 |  |  |
|  |  |  |  | D | 226 (183,312) | 226 (183,312) | -- | 25 (7,47) | 26.8 (4) | 38 |  |  |

| **Country, year of study, reference** | **Study population** | **Sample size (no. of age groups)** | **Lab test (cut-off)** | **Cata-lytic model** | **Force of infection (/1000/year)** | | **Sensitivity (%)** | **CRS/ 100,000 live births** | **Loglike-lihood deviance (deg of freedom)** | **AICc** | **Selected model according to criterion:** | |
| --- | --- | --- | --- | --- | --- | --- | --- | --- | --- | --- | --- | --- |
|  |  |  |  |  | **<13 yr olds** | **≥13 yr olds** |  |  |  |  | **1** | **2** |
| Chile (rural), 1967-68[21] | Sera from rural M&F | 163 (5) | HAI-1:10 | A | 370 (271,584) | 1000 (1000,1000) | 100 (100,100) | 1 (0,3) | 1.1 (2) | 34 | C | D |
|  |  |  |  | B | 370 (271,584) | 1000 (1000,1000) | -- | 1 (0,3) | 1.1 (3) | 14 |  |  |
|  |  |  |  | C | 381 (293,586) | 381 (293,586) | 100 (100,100) | 3 (0,9) | 1.3 (3) | 14 |  |  |
|  |  |  |  | D | 381 (293,586) | 381 (293,586) | -- | 3 (0,9) | 1.3 (4) | 8 |  |  |
| Haiti, 2002[29] | Pregnant F (urban) | 425 (6) | EIA | A | 42 (0,883) | 1000 (0,1000) | 96 (95,100) | 48 (0,153) | 1.4 (3) | 34 | B | C |
|  |  |  |  | B | 249 (173,292) | 8 (0,90) | -- | 6 (0,58) | 1.7 (4) | 25 |  |  |
|  |  |  |  | C | 266 (157,999) | 266 (157,999) | 96 (94,99) | 14 (0,68) | 1.5 (4) | 24 |  |  |
|  |  |  |  | D | 128 (109,157) | 128 (109,157) | -- | 104 (69,133) | 13.1 (5) | 31 |  |  |
| Jamaica (Kingston), 1967-68[21] | Sera from urban M&F | 200 (5) | HAI-1:10 | A | 1000 (267,1000) | 7 (0,66) | 63 (57,70) | 0 (0,3) | 6.7 (2) | 57 | D | D |
|  |  |  |  | B | 88 (74,105) | 0 (0,0) | -- | 0 (0,0) | 19.1 (3) | 49 |  |  |
|  |  |  |  | C | 1000 (335,1000) | 1000 (335,1000) | 63 (57,69) | 0 (0,5) | 6.7 (3) | 37 |  |  |
|  |  |  |  | D | 58 (49,70) | 58 (49,70) | -- | 240 (216,255) | 47.1 (4) | 71 |  |  |
| Jamaica (rural), 1967-68[21] | Sera from rural M&F | 200 (5) | HAI-1:10 | A | 169 (49,1000) | 219 (45,958) | 49 (38,75) | 52 (0,255) | 6.1 (2) | 57 | B | D |
|  |  |  |  | B | 46 (32,59) | 11 (0,36) | -- | 102 (0,266) | 8.7 (3) | 39 |  |  |
|  |  |  |  | C | 170 (42,1000) | 170 (42,1000) | 49 (38,86) | 57 (0,260) | 6.2 (3) | 37 |  |  |
|  |  |  |  | D | 35 (28,42) | 35 (28,42) | -- | 263 (254,264) | 12.8 (4) | 37 |  |  |

| **Country, year of study, reference** | **Study population** | **Sample size (no. of age groups)** | **Lab test (cut-off)** | **Cata-lytic model** | **Force of infection (/1000/year)** | | **Sensitivity (%)** | **CRS/ 100,000 live births** | **Loglike-lihood deviance (deg of freedom)** | **AICc** | **Selected model according to criterion:** | |
| --- | --- | --- | --- | --- | --- | --- | --- | --- | --- | --- | --- | --- |
|  |  |  |  |  | **<13 yr olds** | **≥13 yr olds** |  |  |  |  | **1** | **2** |
| Mexico, 1987-88[26] | Sera from blood collection | 24331 (7) | HAI-1:8 | A | 133 (125,139) | 101 (72,134) | 89 (87,91) | 100 (90,109) | 1 (4) | -40 | A | C |
|  |  |  |  | B | 107 (104,110) | 32 (29,35) | -- | 102 (94,110) | 23 (5) | -25 |  |  |
|  |  |  |  | C | 134 (128,141) | 134 (128,141) | 87 (87,88) | 95 (86,104) | 5 (5) | -43 |  |  |
|  |  |  |  | D | 76 (75,78) | 76 (75,78) | -- | 201 (198,204) | 799.8 (6) | 747 |  |  |
| Mexico, 1989[27] | F of reproductive age | 428 (6) | HAI:1:8 | A | 31 (17,48) | 51 (35,85) | 100 (86,100) | 326 (233,416) | 2.1 (3) | 46 | C | D |
|  |  |  |  | B | 31 (17,47) | 51 (34,69) | -- | 326 (231,412) | 2.1 (4) | 36 |  |  |
|  |  |  |  | C | 40 (36,48) | 40 (36,48) | 100 (92,100) | 263 (257,264) | 3.8 (4) | 38 |  |  |
|  |  |  |  | D | 40 (36,46) | 40 (36,46) | -- | 263 (259,264) | 3.8 (5) | 33 |  |  |
| Panama (Panama City), 1967-68[21] | Sera from urban M&F | 240 (6) | HAI-1:10 | A | 67 (49,144) | 18 (2,1000) | 100 (65,100) | 56 (46,84) | 2.7 (3) | 45 | B | B |
|  |  |  |  | B | 67 (49,86) | 18 (0,39) | -- | 56 (46,66) | 2.7 (4) | 35 |  |  |
|  |  |  |  | C | 104 (48,206) | 104 (48,206) | 72 (62,98) | 73 (45,92) | 4.3 (4) | 36 |  |  |
|  |  |  |  | D | 45 (38,54) | 45 (38,54) | -- | 43 (38,49) | 10.2 (5) | 37 |  |  |
| Panama (rural), 1967-68[21] | Sera from rural M&F | 268 (6) | HAI-1:10 | A | 21 (13,36) | 25 (14,153) | 100 (54,100) | 23 (15,36) | 0 (3) | 48 | C | D |
|  |  |  |  | B | 21 (13,30) | 25 (14,37) | -- | 23 (15,32) | 5.9 (4) | 38 |  |  |
|  |  |  |  | C | 22 (18,48) | 22 (18,48) | 100 (61,100) | 24 (20,45) | 6.1 (4) | 38 |  |  |
|  |  |  |  | D | 22 (18,27) | 22 (18,27) | -- | 24 (20,29) | 6.1 (5) | 33 |  |  |

| **Country, year of study, reference** | **Study population** | **Sample size (no. of age groups)** | **Lab test (cut-off)** | **Cata-lytic model** | **Force of infection (/1000/year)** | | **Sensitivity (%)** | **CRS/ 100,000 live births** | **Loglike-lihood deviance (deg of freedom)** | **AICc** | **Selected model according to criterion:** | |
| --- | --- | --- | --- | --- | --- | --- | --- | --- | --- | --- | --- | --- |
|  |  |  |  |  | **<13 yr olds** | **≥13 yr olds** |  |  |  |  | **1** | **2** |
| Peru (Lima), 1967-68[21] | Sera from urban M&F | 243 (5) | HAI-1:10 | A | 302 (149,1000) | 0 (0,4) | 83 (76,100) | 0 (0,0) | 15.4 (2) | 64 | D | C |
|  |  |  |  | B | 143 (122,169) | 0 (0,0) | -- | 0 (0,0) | 18.6 (3) | 47 |  |  |
|  |  |  |  | C | 385 (265,1000) | 385 (265,1000) | 81 (75,86) | 2 (0,14) | 16.2 (3) | 45 |  |  |
|  |  |  |  | D | 93 (79,113) | 93 (79,113) | -- | 165 (128,195) | 60.9 (4) | 83 |  |  |
| Peru (rural), 1967-68[21] | Sera from M&F | 203 (5) | EIA | A | 31 (19,48) | 62 (38,138) | 100 (75,100) | 342 (242,413) | 2.2 (2) | 51 | C | D |
|  |  |  |  | B | 31 (19,44) | 62 (36,89) | -- | 342 (245,413) | 2.2 (3) | 31 |  |  |
|  |  |  |  | C | 41 (33,50) | 41 (33,50) | 100 (99,100) | 263 (253,264) | 4.7 (3) | 34 |  |  |
|  |  |  |  | D | 41 (33,49) | 41 (33,49) | -- | 263 (255,264) | 4.7 (4) | 27 |  |  |
| Peru, 2003[28] | Postpartum F | 1236 (7) | HAI-1:10 | A | 99 (0,155) | 325 (19,877) | 89 (87,100) | 100 (44,208) | 6.4 (4) | 46 | B | C |
|  |  |  |  | B | 132 (110,156) | 33 (11,56) | -- | 76 (27,121) | 6.8 (5) | 39 |  |  |
|  |  |  |  | C | 145 (113,217) | 145 (113,217) | 91 (88,94) | 81 (28,127) | 6.6 (5) | 39 |  |  |
|  |  |  |  | D | 86 (80,92) | 86 (80,92) | -- | 180 (166,193) | 25.6 (6) | 54 |  |  |
| Trinidad, 1966-7[30] | Sera from M & F | 71 (2) | ? | A | 0 (0,5) | 36 (24,78) | 100 (68,100) | 416 (332,501) | 1 (-1) | -- | C | C |
|  |  |  |  | B | 0 (0,11) | 36 (21,53) | -- | 416 (289,482) | 1 (0) | -- |  |  |
|  |  |  |  | C | 19 (13,27) | 19 (13,27) | 100 (100,100) | 221 (179,252) | 4.8 (0) | -- |  |  |
|  |  |  |  | D | 19 (13,27) | 19 (13,27) | -- | 221 (179,252) | 4.8 (1) | -- |  |  |
| **Country, year of study, reference** | **Study population** | **Sample size (no. of age groups)** | **Lab test (cut-off)** | **Cata-lytic model** | **Force of infection (/1000/year)** | | **Sensitivity (%)** | **CRS/ 100,000 live births** | **Loglike-lihood deviance (deg of freedom)** | **AICc** | **Selected model according to criterion:** | |
|  |  |  |  |  | **<13 yr olds** | **≥13 yr olds** |  |  |  |  | **1** | **2** |
| Trinidad (Port au Spain), 1967-68[21] | Sera from urban M&F | 200 (5) | HAI-1:10 | A | 57 (17,305) | 33 (0,1000) | 54 (29,100) | 193 (0,315) | 0.3 (2) | 50 | B | D |
|  |  |  |  | B | 27 (15,37) | 10 (0,29) | -- | 119 (0,290) | 0.4 (3) | 30 |  |  |
|  |  |  |  | C | 78 (18,341) | 78 (18,341) | 43 (28,100) | 198 (5,264) | 0.4 (3) | 30 |  |  |
|  |  |  |  | D | 21 (16,27) | 21 (16,27) | -- | 232 (201,251) | 2.2 (4) | 25 |  |  |
| Trinidad (rural), 1967-8[21] | Sera from rural M&F | 200 (5) | HAI-1:10 | A | 22 (12,160) | 9 (0,1000) | 100 (28,100) | 121 (0,285) | 3.6 (2) | 52 | B | D |
|  |  |  |  | B | 22 (12,30) | 9 (0,26) | -- | 121 (0,284) | 3.6 (3) | 32 |  |  |
|  |  |  |  | C | 52 (14,240) | 52 (14,240) | 45 (25,100) | 250 (20,264) | 4.2 (3) | 33 |  |  |
|  |  |  |  | D | 17 (12,23) | 17 (12,23) | -- | 212 (174,238) | 4.8 (4) | 27 |  |  |
| Uruguay (urban) 1967-68[21] | Sera from urban M&F | 270 (6) | HAI-1:10 | A | 297 (156,999) | 54 (0,105) | 87 (81,100) | 12 (0,58) | 10.1 (3) | 49 | D | C |
|  |  |  |  | B | 162 (133,187) | 0 (0,34) | -- | 0 (0,69) | 13 (4) | 42 |  |  |
|  |  |  |  | C | 329 (190,999) | 329 (190,999) | 85 (80,91) | 6 (0,42) | 10.6 (4) | 40 |  |  |
|  |  |  |  | D | 100 (84,120) | 100 (84,120) | -- | 151 (115,184) | 42.8 (5) | 67 |  |  |
| Uruguay (rural) 1967-68[21] | Sera from rural M&F | 155 (4) | HAI-1:10 | A | 0 (0,965) | 781 (0,1000) | 88 (83,100) | 129 (0,271) | 0.4 (1) | - | B | D |
|  |  |  |  | B | 155 (97,192) | 4 (0,58) | -- | 10 (0,144) | 1.1 (2) | 31 |  |  |
|  |  |  |  | C | 200 (102,997) | 200 (102,997) | 88 (83,96) | 37 (0,147) | 0.8 (2) | 30 |  |  |
|  |  |  |  | D | 77 (64,98) | 77 (64,98) | -- | 200 (155,229) | 9.6 (3) | 27 |  |  |
| USA (Atlanta), <1967[25] | Residual sera | 172 (5) | HAI-? | A | 313 (147,1000) | 69 (0,1000) | 86 (79,100) | 10 (0,60) | 0.8 (2) | 48 | D | C |
|  |  |  |  | B | 161 (127,195) | 0 (0,36) | -- | 0 (0,70) | 2.1 (3) | 29 |  |  |
|  |  |  |  | C | 327 (175,1000) | 327 (175,1000) | 85 (79,92) | 6 (0,53) | 0.8 (3) | 28 |  |  |
|  |  |  |  | D | 98 (80,124) | 98 (80,124) | -- | 154 (109,193) | 19.7 (4) | 40 |  |  |
| **Country, year of study, reference** | **Study population** | **Sample size (no. of age groups)** | **Lab test (cut-off)** | **Cata-lytic model** | **Force of infection (/1000/year)** | | **Sensitivity (%)** | **CRS/ 100,000 live births** | **Loglike-lihood deviance (deg of freedom)** | **AICc** | **Selected model according to criterion:** | |
|  |  |  |  |  | **<13 yr olds** | **≥13 yr olds** |  |  |  |  | **1** | **2** |
| USA (Houston), <1967[25] | Residual sera | 173 (5) | HAI-? | A | 217 (109,1000) | 1000 (0,1000) | 82 (75,100) | 5 (0,115) | 1.5 (2) | 49 | B | C |
|  |  |  |  | B | 134 (97,163) | 4 (0,62) | -- | 14 (0,140) | 2.5 (3) | 30 |  |  |
|  |  |  |  | C | 220 (117,1000) | 220 (117,1000) | 82 (75,93) | 28 (0,120) | 1.7 (3) | 29 |  |  |
|  |  |  |  | D | 81 (67,101) | 81 (67,101) | -- | 190 (149,222) | 11.5 (4) | 32 |  |  |
| **Eastern Mediterranean** | | | | | | | | | | | | |
| Bahrain, 1981[31] | School children and pregnant F | 6097 (8) | HAI-1:8 | A | 107 (100,114) | 857 (522,1000) | 88 (87,89) | 29 (20,60) | 59.4 (5) | 124 | B | C |
|  |  |  |  | B | 102 (97,107) | 57 (47,68) | -- | 138 (123,150) | 160.6 (6) | 219 |  |  |
|  |  |  |  | C | 114 (107,121) | 114 (107,121) | 93 (92,95) | 126 (114,138) | 148.4 (6) | 207 |  |  |
|  |  |  |  | D | 91 (88,95) | 91 (88,95) | -- | 168 (161,175) | 202.1 (7) | 257 |  |  |
| Iran, 1993-95[32] | Pregnant F | 2006 (11) | EIA | A | 173 (148,201) | 248 (99,964) | 96 (94,99) | 46 (10,74) | 69.8 (9) | 124 | B | C |
|  |  |  |  | B | 163 (145,183) | 69 (45,95) | -- | 67 (47,85) | 76.4 (9) | 127 |  |  |
|  |  |  |  | C | 174 (151,203) | 174 (151,203) | 96 (95,98) | 53 (35,75) | 70.3 (9) | 120 |  |  |
|  |  |  |  | D | 130 (121,142) | 130 (121,142) | -- | 100 (85,114) | 101 (9) | 148 |  |  |
| Jordan, 1982-3[33] | Cord blood, well child and ANC attendees | 1100 (6) | EIA | A | 1000 (1000,1000) | 27 (6,47) | 84 (82,86) | 0 (0,0) | 135.4 (3) | 181 | D | D |
|  |  |  |  | B | 166 (153,182) | 0 (0,0) | -- | 0 (0,0) | 301.7 (4) | 337 |  |  |
|  |  |  |  | C | 1000 (1000,1000) | 1000 (1000,1000) | 84 (82,86) | 0 (0,0) | 135.4 (4) | 171 |  |  |
|  |  |  |  | D | 124 (113,136) | 124 (113,136) | -- | 110 (92,126) | 374.9 (5) | 405 |  |  |

| **Country, year of study, reference** | **Study population** | **Sample size (no. of age groups)** | **Lab test (cut-off)** | **Cata-lytic model** | **Force of infection (/1000/year)** | | **Sensitivity (%)** | **CRS/ 100,000 live births** | **Loglike-lihood deviance (deg of freedom)** | **AICc** | **Selected model according to criterion:** | |
| --- | --- | --- | --- | --- | --- | --- | --- | --- | --- | --- | --- | --- |
|  |  |  |  |  | **<13 yr olds** | **≥13 yr olds** |  |  |  |  | **1** | **2** |
| Kuwait, <1978[34] | F (child-bearing age) | 1002 (5) | HAI-1:4 | A | 214 (0,658) | 222 (0,941) | 95 (94,100) | 30 (0,189) | 1.7 (2) | 52 | B | C |
|  |  |  |  | B | 217 (168,250) | 15 (0,68) | -- | 16 (0,59) | 2.1 (3) | 32 |  |  |
|  |  |  |  | C | 216 (150,987) | 216 (150,987) | 95 (94,98) | 29 (0,75) | 1.7 (3) | 32 |  |  |
|  |  |  |  | D | 121 (110,134) | 121 (110,134) | -- | 114 (94,132) | 21.3 (4) | 45 |  |  |
| Lebanon, 1980-81[35] | Pregnant F | 65 (3) | SRH & HAI- | A | 559 (0,931) | 0 (0,769) | 92 (86,100) | 0 (0,146) | 1.2 (0) | - | D | D |
|  |  |  |  | B | 205 (111,334) | 0 (0,174) | -- | 0 (0,130) | 1.2 (1) | - |  |  |
|  |  |  |  | C | 989 (114,999) | 989 (114,999) | 92 (86,100) | 0 (0,125) | 1.2 (1) | - |  |  |
|  |  |  |  | D | 118 (86,198) | 118 (86,198) | -- | 118 (38,178) | 3.4 (2) | 16 |  |  |
| Morocco, 1969-1970[36] | Schoolgirls & pregnant F | 544 (6) | HAI-1:10 | A | 125 (101,189) | 32 (11,738) | 100 (86,100) | 81 (15,133) | 8.6 (3) | 50 | A | B |
|  |  |  |  | B | 125 (103,152) | 32 (8,57) | -- | 81 (24,133) | 8.6 (4) | 40 |  |  |
|  |  |  |  | C | 167 (98,999) | 167 (98,999) | 88 (83,96) | 60 (0,155) | 11.7 (4) | 43 |  |  |
|  |  |  |  | D | 86 (77,96) | 86 (77,96) | -- | 179 (158,199) | 20.6 (5) | 47 |  |  |
| Saudi Arabia, 1989[39] | Children in peds clinic, F at Obs. & Gynae | 672 (3) | EIA | A | 188 (152,397) | 21 (0,1000) | 100 (91,100) | 29 (0,56) | 0 (0) | - | B | D |
|  |  |  |  | B | 188 (145,219) | 21 (0,79) | -- | 29 (0,84) | 0 (1) | - |  |  |
|  |  |  |  | C | 239 (161,431) | 239 (161,431) | 93 (91,96) | 21 (1,64) | 0.3 (1) | - |  |  |
|  |  |  |  | D | 121 (108,136) | 121 (108,136) | -- | 113 (92,136) | 11.5 (2) | 30 |  |  |
| Saudi Arabia, 1992-93[40] | Antenatal F | 10824 (6) | ? | A | 180 (154,215) | 103 (67,147) | 93 (92,95) | 55 (35,76) | 23.6 (3) | 81 | A | D |
|  |  |  |  | B | 160 (149,171) | 27 (17,37) | -- | 48 (31,64) | 31.1 (4) | 79 |  |  |
|  |  |  |  | C | 161 (144,192) | 161 (144,192) | 93 (92,94) | 65 (41,83) | 26.7 (4) | 74 |  |  |
|  |  |  |  | D | 91 (89,94) | 91 (89,94) | -- | 168 (163,173) | 220.1 (5) | 263 |  |  |

| **Country, year of study, reference** | **Study population** | **Sample size (no. of age groups)** | **Lab test (cut-off)** | **Cata-lytic model** | **Force of infection (/1000/year)** | | **Sensitivity (%)** | **CRS/ 100,000 live births** | **Loglike-lihood deviance (deg of freedom)** | **AICc** | **Selected model according to criterion:** | |
| --- | --- | --- | --- | --- | --- | --- | --- | --- | --- | --- | --- | --- |
|  |  |  |  |  | **<13 yr olds** | **≥13 yr olds** |  |  |  |  | **1** | **2** |
| Tunisia, <1970[41] | Schoolgirls & pregnant F | 429 (5) | HAI-1:10 | A | 473 (340,1000) | 208 (8,941) | 94 (90,97) | 1 (0,6) | 3.4 (2) | 50 | D | C |
|  |  |  |  | B | 283 (248,329) | 0 (0,33) | -- | 0 (0,12) | 14.8 (3) | 42 |  |  |
|  |  |  |  | C | 475 (342,1000) | 475 (342,1000) | 94 (90,97) | 1 (0,5) | 3.4 (3) | 30 |  |  |
|  |  |  |  | D | 240 (203,289) | 240 (203,289) | -- | 20 (10,35) | 43 (4) | 63 |  |  |
| **Europe** | | | | | | | | | | | | |
| Czech Republic, <1967[25] | Residual sera | 157 (5) | HAI-? | A | 135 (105,463) | 0 (0,1000) | 100 (77,100) | 0 (0,109) | 1.5 (2) | 48 | C | C |
|  |  |  |  | B | 135 (101,161) | 0 (0,48) | -- | 0 (0,116) | 1.5 (3) | 28 |  |  |
|  |  |  |  | C | 219 (122,498) | 219 (122,498) | 82 (75,91) | 28 (0,112) | 2.3 (3) | 29 |  |  |
|  |  |  |  | D | 82 (67,101) | 82 (67,101) | -- | 189 (149,222) | 12.9 (4) | 33 |  |  |
| Denmark, <1967[25] | Residual sera | 118 (3) | HAI-? | A | 235 (203,280) | 15 (0,401) | 96 (92,100) | 13 (0,36) | 12.8 (14) | 93 | A | C |
|  |  |  |  | B | 207 (192,223) | 0 (0,84) | -- | 0 (0,41) | 15 (15) | 92 |  |  |
|  |  |  |  | C | 248 (214,289) | 248 (214,289) | 94 (91,98) | 18 (10,30) | 13.8 (15) | 91 |  |  |
|  |  |  |  | D | 197 (183,213) | 197 (183,213) | -- | 38 (30,47) | 25.8 (16) | 100 |  |  |
| Denmark, 1983[44] | Residual sera | 1442 (17) | EIA | A | 103 (0,948) | 49 (0,798) | 100 (83,100) | 130 (0,419) | 0 (0) | - | B | D |
|  |  |  |  | B | 103 (22,171) | 49 (0,148) | -- | 130 (0,373) | 0 (1) | - |  |  |
|  |  |  |  | C | 104 (66,958) | 104 (66,958) | 92 (81,100) | 142 (0,224) | 0 (1) | - |  |  |
|  |  |  |  | D | 77 (61,99) | 77 (61,99) | -- | 200 (153,234) | 0.4 (2) | 17 |  |  |
| East Germany, 1990[45] | Random sample | 1862 (23) | HAI-? (EIA on neg.) | A | 205 (188,240) | 82 (64,118) | 100 (96,100) | 41 (26,50) | 29.6 (20) | 119 | A | B |
|  |  |  |  | B | 201 (187,217) | 77 (50,114) | -- | 42 (33,50) | 29.6 (21) | 117 |  |  |
|  |  |  |  | C | 223 (193,256) | 223 (193,256) | 97 (94,99) | 26 (16,41) | 34.7 (21) | 122 |  |  |
|  |  |  |  | D | 187 (175,202) | 187 (175,202) | -- | 44 (36,53) | 43.9 (22) | 128 |  |  |

| **Country, year of study, reference** | **Study population** | **Sample size (no. of age groups)** | **Lab test (cut-off)** | **Cata-lytic model** | **Force of infection (/1000/year)** | | **Sensitivity (%)** | **CRS/ 100,000 live births** | **Loglike-lihood deviance (deg of freedom)** | **AICc** | **Selected model according to criterion:** | |
| --- | --- | --- | --- | --- | --- | --- | --- | --- | --- | --- | --- | --- |
|  |  |  |  |  | **<13 yr olds** | **≥13 yr olds** |  |  |  |  | **1** | **2** |
| England, <1967[25] | Residual sera | 294 (5) | HAI-? | A | 111 (89,190) | 8 (0,1000) | 100 (77,100) | 36 (0,134) | 2.2 (2) | 53 | B | B |
|  |  |  |  | B | 111 (88,130) | 8 (0,40) | -- | 36 (0,138) | 2.2 (3) | 33 |  |  |
|  |  |  |  | C | 162 (108,241) | 162 (108,241) | 80 (73,88) | 64 (20,135) | 3.7 (3) | 35 |  |  |
|  |  |  |  | D | 72 (62,85) | 72 (62,85) | -- | 211 (182,233) | 18.8 (4) | 43 |  |  |
| England, 1986-87[46] | Residual sera | 4230 (44) | RH | A | 126 (120,143) | 62 (50,299) | 100 (92,100) | 105 (63,114) | 57.7 (41) | 231 | A | B |
|  |  |  |  | B | 126 (119,132) | 62 (50,76) | -- | 105 (93,117) | 57.7 (42) | 229 |  |  |
|  |  |  |  | C | 134 (125,144) | 134 (125,144) | 95 (93,97) | 94 (83,107) | 59.5 (42) | 230 |  |  |
|  |  |  |  | D | 110 (105,115) | 110 (105,115) | -- | 132 (123,141) | 104.9 (43) | 273 |  |  |
| Finland, 1979[47] | Sera sent for rubella test | 751 (12) | RH | A | 82 (72,93) | 187 (141,250) | 89 (88,91) | 164 (138,186) | 15.6 (9) | 92 | B | D |
|  |  |  |  | B | 88 (80,96) | 59 (51,68) | -- | 166 (144,188) | 52.9 (10) | 126 |  |  |
|  |  |  |  | C | 95 (88,104) | 95 (88,104) | 93 (92,95) | 160 (142,176) | 35.7 (10) | 109 |  |  |
|  |  |  |  | D | 74 (72,76) | 74 (72,76) | -- | 206 (202,211) | 68.2 (11) | 138 |  |  |
| France, <1967[25] | Residual sera | 201 (5) | HAI-? | A | 146 (90,233) | 766 (0,1000) | 81 (75,100) | 21 (0,128) | 2.1 (2) | 51 | B | C |
|  |  |  |  | B | 110 (80,140) | 17 (0,58) | -- | 66 (0,178) | 3.8 (3) | 33 |  |  |
|  |  |  |  | C | 149 (95,247) | 149 (95,247) | 83 (75,92) | 76 (19,160) | 2.9 (3) | 32 |  |  |
|  |  |  |  | D | 74 (61,91) | 74 (61,91) | -- | 205 (169,233) | 12.7 (4) | 35 |  |  |

| **Country, year of study, reference** | **Study population** | **Sample size (no. of age groups)** | **Lab test (cut-off)** | **Cata-lytic model** | **Force of infection (/1000/year)** | | **Sensitivity (%)** | **CRS/ 100,000 live births** | **Loglike-lihood deviance (deg of freedom)** | **AICc** | **Selected model according to criterion:** | |
| --- | --- | --- | --- | --- | --- | --- | --- | --- | --- | --- | --- | --- |
|  |  |  |  |  | **<13 yr olds** | **≥13 yr olds** |  |  |  |  | **1** | **2** |
| Kyrgyzstan, 2001[48] | F attending women’s clinics | 964 (5) | EIA | A | 55 (0,884) | 1000 (0,1000) | 93 (92,100) | 41 (0,140) | 5.4 (2) | 57 | D | C |
|  |  |  |  | B | 210 (178,229) | 0 (0,28) | -- | 0 (0,38) | 5.7 (3) | 38 |  |  |
|  |  |  |  | C | 362 (187,999) | 362 (187,999) | 93 (91,95) | 3 (0,44) | 5.7 (3) | 38 |  |  |
|  |  |  |  | D | 102 (93,113) | 102 (93,113) | -- | 147 (127,164) | 46.1 (4) | 71 |  |  |
| Romania, <1989[49] | Healthy F | 5030 (5) | HAI-1:20 | A | 166 (144,995) | 0 (0,939) | 88 (76,93) | 0 (0,0) | 6.3 (2) | 71 | D | D |
|  |  |  |  | B | 116 (112,121) | 0 (0,0) | -- | 0 (0,0) | 6.3 (3) | 51 |  |  |
|  |  |  |  | C | 964 (860,999) | 964 (860,999) | 77 (76,78) | 0 (0,0) | 6.3 (3) | 51 |  |  |
|  |  |  |  | D | 54 (52,56) | 54 (52,56) | -- | 248 (245,251) | 319.4 (4) | 358 |  |  |
| Turkey, 1998[50] | F of reproductive age | 467 (6) | EIA | A | 356 (19,999) | 0 (0,1000) | 99 (97,100) | 0 (0,63) | 4 (3) | 34 | D | D |
|  |  |  |  | B | 325 (261,385) | 0 (0,48) | -- | 0 (0,15) | 4 (4) | 24 |  |  |
|  |  |  |  | C | 995 (246,999) | 995 (246,999) | 98 (97,99) | 0 (0,19) | 4 (4) | 24 |  |  |
|  |  |  |  | D | 153 (129,199) | 153 (129,199) | -- | 72 (37,102) | 23.2 (5) | 38 |  |  |
| Turkey, 2003-04[51] | Pregnant F | 803 (6) | EIA | A | 695 (31,984) | 0 (0,1000) | 94 (93,100) | 0 (0,55) | 5.6 (3) | 44 | D | D |
|  |  |  |  | B | 231 (209,255) | 0 (0,1) | -- | 0 (0,2) | 5.6 (4) | 34 |  |  |
|  |  |  |  | C | 962 (287,999) | 962 (287,999) | 94 (93,96) | 0 (0,10) | 5.6 (4) | 34 |  |  |
|  |  |  |  | D | 112 (101,126) | 112 (101,126) | -- | 128 (107,149) | 42.5 (5) | 66 |  |  |
| Turkey, 2005[52] | Women attending health care centres | 607 (4) | EIA | A | 143 (0,985) | 0 (0,1000) | 66 (52,97) | 0 (0,256) | 1 (1) | -- | D | D |
|  |  |  |  | B | 64 (54,71) | 0 (0,6) | -- | 0 (0,56) | 1 (2) | 39 |  |  |
|  |  |  |  | C | 674 (116,993) | 674 (116,993) | 55 (51,60) | 0 (0,121) | 1 (2) | 39 |  |  |
|  |  |  |  | D | 26 (24,29) | 26 (24,29) | -- | 251 (242,257) | 41.4 (3) | 67 |  |  |

| **Country, year of study, reference** | **Study population** | **Sample size (no. of age groups)** | **Lab test (cut-off)** | **Cata-lytic model** | **Force of infection (/1000/year)** | | **Sensitivity (%)** | **CRS/ 100,000 live births** | **Loglike-lihood deviance (deg of freedom)** | **AICc** | **Selected model according to criterion:** | |
| --- | --- | --- | --- | --- | --- | --- | --- | --- | --- | --- | --- | --- |
|  |  |  |  |  | **<13 yr olds** | **≥13 yr olds** |  |  |  |  | **1** | **2** |
| **South East Asia** | | | | | | | | | | | | |
| Thailand, 1978[59] | F of childbearing age | 300 (6) | HAI-1:8 | A | 84 (0,108) | 63 (13,599) | 87 (75,100) | 176 (56,387) | 2.9 (3) | 45 | A | D |
|  |  |  |  | B | 74 (46,105) | 30 (6,56) | -- | 150 (33,267) | 2.9 (4) | 35 |  |  |
|  |  |  |  | C | 83 (49,184) | 83 (49,184) | 85 (75,100) | 186 (46,255) | 2.9 (4) | 35 |  |  |
|  |  |  |  | D | 50 (44,59) | 50 (44,59) | -- | 254 (239,261) | 5.9 (5) | 33 |  |  |
| **Western Pacific** | | | | | | | | | | | | |
| Australia, <1967[25] | Residual sera | 207 (5) | HAI-? | A | 194 (101,774) | 82 (0,1000) | 81 (71,100) | 46 (0,119) | 0.9 (2) | 51 | D | C |
|  |  |  |  | B | 117 (89,140) | 6 (0,40) | -- | 27 (0,131) | 1.7 (3) | 31 |  |  |
|  |  |  |  | C | 207 (122,790) | 207 (122,790) | 79 (70,88) | 33 (0,112) | 1 (3) | 31 |  |  |
|  |  |  |  | D | 75 (61,92) | 75 (61,92) | -- | 203 (167,234) | 16.2 (4) | 39 |  |  |
| China, 1979-80[67] | Residual sera | 16658 (11) | HAI-? | A | 295 (283,311) | 2 (0,24) | 98 (97,99) | 1 (0,8) | 134.2 (8) | 217 | A | D |
|  |  |  |  | B | 268 (262,275) | 0 (0,0) | -- | 0 (0,0) | 153.1 (9) | 232 |  |  |
|  |  |  |  | C | 318 (306,330) | 318 (306,330) | 96 (96,97) | 7 (5,8) | 150.5 (9) | 229 |  |  |
|  |  |  |  | D | 205 (199,212) | 205 (199,212) | -- | 34 (31,37) | 1332.3 (10) | 1408 |  |  |
| Fiji, <1973[60] | Sera from F in all ages | 1174 (9) | HAI-1:4 | A | 29 (23,35) | 81 (63,120) | 97 (92,100) | 364 (332,391) | 62.1 (6) | 108 | C | D |
|  |  |  |  | B | 29 (24,35) | 68 (59,81) | -- | 356 (325,387) | 64.6 (7) | 105 |  |  |
|  |  |  |  | C | 45 (42,49) | 45 (42,49) | 100 (100,100) | 260 (255,262) | 93 (7) | 134 |  |  |
|  |  |  |  | D | 45 (42,49) | 45 (42,49) | -- | 260 (255,262) | 93 (8) | 130 |  |  |

| **Country, year of study, reference** | **Study population** | **Sample size (no. of age groups)** | **Lab test (cut-off)** | **Cata-lytic model** | **Force of infection (/1000/year)** | | **Sensitivity (%)** | **CRS/ 100,000 live births** | **Loglike-lihood deviance (deg of freedom)** | **AICc** | **Selected model according to criterion:** | |
| --- | --- | --- | --- | --- | --- | --- | --- | --- | --- | --- | --- | --- |
|  |  |  |  |  | **<13 yr olds** | **≥13 yr olds** |  |  |  |  | **1** | **2** |
| Japan (Sapporo), <1967[25] | Residual sera | 188 (5) | HAI-? | A | 1000 (109,1000) | 820 (0,1000) | 50 (44,62) | 0 (0,18) | 15.6 (2) | 65 | D | D |
|  |  |  |  | B | 62 (50,75) | 0 (0,0) | -- | 0 (0,0) | 19.6 (3) | 49 |  |  |
|  |  |  |  | C | 1000 (174,1000) | 1000 (174,1000) | 50 (44,59) | 0 (0,53) | 15.6 (3) | 45 |  |  |
|  |  |  |  | D | 34 (27,41) | 34 (27,41) | -- | 262 (253,264) | 47.3 (4) | 70 |  |  |
| Japan (Ohtsu), <1967[25] | Residual sera | 155 (5) | HAI-? | A | 47 (8,98) | 171 (2,1000) | 38 (28,100) | 262 (13,417) | 0.1 (2) | 48 | B | D |
|  |  |  |  | B | 18 (6,32) | 15 (0,31) | -- | 187 (0,345) | 0.5 (3) | 28 |  |  |
|  |  |  |  | C | 28 (13,128) | 28 (13,128) | 66 (29,100) | 255 (103,264) | 0.5 (3) | 28 |  |  |
|  |  |  |  | D | 16 (12,22) | 16 (12,22) | -- | 206 (168,235) | 0.6 (4) | 21 |  |  |
| Malaysia, <1972[61] | F hospital patients, pregnant F | 697 (6) | HAI-1:10 | A | 54 (42,1000) | 22 (14,39) | 100 (62,100) | 159 (0,225) | 22.8 (3) | 70 | B | B |
|  |  |  |  | B | 54 (42,66) | 22 (12,32) | -- | 159 (91,225) | 22.8 (4) | 60 |  |  |
|  |  |  |  | C | 51 (35,1000) | 51 (35,1000) | 86 (59,100) | 252 (0,264) | 30.1 (4) | 67 |  |  |
|  |  |  |  | D | 38 (34,42) | 38 (34,42) | -- | 264 (262,264) | 30.9 (5) | 63 |  |  |
| Singapore, 1975-79[62] | Pregnant women and F (child-bearing age) | 2284 (5) | HAI-1:8 | A | 13 (0,89) | 244 (106,364) | 55 (52,60) | 345 (156,447) | 4.7 (2) | 65 | B | C |
|  |  |  |  | B | 47 (35,58) | 11 (1,22) | -- | 105 (10,197) | 10.7 (3) | 51 |  |  |
|  |  |  |  | C | 86 (54,145) | 86 (54,145) | 59 (53,70) | 179 (82,248) | 7.9 (3) | 49 |  |  |
|  |  |  |  | D | 28 (27,30) | 28 (27,30) | -- | 255 (252,258) | 22 (4) | 56 |  |  |
| **Country, year of study, reference** | **Study population** | **Sample size (no. of age groups)** | **Lab test (cut-off)** | **Cata-lytic model** | **Force of infection (/1000/year)** | | **Sensitivity (%)** | **CRS/ 100,000 live births** | **Loglike-lihood deviance (deg of freedom)** | **AICc** | **Selected model according to criterion:** | |
|  |  |  |  |  | **<13 yr olds** | **≥13 yr olds** |  |  |  |  | **1** | **2** |
| Taiwan, 1984[63] | F on maternity ward | 154 (5) | HAI:1-8 | A | 90 (0,442) | 68 (0,674) | 100 (85,100) | 167 (0,471) | 0.5 (2) | 43 | B | D |
|  |  |  |  | B | 90 (0,176) | 68 (0,161) | -- | 167 (0,475) | 0.5 (3) | 23 |  |  |
|  |  |  |  | C | 79 (67,984) | 79 (67,984) | 100 (84,100) | 195 (0,221) | 0.6 (3) | 23 |  |  |
|  |  |  |  | D | 79 (66,97) | 79 (66,97) | -- | 195 (156,224) | 0.6 (4) | 16 |  |  |
| Taiwan, 1984-6[64] | Sera from older girls and women of child-bearing age | 2030 (4) | HAI | A | 122 (99,150) | 756 (390,1000) | 62 (59,66) | 30 (14,70) | 5.5 (1) | - | B | D |
|  |  |  |  | B | 62 (56,68) | 28 (10,47) | -- | 168 (72,227) | 12.1 (2) | 55 |  |  |
|  |  |  |  | C | 102 (70,140) | 102 (70,140) | 72 (64,88) | 147 (87,216) | 11.8 (2) | 54 |  |  |
|  |  |  |  | D | 55 (52,58) | 55 (52,58) | -- | 246 (239,251) | 20 (3) | 51 |  |  |

**Notes:** See for the definitions of the abbreviations.

Figure Ba: Comparison between model predictions of the percentage susceptible and the percentage seronegative to rubella obtained using the four types of catalytic model (denoted by the lines labelled A, B, C and D), and that observed in different settings in the African WHO region. The crosses show the observed percentage seronegative together with 95% (exact) confidence intervals

Figure Bb: Comparison between model predictions of the percentage susceptible and the percentage seronegative to rubella obtained using the four types of catalytic model (denoted by the lines labelled A, B, C and D), and that observed in different settings in the African WHO region. The crosses show the observed percentage seronegative together with 95% (exact) confidence intervals.

Figure Bc: Comparison between model predictions of the percentage susceptible and the percentage seronegative to rubella obtained using the four types of catalytic model (denoted by the lines labelled A, B, C and D), and that observed in different settings in the African WHO region. The crosses show the observed percentage seronegative together with 95% (exact) confidence intervals.

Figure : Comparison between model predictions of the percentage susceptible and the percentage seronegative to rubella obtained using the four types of catalytic model (denoted by the lines labelled A, B, C and D), and that observed in different settings in the Eastern Mediterranean WHO region. The crosses show the observed percentage seronegative together with 95% (exact) confidence intervals.

Figure Da: Comparison between model predictions of the percentage susceptible and the percentage seronegative to rubella obtained using the four types of catalytic model (denoted by the lines labelled A, B, C and D), and that observed in different settings in the South East Asian WHO region. The crosses show the observed percentage seronegative, together with 95% (exact) confidence intervals.

Figure Db: Comparison between model predictions of the percentage susceptible and the percentage seronegative to rubella obtained using the four types of catalytic model (denoted by the lines labelled A, B, C and D), and that observed in different settings in the South East Asian and Western Pacific regions. The crosses show the observed percentage seronegative together with 95% (exact) confidence intervals.

Figure : Comparison between model predictions of the percentage susceptible and the percentage seronegative to rubella obtained using the four types of catalytic model (denoted by the lines labelled A, B, C and D), and that observed for the two datasets for which the selected catalytic models fitted poorly. The crosses show the observed percentage seronegative, together with 95% (exact) confidence intervals.

# E: Estimates of the CRS incidence

Figure Fa: Estimates of the incidence of CRS per 100,000 live births among 15-44 year olds in the African, American and Eastern Mediterranean WHO Regions in 2000 and 2010. Countries in which rubella vaccination had not been introduced by the year 2010 (either into the routine schedule or during a campaign) are indicated using an asterisk.

Figure Fb: Estimates of the incidence of CRS per 100,000 live births among 15-44 year olds in the European, South East Asian and Western Pacific WHO Regions in 2000 and 2010. Countries in which rubella vaccination had not been introduced by the year 2010 (either into the routine schedule or during a campaign) are indicated using an asterisk.

Table : Estimated numbers of CRS cases per 100,000 live births and overall number by WHO region and year. The different columns reflect estimates obtained by using datasets for countries based on WHO/geographical region or GBD regions for settings which did not have serological datasets predating the introduction of RCV. The values in parentheses reflect the values at the lowest and highest limits of the 95% CI. The reduction in the burden in CRS between the years 2004 and 2005 is attributable to a change in the value for the fertility rate that is used in these calculations, which uses the average value for the period 2000-2004 for each year in this period and the average value for the period 2005-2010 for each year in subsequent years.

| **Region** | **Year** | **WHO grouping** | | **GBD grouping** | | |
| --- | --- | --- | --- | --- | --- | --- |
|  |  | **CRS incidence per 100,000 live births** | **Total number of CRS cases** | **CRS incidence per 100,000 live births** | **Total number of CRS cases** | |
| **AFRO** | 1996 | 115 (55,231) | 28315 (13443,57421) | 121 (74,181) | 29976 (18488,46525) | |
|  | 2000 | 116 (55,232) | 30464 (14411,61846) | 121 (74,182) | 32317 (19812,50053) | |
|  | 2001 | 116 (55,233) | 31388 (14829,63821) | 121 (74,182) | 33313 (20403,51690) | |
|  | 2002 | 116 (55,233) | 32354 (15256,65870) | 122 (74,183) | 34350 (21021,53415) | |
|  | 2003 | 116 (55,234) | 33344 (15690,67970) | 122 (74,183) | 35400 (21638,55161) | |
|  | 2004 | 116 (55,234) | 34355 (16132,70130) | 122 (74,184) | 36475 (22269,56946) | |
|  | 2005 | 116 (56,235) | 33696 (15813,68886) | 122 (74,184) | 35770 (21574,55277) | |
|  | 2006 | 116 (56,235) | 34682 (16250,71023) | 122 (74,185) | 36817 (22196,57039) | |
|  | 2007 | 116 (56,235) | 35662 (16695,73202) | 122 (74,185) | 37891 (22834,58836) | |
|  | 2008 | 116 (56,235) | 36657 (17146,75411) | 122 (74,185) | 38982 (23486,60661) | |
|  | 2009 | 116 (56,235) | 37675 (17603,77635) | 122 (74,186) | 40090 (24164,62438) | |
|  | 2010 | 116 (56,235) | 38712 (18063,79852) | 122 (74,186) | 41194 (24844,64198) | |
| **AMRO** | 1996 | 56 (24,104) | 10640 (4394,19867) | 68 (34,105) | 13337 (6730,20302) | |
|  | 2000 | 11 (6,23) | 2514 (1160,4990) | 12 (7,23) | 2633 (1379,5017) | |
|  | 2001 | 6 (1,17) | 1164 (276,3092) | 6 (2,12) | 1369 (567,2714) | |
|  | 2002 | 2 (0,13) | 541 (8,2623) | 3 (0,7) | 685 (73,1434) | |
|  | 2003 | 2 (0,21) | 421 (1,4117) | 2 (0,11) | 464 (18,2166) | |
|  | 2004 | 1 (0,24) | 168 (0,4586) | 1 (0,12) | 153 (4,2303) | |
|  | 2005 | <1 (0,1) | 25 (0,170) | <1 (0,1) | 12 (1,99) | |
|  | 2006 | <0.1 (0,2) | 4 (0,187) | <0.1 (0,0.2) | 1 (0,34) | |
|  | 2007 | <0.01 (0,1) | 1 (0,62) | <0.1 (0,0.2) | 1 (0,30) | |
|  | 2008 | <0.01 (0,1) | 1 (0,94) | <0.01 (0,1) | <1 (0,93) | |
|  | 2009 | <0.01 (0,1) | <1 (0,198) | <0.01 (0,1) | <1 (0,198) |  |
|  | 2010 | <0.01 (0,1) | <1 (0,136) | <0.01 (0,1) | <1 (0,136) | |
| **EMRO** | 1996 | 56 (22,106) | 7625 (2577,15290) | 62 (31,105) | 8708 (4078,14945) | |
|  | 2000 | 42 (16,82) | 6216 (1927,12580) | 47 (23,83) | 6874 (3036,12708) | |
|  | 2001 | 39 (15,77) | 5933 (1830,12202) | 43 (21,79) | 6606 (2832,12672) | |
|  | 2002 | 37 (15,74) | 5882 (1794,12088) | 41 (19,77) | 6480 (2744,12713) | |
|  | 2003 | 30 (8,67) | 5336 (1217,11607) | 34 (13,72) | 5934 (2092,12423) | |
|  | 2004 | 27 (6,63) | 5033 (977,11393) | 31 (10,67) | 5658 (1750,12134) | |
|  | 2005 | 26 (5,62) | 4609 (839,10652) | 30 (9,65) | 5209 (1508,11301) | |
|  | 2006 | 26 (4,61) | 4719 (748,10985) | 29 (8,65) | 5330 (1489,11620) | |
|  | 2007 | 26 (4,61) | 4828 (756,11297) | 29 (7,65) | 5419 (1508,11974) | |
|  | 2008 | 25 (4,61) | 4977 (776,11643) | 29 (7,65) | 5586 (1539,12350) | |
|  | 2009 | 25 (4,61) | 5133 (799,12000) | 29 (7,65) | 5762 (1586,12732) | |
|  | 2010 | 25 (4,61) | 5294 (827,12358) | 29 (7,65) | 5938 (1639,13115) | |

| **Region** | **Year** | **WHO grouping** | | **GBD grouping** | |
| --- | --- | --- | --- | --- | --- |
|  |  | **CRS incidence per 100,000 live births** | **Total number of CRS cases** | **CRS incidence per 100,000 live births** | **Total number of CRS cases** |
| **EURO** | 1996 | 65 (14,133) | 8155 (1839,15349) | 108 (99,130) | 11217 (9596,14211) |
|  | 2000 | 45 (6,114) | 6004 (1030,13266) | 79 (70,97) | 8493 (7089,11075) |
|  | 2001 | 31 (5,86) | 4811 (877,10942) | 51 (39,71) | 6176 (4554,9067) |
|  | 2002 | 25 (6,73) | 4315 (900,9991) | 37 (20,60) | 5073 (3076,8221) |
|  | 2003 | 23 (5,68) | 4167 (887,9558) | 34 (16,57) | 4737 (2651,7942) |
|  | 2004 | 21 (5,57) | 3830 (845,8488) | 26 (12,48) | 4030 (2291,7020) |
|  | 2005 | 20 (5,46) | 3421 (762,7039) | 20 (11,40) | 3304 (1902,6043) |
|  | 2006 | 9 (2,28) | 1483 (275,4216) | 9 (4,28) | 1406 (738,4010) |
|  | 2007 | 4 (0,17) | 629 (32,2247) | 3 (2,15) | 528 (336,2018) |
|  | 2008 | 3 (0,13) | 415 (3,1578) | 2 (1,9) | 434 (311,1098) |
|  | 2009 | 1 (0,6) | 179 (2,651) | 1 (0,6) | 108 (49,597) |
|  | 2010 | 1 (0,5) | 98 (1,507) | 1 (0,6) | 74 (9,525) |
| **SEARO** | 1996 | 130 (43,251) | 50128 (14587,96435) | 153 (52,261) | 57718 (17649,100090) |
|  | 2000 | 126 (39,246) | 48252 (13196,93822) | 149 (48,258) | 55585 (16228,97150) |
|  | 2001 | 127 (40,250) | 49121 (13747,95800) | 149 (51,259) | 56522 (17160,99344) |
|  | 2002 | 126 (39,248) | 49995 (13706,97141) | 148 (49,257) | 57204 (16666,100990) |
|  | 2003 | 126 (38,245) | 50863 (14028,98629) | 147 (48,256) | 57804 (16938,102631) |
|  | 2004 | 127 (38,248) | 51836 (13800,100761) | 148 (49,257) | 58868 (17433,104390) |
|  | 2005 | 126 (38,245) | 46997 (12609,91745) | 147 (46,256) | 53485 (15253,94892) |
|  | 2006 | 123 (34,241) | 47136 (11421,92047) | 144 (40,251) | 53723 (14036,95305) |
|  | 2007 | 122 (31,239) | 47470 (11155,92973) | 143 (39,250) | 54225 (13797,96543) |
|  | 2008 | 121 (31,238) | 47963 (11201,94307) | 142 (39,250) | 54861 (13937,97824) |
|  | 2009 | 121 (31,238) | 48613 (11189,95655) | 141 (39,250) | 55514 (13977,99185) |
|  | 2010 | 121 (31,238) | 49229 (11204,96976) | 141 (38,250) | 56166 (14144,100485) |
| **WPRO (excluding China)** | 1996 | 118 (58,225) | 11368 (5137,21938) | 118 (71,222) | 11424 (6949,22046) |
|  | 2000 | 117 (60,206) | 10922 (5020,20115) | 125 (74,222) | 11182 (6882,21587) |
|  | 2001 | 117 (59,207) | 11037 (5048,20484) | 123 (72,223) | 11162 (6677,21948) |
|  | 2002 | 116 (59,213) | 11086 (5072,21173) | 120 (74,223) | 11329 (7064,22414) |
|  | 2003 | 114 (58,221) | 11215 (5169,21917) | 118 (71,223) | 11363 (6785,22762) |
|  | 2004 | 113 (58,221) | 11420 (5126,22150) | 118 (73,223) | 11595 (7121,23306) |
|  | 2005 | 111 (57,209) | 10551 (4698,20424) | 114 (70,219) | 10643 (6233,21522) |
|  | 2006 | 109 (54,203) | 10510 (4552,20359) | 112 (69,218) | 10606 (6467,21711) |
|  | 2007 | 109 (53,200) | 10569 (4509,20480) | 110 (66,216) | 10627 (6070,21903) |
|  | 2008 | 109 (53,196) | 10689 (4522,20676) | 111 (68,215) | 10783 (6481,22219) |
|  | 2009 | 107 (52,195) | 10658 (4529,20884) | 108 (63,213) | 10692 (6012,22393) |
|  | 2010 | 90 (46,195) | 8889 (4010,21118) | 91 (54,213) | 8833 (5184,22681) |
| **WPRO (including China)** | 1996 | 30 (15,55) | 11541 (5268,21980) | 29 (18,55) | 11645 (7095,22325) |
|  | 2000 | 30 (15,52) | 11084 (5328,20167) | 32 (19,56) | 11381 (7035,21802) |
|  | 2001 | 30 (15,53) | 11163 (5372,20599) | 31 (18,56) | 11393 (6858,22063) |
|  | 2002 | 29 (15,54) | 11242 (5300,21277) | 31 (19,56) | 11536 (7149,22634) |
|  | 2003 | 29 (15,56) | 11392 (5284,21940) | 30 (18,56) | 11579 (6966,22962) |
|  | 2004 | 29 (15,56) | 11565 (5290,22339) | 30 (19,57) | 11827 (7240,23515) |
|  | 2005 | 29 (15,53) | 10710 (4839,20583) | 29 (18,56) | 10845 (6414,21679) |
|  | 2006 | 28 (14,52) | 10684 (4635,20590) | 29 (18,56) | 10835 (6584,21873) |
|  | 2007 | 28 (14,51) | 10774 (4611,20595) | 29 (17,55) | 10835 (6236,22113) |
|  | 2008 | 28 (14,50) | 10725 (4573,20722) | 28 (17,55) | 10851 (6520,22255) |
|  | 2009 | 27 (13,50) | 10660 (4529,20884) | 28 (16,55) | 10694 (6013,22394) |
|  | 2010 | 23 (12,50) | 8889 (4010,21118) | 23 (14,55) | 8833 (5184,22681) |

| **Region** | **Year** | **WHO grouping** | | **GBD grouping** | |
| --- | --- | --- | --- | --- | --- |
|  |  | **CRS incidence per 100,000 live births** | **Total number of CRS cases** | **CRS incidence per 100,000 live births** | **Total number of CRS cases** |
| **Global** | 1996 | -- | 119224 (72119,169107) | -- | 133422 (88796,177332) |
|  | 2000 |  | 107156 (62121,154446) |  | 118331 (75525,163652) |
|  | 2001 |  | 106508 (60618,155183) |  | 116460 (71977,163067) |
|  | 2002 |  | 107408 (59964,157276) |  | 116122 (71314,162864) |
|  | 2003 |  | 108854 (60061,159270) |  | 116664 (70544,164815) |
|  | 2004 |  | 110271 (60553,162017) |  | 118115 (71916,167908) |
|  | 2005 |  | 101841 (55603,149394) |  | 109399 (66099,154955) |
|  | 2006 |  | 101778 (54629,150035) |  | 108948 (65034,155067) |
|  | 2007 |  | 102304 (53638,152542) |  | 109643 (63325,156382) |
|  | 2008 |  | 103845 (54373,156443) |  | 111570 (65732,160816) |
|  | 2009 |  | 105212 (55087,158598) |  | 112499 (64267,162105) |
|  | 2010 |  | 105391 (53605,158041) |  | 113254 (65649,162674) |

# F: Sensitivity analyses

## The effect of selective vaccination coverage

Table : Estimates of the regional and global numbers of CRS cases predicted to have been born each year during 1996-2010, assuming that the selective vaccination coverage in countries which had introduced selective vaccination of adolescents was either 50% (medium, as in the base-case model), 10% (low) or 90% (high). The numbers in parentheses reflect the 95% CI, obtained by bootstrapping.

| **Region** | **Year** | **Numbers of CRS cases assuming a vaccination coverage among adolescents of:** | | |
| --- | --- | --- | --- | --- |
|  |  | **50% (Medium)** | **10% (Low)** | **90% (High)** |
| **AFRO** | 1996 | 28315 (13443,57421) | 28316 (13443,57431) | 28315 (13443,57411) |
|  | 2000 | 30464 (14411,61846) | 30465 (14411,61856) | 30461 (14411,61834) |
|  | 2001 | 31388 (14829,63821) | 31391 (14829,63826) | 31386 (14829,63807) |
|  | 2002 | 32354 (15256,65870) | 32359 (15256,65871) | 32353 (15256,65857) |
|  | 2003 | 33344 (15690,67970) | 33348 (15690,67972) | 33343 (15690,67961) |
|  | 2004 | 34355 (16132,70130) | 34358 (16132,70133) | 34354 (16132,70125) |
|  | 2005 | 33696 (15813,68886) | 33698 (15813,68890) | 33695 (15813,68884) |
|  | 2006 | 34682 (16250,71023) | 34683 (16250,71029) | 34681 (16250,71021) |
|  | 2007 | 35662 (16695,73202) | 35663 (16695,73209) | 35662 (16695,73199) |
|  | 2008 | 36657 (17146,75411) | 36657 (17146,75419) | 36657 (17146,75407) |
|  | 2009 | 37675 (17603,77635) | 37675 (17603,77639) | 37675 (17603,77629) |
|  | 2010 | 38712 (18063,79852) | 38712 (18063,79854) | 38712 (18063,79848) |
| **AMRO** | 1996 | 10640 (4394,19867) | 11341 (4483,21577) | 9973 (4240,18225) |
|  | 2000 | 2514 (1160,4990) | 2767 (1166,5065) | 2470 (1146,4960) |
|  | 2001 | 1164 (276,3092) | 1193 (276,2897) | 1162 (276,2835) |
|  | 2002 | 541 (8,2623) | 550 (8,2670) | 533 (8,2584) |
|  | 2003 | 421 (1,4117) | 434 (1,4112) | 418 (1,4102) |
|  | 2004 | 168 (0,4586) | 171 (0,4586) | 168 (0,4579) |
|  | 2005 | 25 (0,170) | 26 (0,284) | 17 (0,130) |
|  | 2006 | 4 (0,187) | 4 (0,167) | 1 (0,34) |
|  | 2007 | 1 (0,62) | 2 (0,69) | 1 (0,32) |
|  | 2008 | 1 (0,94) | 2 (0,96) | <1 (0,93) |
|  | 2009 | <1 (0,198) | 1 (0,200) | <1 (0,198) |
|  | 2010 | <1 (0,136) | 2 (0,136) | <1 (0,136) |
| **EMRO** | 1996 | 7625 (2577,15290) | 7716 (2634,15479) | 7545 (2526,15166) |
|  | 2000 | 6216 (1927,12580) | 6290 (1986,12719) | 6106 (1871,12444) |
|  | 2001 | 5933 (1830,12202) | 6085 (1880,12373) | 5845 (1776,12045) |
|  | 2002 | 5882 (1794,12088) | 6019 (1905,12298) | 5730 (1688,11890) |
|  | 2003 | 5336 (1217,11607) | 5425 (1249,11752) | 5274 (1206,11474) |
|  | 2004 | 5033 (977,11393) | 5047 (980,11414) | 5030 (973,11374) |
|  | 2005 | 4609 (839,10652) | 4610 (839,10652) | 4607 (838,10652) |
|  | 2006 | 4719 (748,10985) | 4720 (749,10985) | 4719 (747,10984) |
|  | 2007 | 4828 (756,11297) | 4828 (757,11297) | 4828 (756,11297) |
|  | 2008 | 4977 (776,11643) | 4978 (776,11643) | 4977 (776,11643) |
|  | 2009 | 5133 (799,12000) | 5133 (799,12001) | 5133 (799,12000) |
|  | 2010 | 5294 (827,12358) | 5294 (827,12359) | 5294 (826,12358) |

| **Region** | **Year** | **Numbers of CRS cases assuming a vaccination coverage among adolescents of:** | | |
| --- | --- | --- | --- | --- |
|  |  | **50% (Medium)** | **10% (Low)** | **90% (High)** |
| **EURO** | 1996 | 8155 (1839,15349) | 8401 (1869,17772) | 8006 (1818,14875) |
|  | 2000 | 6004 (1030,13266) | 6241 (1152,13394) | 5871 (997,12048) |
|  | 2001 | 4811 (877,10942) | 5046 (941,10712) | 4641 (833,9945) |
|  | 2002 | 4315 (900,9991) | 4563 (875,9500) | 4193 (825,9045) |
|  | 2003 | 4167 (887,9558) | 4420 (863,8986) | 4034 (825,8651) |
|  | 2004 | 3830 (845,8488) | 4018 (864,8004) | 3760 (817,7782) |
|  | 2005 | 3421 (762,7039) | 3587 (786,6703) | 3323 (777,6737) |
|  | 2006 | 1483 (275,4216) | 1836 (299,4404) | 1381 (286,4109) |
|  | 2007 | 629 (32,2247) | 920 (34,2849) | 440 (40,2178) |
|  | 2008 | 415 (3,1578) | 459 (3,2151) | 350 (4,1494) |
|  | 2009 | 179 (2,651) | 271 (2,1058) | 96 (1,531) |
|  | 2010 | 98 (1,507) | 229 (1,741) | 47 (0,406) |
| **SEARO** | 1996 | 50128 (14587,96435) | 50888 (15063,97234) | 49479 (13379,95830) |
|  | 2000 | 48252 (13196,93822) | 48654 (13658,94485) | 47639 (12523,93091) |
|  | 2001 | 49121 (13747,95800) | 49360 (14200,95973) | 48930 (13265,95331) |
|  | 2002 | 49995 (13706,97141) | 50137 (13796,97533) | 49760 (13436,96958) |
|  | 2003 | 50863 (14028,98629) | 50936 (14333,99045) | 50525 (13274,98520) |
|  | 2004 | 51836 (13800,100761) | 51994 (13907,100812) | 51568 (13659,100321) |
|  | 2005 | 46997 (12609,91745) | 47029 (12922,92003) | 46912 (12176,91697) |
|  | 2006 | 47136 (11421,92047) | 47185 (11478,92055) | 46999 (11377,92003) |
|  | 2007 | 47470 (11155,92973) | 47504 (11184,92990) | 47429 (11132,92948) |
|  | 2008 | 47963 (11201,94307) | 48018 (11278,94308) | 48013 (11163,94302) |
|  | 2009 | 48613 (11189,95655) | 48613 (11238,95653) | 48622 (11169,95652) |
|  | 2010 | 49229 (11204,96976) | 49230 (11184,96958) | 49210 (11318,96972) |
| **WPRO** | 1996 | 11368 (5137,21938) | 12163 (5862,22339) | 11278 (5064,21357) |
|  | 2000 | 10922 (5020,20115) | 11612 (5659,20834) | 10652 (4766,19729) |
|  | 2001 | 11037 (5048,20484) | 11766 (5704,21343) | 10808 (4810,20332) |
|  | 2002 | 11086 (5072,21173) | 11962 (5702,22005) | 10957 (4897,20913) |
|  | 2003 | 11215 (5169,21917) | 12143 (5822,22915) | 11122 (4986,21807) |
|  | 2004 | 11420 (5126,22150) | 12438 (5815,22958) | 11290 (5023,21995) |
|  | 2005 | 10551 (4698,20424) | 11511 (5294,21017) | 10506 (4619,20302) |
|  | 2006 | 10510 (4552,20359) | 11347 (4872,20670) | 10617 (4601,20396) |
|  | 2007 | 10569 (4509,20480) | 11263 (4706,20676) | 10760 (4605,20543) |
|  | 2008 | 10689 (4522,20676) | 11152 (4628,20784) | 10724 (4573,20688) |
|  | 2009 | 10658 (4529,20884) | 10892 (4596,20914) | 10658 (4529,20869) |
|  | 2010 | 8889 (4010,21118) | 9088 (4218,21162) | 8889 (4010,21111) |
| **Global** | 1996 | 119224 (72119,169107) | 122125 (74616,171420) | 116953 (70207,166391) |
|  | 2000 | 107156 (62121,154446) | 109017 (63871,156852) | 105685 (60576,152003) |
|  | 2001 | 106508 (60618,155183) | 107715 (61755,156833) | 105219 (58909,153593) |
|  | 2002 | 107408 (59964,157276) | 108852 (61880,158470) | 106140 (58333,155819) |
|  | 2003 | 108854 (60061,159270) | 110394 (61227,160682) | 107768 (59085,157527) |
|  | 2004 | 110271 (60553,162017) | 111897 (61532,163543) | 109193 (59756,161058) |
|  | 2005 | 101841 (55603,149394) | 103489 (56688,150935) | 101314 (55561,148732) |
|  | 2006 | 101778 (54629,150035) | 102628 (55585,152081) | 101432 (54060,149950) |
|  | 2007 | 102304 (53638,152542) | 102750 (54054,153444) | 102099 (53350,152856) |
|  | 2008 | 103845 (54373,156443) | 104336 (54673,157511) | 103691 (54220,155507) |
|  | 2009 | 105212 (55087,158598) | 105617 (55181,158917) | 105107 (54870,158344) |
|  | 2010 | 105391 (53605,158041) | 105838 (53898,158994) | 105232 (53404,158782) |

## The effect of including additional datasets

Table : Estimates of the numbers of CRS cases in the African region and globally predicted to have been born each year during 1996-2010, obtained in the base-case and after including the two datasets (from Niger[98] and Cote D’Ivoire[97]) which had been dropped due to the poor fit of the catalytic models to the data. The numbers in parentheses reflect the 95% CI, obtained by bootstrapping.

|  | **Excluding datasets from Niger[98] and Cote d’Ivoire[97] (base case)** | | **Including datasets from Niger[98] and Cote d’Ivoire[97]** | |
| --- | --- | --- | --- | --- |
| **Year** | **CRS incidence per 100,000 live births** | **Numbers of CRS cases** | **CRS incidence per 100,000 live births** | **Numbers of CRS cases** |
| **Africa** |  |  |  |  |
| 1996 | 115 (55,231) | 28315 (13443,57421) | 124 (63,236) | 30700 (15088,58648) |
| 2000 | 116 (55,232) | 30464 (14411,61846) | 125 (63,237) | 33130 (16316,63022) |
| 2001 | 116 (55,233) | 31388 (14829,63821) | 125 (63,237) | 34149 (16800,65002) |
| 2002 | 116 (55,233) | 32354 (15256,65870) | 125 (63,238) | 35195 (17297,67049) |
| 2003 | 116 (55,234) | 33344 (15690,67970) | 125 (63,238) | 36266 (17807,69117) |
| 2004 | 116 (55,234) | 34355 (16132,70130) | 126 (63,238) | 37357 (18328,71246) |
| 2005 | 116 (56,235) | 33696 (15813,68886) | 126 (64,240) | 36812 (18281,69954) |
| 2006 | 116 (56,235) | 34682 (16250,71023) | 126 (64,240) | 37888 (18823,72041) |
| 2007 | 116 (56,235) | 35662 (16695,73202) | 126 (64,240) | 39003 (19378,74135) |
| 2008 | 116 (56,235) | 36657 (17146,75411) | 126 (64,240) | 40127 (19945,76317) |
| 2009 | 116 (56,235) | 37675 (17603,77635) | 126 (64,240) | 41292 (20521,78541) |
| 2010 | 116 (56,235) | 38712 (18063,79852) | 126 (64,240) | 42460 (21105,80779) |
| **Global** |  |  |  |  |
| 1996 |  | 119224 (72119,169107) |  | 120550 (75072,176115) |
| 2000 |  | 107156 (62121,154446) |  | 108757 (65399,158754) |
| 2001 |  | 106508 (60618,155183) |  | 108294 (63718,158788) |
| 2002 |  | 107408 (59964,157276) |  | 109286 (64244,160594) |
| 2003 |  | 108854 (60061,159270) |  | 110608 (64466,162564) |
| 2004 |  | 110271 (60553,162017) |  | 112733 (65479,165188) |
| 2005 |  | 101841 (55603,149394) |  | 104316 (60937,154006) |
| 2006 |  | 101778 (54629,150035) |  | 104074 (58460,154410) |
| 2007 |  | 102304 (53638,152542) |  | 104546 (57755,157104) |
| 2008 |  | 103845 (54373,156443) |  | 106434 (57756,161225) |
| 2009 |  | 105212 (55087,158598) |  | 107566 (58392,163190) |
| 2010 |  | 105391 (53605,158041) |  | 107866 (57565,163342) |

## Effect of excluding individual datasets

Figure : Estimates of the country-specific CRS incidence per 100,000 live births in Africa in 2010, calculated after excluding individual datasets.

Figure G continued.

Figure G continued.

Figure G continued.

Figure : Estimates of the country-specific CRS incidence per 100,000 live births in the Eastern Mediterranean in 2010, calculated after excluding individual datasets.

Figure H continued

Figure : Estimates of the country-specific CRS incidence per 100,000 live births in South East Asia in 2010, calculated after excluding individual datasets.

Figure : Estimates of the country-specific CRS incidence per 100,000 live births in the Western Pacific in 2010, calculated after excluding individual datasets.

Figure J continued

Figure J continued

Figure : Estimated CRS incidence per 100,000 live births among mothers aged 15-44 years in 2010 in the African, Eastern Mediterranean, South East Asian and Western Pacific regions, calculated after excluding individual datasets.

Figure : Estimated numbers of CRS cases born among mothers aged 15-44 years in 2010 in the African, Eastern Mediterranean, South East Asian and Western Pacific regions, globally, calculated after excluding individual datasets.

Figure : Estimated numbers of cases of CRS born in 2010 globally, calculated after excluding individual datasets.

## Comparison between the current and previous estimates obtained for 1996, for countries that had not introduced rubella-containing vaccine by 1996

In general, differences between the current and previous estimates for 1996, considering the countries analysed in previous analyses[99], varied between regions. The differences resulted mainly from the fact that previous estimates were based on comparatively fewer data sets and thus the incidence for many of the countries was assumed to equal the regional average. The latter was higher in previous analyses for some regions than in current settings, such as the Western Pacific. Other factors included the fact that the current estimates improved on those obtained previously by a) accounting for the sensitivity of the test (where possible), which led to increased or decreased estimates of the CRS burden for Mexico and China respectively, b) weighting the estimates by the age-dependent fertility rate, which led to increased estimates for some countries, e.g. the Philippines, c) accounting for the introduction of rubella-containing vaccine. Comparisons between the two sets of analyses are complicated by our use of the median, based on 1000 bootstrap-derived estimates, for the current analyses, whereas the previous analyses relied on average values. The differences for each region are described in further detail below and are summarised in Table M and Figure N.

For the African region, the previously predicted CRS incidence was slightly lower than that currently predicted: average of 104 vs median 116 per 100,000 live births respectively, corresponding to an average of 22,471 and median of 28,315 cases respectively. This discrepancy is largely due to the increased estimate for Nigeria in the current analyses (Figure N), which results mainly from the high incidence of CRS that was implied by one study[16], which had not been carried out at the time of the previous analyses. Likewise, differences between the estimates for the South East Asian region in 1996 were very small, with most of the small difference being attributable to increased numbers of cases in India, largely due to inclusion of additional datasets (Figure N).

For the American and Eastern Mediterranean regions, the CRS incidence was slightly lower that estimated previously (median of 10,553 vs an average of 15,995 for the American region and median of 7,555 vs average of 12,080 for the Eastern Mediterranean). For the Americas, the estimates obtained for all countries (apart from Mexico) in the current analyses were smaller than those obtained previously. This partly resulted from the fact that, in contrast with previous analyses, the current analyses accounted for the sensitivity of the antibody assay, which resulted in force of infection estimates among older individuals being slightly higher than those obtained previously (e.g. 101 vs 28 per 1000 respectively using the data of Trujillo et al[26]). This increased force of infection, in turn, led to increased estimates of the CRS incidence.

For the Eastern Mediterranean region, the discrepancy was largely due to the estimate for Pakistan being reduced, as compared with that in previous analyses (Figure N), as a result of including datasets which have since become available[37-38]. Pakistan is one of the most populous countries in the Eastern Mediterranean, and this translated into a reduced estimate of the burden for this region.

For the Western Pacific Region, however, the estimates obtained using the two methods were similar (average total numbers of cases of 12,634 vs a median of 11,172, excluding China or 11,395, including China). As shown in Figure N the reduced estimates obtained for three countries (China, Korea and Malaysia) were compensated by increased estimates obtained for the Philippines. The increased estimates for the Philippines resulted from the fact that the numbers of CRS cases in the current analyses were calculated after weighting by an age-dependent fertility rate, which, in many regions, is highest in the youngest maternal age group, in which the incidence of CRS per 100,000 live births is also greatest. The reduced estimates for China resulted from including the sensitivity of the antibody assay when fitting the catalytic model to the data, which led to a slightly increased estimate of the prevalence of infection by child-bearing age. The reduced estimates for Malaysia and Korea resulted from including vaccination in these countries in the current modelling, whereas it was not included in previous analyses.

Table : Comparison between estimates of the CRS incidence per 100,000 live births and the number of CRS cases born in 1996 in different regions. The values in the shaded rows are the values obtained in previous analyses by Cutts and Vynnycky[99]. The values in the rows labelled “restricted” refer to estimates obtained in the current analyses but considering only the countries used in previous analyses. The values in rows labelled “all” refer to estimates obtained in the current analyses and considering all countries.

| **Region** | **Analysis** | **CRS incidence per 100,000 live births** | | | **Number of CRS cases** | | |
| --- | --- | --- | --- | --- | --- | --- | --- |
|  |  | Low* | ”Average”^+^ | High* | Low* | ”Average”^+^ | High* |
| AFRO | Cutts & Vynnycky (1999) [99] | 25 | 104 | 246 | 6127 | **22,471** | 51,472 |
|  | restricted | 55 | 115 | 231 | 13442 | 28308 | 57402 |
|  | all countries | 55 | 115 | 231 | 13443 | 28315 | 57421 |
| AMRO | Cutts & Vynnycky (1999)[99]:  total  Island:  Mainland: | 0  0 | **171**  **175** | 353  598 | 4552 | **15,995** | 35950 |
|  | restricted | 36 | 89 | 167 | 4241 | 10553 | 19765 |
|  | all countries | 24 | 56 | 104 | 4394 | 10640 | 19867 |
| EMRO | Cutts & Vynnycky (1999) [99] | 0 | **77** | 212 | 1008 | **12080** | 30711 |
|  | restricted | 22 | 56 | 106 | 2568 | 7555 | 15135 |
|  | all countries | 22 | 56 | 106 | 2577 | 7625 | 15290 |
| SEARO | Cutts & Vynnycky (1999) [99] | 0 | **136** | 470 | 1016 | **46,621** | 168,910 |
|  | restricted | 43 | 130 | 251 | 14553 | 50076 | 96372 |
|  | all countries | 43 | 130 | 251 | 14587 | 50128 | 96435 |
| WPRO | Cutts & Vynnycky (1999) [99] | 0 | **173** | 302 | 1545 | **12,634** | 21396 |
|  | restricted | 91 | 188 | 351 | 5193 | 11395 | 21179 |
|  | all countries | 58 | 118 | 225 | 5268 | 11541 | 21980 |
| Global | Cutts & Vynnycky (1999) [99] |  | | | 14248 | **109,800** | 308,438 |
|  | restricted |  |  |  | 64303 | 111428 | 161552 |
|  | all countries |  |  |  | 72119 | 119224 | 169107 |

* Low and high refer to the minimum and maximum values presented in the previous analyses[99] and to the lower and upper limits of the 95% CI, based on bootstrapping, for the current analyses.

^+^ The average refers to the “mean” value in the previous analyses[99] and to the median, based on 1000 bootstraps in the current analyses.

Figure N: Comparison between the numbers of CRS cases estimated in the current analyses for 1996 against those obtained for 1996 by Cutts and Vynnycky^34^.

**References**

1. Brown DWJ, Cutts FT, Joseph A. An evaluation of complementary epidemiological methods in a defined population in Southern India for estimating the burden of Congenital Rubella Syndrome. 2004.

2. Cumberland P, Shulman CE, Maple PA, Bulmer JN, Dorman EK, Kawuondo K, et al. Maternal HIV infection and placental malaria reduce transplacental antibody transfer and tetanus antibody levels in newborns in Kenya. J Infect Dis. 2007;196(4):550-7.

3. Scott S, Cumberland P, Shulman CE, Cousens S, Cohen BJ, Brown DW, et al. Neonatal measles immunity in rural Kenya: the influence of HIV and placental malaria infections on placental transfer of antibodies and levels of antibody in maternal and cord serum samples. J Infect Dis. 2005;191(11):1854-60.

4. Shkedy Z, Aerts M, Molenberghs G, Beutels P, Van Damme P. Modelling age-dependent force of infection from prevalence data using fractional polynomials. Stat Med. 2006;25(9):1577-91.

5. Rodier MH, Berthonneau J, Bourgoin A, Giraudeau G, Agius G, Burucoa C, et al. Seroprevalences of Toxoplasma, malaria, rubella, cytomegalovirus, HIV and treponemal infections among pregnant women in Cotonou, Republic of Benin. Acta tropica. 1995;59(4):271-7.

6. Yala F, Biendo M, Odongo I, Kounkou R. [Virological and bacteriological study of materno-fetal infections in Brazzaville]. Bulletin de la Societe de pathologie exotique (1990). 1991;84(5 Pt 5):627-34.

7. Vrinat M, Dutertre J, Helies H, Ropero P. [A serological survey of rubella among pregnant women in Abidjan (author's transl)]. Medecine tropicale : revue du Corps de sante colonial. 1978;38(1):53-7.

8. Ouattara SA, Brettes JP, Kodjo R, Penali K, Gershy-Damet G, Sangare A, et al. [Seroepidemiology of rubella in the Ivory Coast. Geographic distribution]. Bulletin de la Societe de pathologie exotique et de ses filiales. 1987;80(4):655-64.

9. Sandow D, Okubagzhi GS, Arnold U, Denkmann N. Seroepidemiological study in rubella in pregnant women in Gondar Region, northern Ethiopia. Ethiopian medical journal. 1982;20(4):173-8.

10. Cutts FT, Abebe A, Messele T, Dejene A, Enquselassie F, Nigatu W, et al. Sero-epidemiology of rubella in the urban population of Addis Ababa, Ethiopia. Epidemiol Infect. 2000;124(3):467-79.

11. Mefane C. Rubella antibodies in 1737 girls and women in Gabon. Afrique Medicale. 1985;24(226):29-32.

12. Lawn JE, Reef S, Baffoe-Bonnie B, Adadevoh S, Caul EO, Griffin GE. Unseen blindness, unheard deafness, and unrecorded death and disability: congenital rubella in Kumasi, Ghana. American journal of public health. 2000;90(10):1555-61.

13. Dromigny JA, Pecarrere JL, Ollivier G, Leroy F, Zeller HG. [Seroprevalence of rubella in pregnant women at Antananarivo. Study of 853 sera at the Pasteur Institute in Madagascar]. Archives de l'Institut Pasteur de Madagascar. 1996;63(1-2):53-5.

14. Barreto J, Sacramento I, Robertson SE, Langa J, de Gourville E, Wolfson L, et al. Antenatal rubella serosurvey in Maputo, Mozambique. Trop Med Int Health. 2006;11(4):559-64.

15. Odelola HA. Rubella haemagglutination inhibiting antibodies in females of child-bearing age in western Nigeria. Journal of hygiene, epidemiology, microbiology, and immunology. 1978;22(2):190-4.

16. Bukbuk DN, el Nafaty AU, Obed JY. Prevalence of rubella-specific IgG antibody in non-immunized pregnant women in Maiduguri, north eastern Nigeria. Central European journal of public health. 2002;10(1-2):21-3.

17. Amina MD, Oladapo S, Habib S, Adebola O, Bimbo K, Daniel A. Prevalence of rubella IgG antibodies among pregnant women in Zaria, Nigeria. International Health. 2010;2(2):156-9.

18. Dromigny JA, Nabeth P, Perrier Gros Claude JD. Evaluation of the seroprevalence of rubella in the region of Dakar (Senegal). Trop Med Int Health. 2003;8(8):740-3.

19. Corcoran C, Hardie DR. Seroprevalence of rubella antibodies among antenatal patients in the Western Cape. South African medical journal = Suid-Afrikaanse tydskrif vir geneeskunde. 2005;95(9):688-90.

20. Watts T. Rubella antibodies in a sample of Lusaka mothers. Medical journal of Zambia. 1983;17(4):109-10.

21. Dowdle WR, Ferrera W, De Salles Gomes LF, King D, Kourany M, Madalengoitia J, et al. WHO collaborative study on the sero-epidemiology of rubella in Caribbean and Middle and South American populations in 1968. Bull World Health Organ. 1970;42(3):419-22.

22. Pereira F, Uez O. Rubella antibodies in female applicants for premarital health certificates in Mar del Plata, Argentina. Bull Pan Am Health Organ. 1986;20(2):179-85.

23. Souza VA, Moraes JC, Sumita LM, Camargo MC, Fink MC, Hidalgo NT, et al. Prevalence of rubella antibodies in a non-immunized urban population, Sao Paulo, Brazil. The Division of Immunization, CVE. Revista do Instituto de Medicina Tropical de Sao Paulo. 1994;36(4):373-6.

24. Reiche EM, Morimoto HK, Farias GN, Hisatsugu KR, Geller L, Gomes AC, et al. [Prevalence of American trypanosomiasis, syphilis, toxoplasmosis, rubella, hepatitis B, hepatitis C, human immunodeficiency virus infection, assayed through serological tests among pregnant patients, from 1996 to 1998, at the Regional University Hospital Norte do Parana]. Rev Soc Bras Med Trop. 2000;33(6):519-27.

25. Rawls WE, Melnick JL, Bradstreet CM, Bailey M, Ferris AA, Lehmann NI, et al. WHO collaborative study on the sero-epidemiology of rubella. Bull World Health Organ. 1967;37(1):79-88.

26. Gutierrez Trujillo G, Munoz O, Tapia Conyer R, Bustamante Calvillo ME, Alvarez y Munoz MT, Guiscafre Gallardo JP, et al. [The seroepidemiology of rubella in Mexican women. A national probability survey]. Salud publica de Mexico. 1990;32(6):623-31.

27. Yamamoto L, Mejia E, Lopez RM, Gallardo E, Gomez B. Susceptibility to rubella infection in females at high risk. Immune protection associated to population density. Tropical and geographical medicine. 1995;47(6):235-8.

28. Suarez-Ognio L, Adrianzen A, Ortiz A, Martinez C, Whittembury A, Cabezudo E, et al. A rubella serosurvey in postpartum women in the three regions of Peru. Rev Panam Salud Publica. 2007;22(2):110-7.

29. Desinor OY, Anselme RJ, Laender F, Saint-Louis C, Bien-Aime JE. Seroprevalence of antibodies against rubella virus in pregnant women in Haiti. Rev Panam Salud Publica. 2004;15(3):147-50.

30. Pitts OM, Ravenel JM, Finklea JF. Rubella immunity in Trinidad. American journal of epidemiology. 1969;89(3):271-6.

31. Dutta SR, Atrash HK, Mathew L, Mathew PP, Mahmood RA. Seroepidemiology of rubella in Bahrain. Int J Epidemiol. 1985;14(4):618-23.

32. Modarres S, Modarres S, Oskoii NN. The immunity of children and adult females to rubella virus infection in Tehran. Iranian journal of medical sciences. 1996;21:69-73.

33. El-Khateeb MS, Tarawneh MS, Hijazi S, Kahwaji L. Seroimmunity to rubella virus in Jordanians. Public Health. 1983;97(4):204-7.

34. Hathout H, Al-Nakib W, Lilley H, Abo-Ahmed HS, Nosseir AF. Seroepidemiology of rubella in Kuwait: an alternative vaccination policy. Int J Epidemiol. 1978;7(1):49-53.

35. Bedrossian NK, Matossian R. Is there a rubella problem in Lebanon? Lebanese Medical Journal. 1985;35(1):31-8.

36. Nejmi S. [Immunologic survey of rubella in Moroccan women in the Rabat region (study of antibodies inhibiting hemagglutination in 548 serums)]. Maroc medical. 1972;52(559):420-5.

37. Iqbal A, Bokhari S. Occurrence of rubella antibody IgG in the general population. Mother and Child. 1997;35(1):17-22.

38. Ahmed R, Hashmi K, Ullah SE, Khanum T, Rafia A. Study of Prevalence of Immune Status in Adult Females For Rubella Virus Infection. Pakistan Journal of Biological Sciences. 2006;9(5):816.

39. Hossain A. Seroepidemiology of rubella in Saudi Arabia. Journal of tropical pediatrics. 1989;35(4):169-70.

40. Saeed A, Abu-Shagra S, Al-Rasheed R. Congenital Rubella Syndrome - revisited (letter). Saudi Med J. 1993;14(25-26).

41. Nabli B. [Seroepidemiology of rubella in Tunisia]. Bull World Health Organ. 1970;42(6):891-6.

42. Strauss J, Dobahi SS, Danes L, Kopecky K, Svandova E. Serological survey of rubella in Yemen in 1985. Journal of hygiene, epidemiology, microbiology, and immunology. 1989;33(2):163-7.

43. Sallam TA, Al-Jaufy AY, Al-Shaibany KS, Ghauth AB, Best JM. Prevalence of antibodies to measles and rubella in Sana'a, Yemen. Vaccine. 2006;24(37-39):6304-8.

44. Glikmann G, Petersen I, Mordhorst CH. Prevalence of IgG-antibodies to mumps and measles virus in non-vaccinated children. Dan Med Bull. 1988;35(2):185-7.

45. Edmunds WJ, Gay NJ, Kretzschmar M, Pebody RG, Wachmann H. The pre-vaccination epidemiology of measles, mumps and rubella in Europe: implications for modelling studies. Epidemiol Infect. 2000;125(3):635-50.

46. Morgan-Capner P, Wright J, Miller CL, Miller E. Surveillance of antibody to measles, mumps, and rubella by age. BMJ. 1988;297(6651):770-2.

47. Ukkonen P. Rubella immunity and morbidity: impact of different vaccination programs in Finland 1979-1992. Scand J Infect Dis. 1996;28(1):31-5.

48. Malakmadze N, Zimmerman LA, Uzicanin A, Shteinke L, Caceres VM, Kasymbekova K, et al. Development of a rubella vaccination strategy: contribution of a rubella susceptibility study of women of childbearing age in Kyrgyzstan, 2001. Clinical infectious diseases : an official publication of the Infectious Diseases Society of America. 2004;38(12):1780-3.

49. Dumitrescu R, Mateescu M, Gaicu N, Comanescu D. Evaluation of the anti-rubella immunity levels on a lot of 5,000 sera from women at procreative age, tested by HAI, in Romania. Archives roumaines de pathologie experimentales et de microbiologie. 1989;48(3):253-63.

50. Aksakal FN, Maral I, Cirak MY, Aygun R. Rubella seroprevalence among women of childbearing age residing in a rural region: is there a need for rubella vaccination in Turkey? Jpn J Infect Dis. 2007;60(4):157-60.

51. Pehlivan E, Karaoglu L, Ozen M, Gunes G, Tekerekoglu MS, Genc MF, et al. Rubella seroprevalence in an unvaccinated pregnant population in Malatya, Turkey. Public Health. 2007;121(6):462-8.

52. Sasmaz T, Kurt AO, Ozturk C, Bugdayci R, Oner S. Rubella seroprevalence in women in the reproductive period, Mersin, Turkey. Vaccine. 2007;25(5):912-7.

53. Nessa A, Islam MN, Tabassum S, Munshi SU, Ahmed M, Karim R. Seroprevalence of rubella among urban and rural Bangladeshi women emphasises the need for rubella vaccination of pre-pubertal girls. Indian journal of medical microbiology. 2008;26(1):94-5.

54. Seth P, Manjunath N, Balaya S. Rubella infection: the Indian scene. Rev Infect Dis. 1985;7 Suppl 1:S64-7.

55. Chakravarty MS, Gupta B, Das BC, Mukherjee MK, Mitra AC, Sarkar JK. Seroepidemiological study of rubella in Calcutta. The Indian journal of medical research. 1976;64(1):87-92.

56. Khare S, Banerjee K, Padubidri V, Rai A, Kumari S, Kumari S. Lowered immunity status of rubella virus infection in pregnant women. The Journal of communicable diseases. 1987;19(4):391-5.

57. Khare S, Gupta HL, Banerjee K, Kumari S, Kumari S, Gupta HL. Seroimmunity to rubella virus infection in young adult females in Delhi. The Journal of communicable diseases. 1990;22(4):279-80.

58. Upreti SR, Thapa K, Pradhan YV, Shakya G, Sapkota YD, Anand A, et al. Developing rubella vaccination policy in Nepal--results from rubella surveillance and seroprevalence and congenital rubella syndrome studies. J Infect Dis. 2011;204 Suppl 1:S433-8.

59. Desudchit P, Chatiyanonda K, Bhamornsathit S. Rubella antibody among Thai women of childbearing age. Southeast Asian J Trop Med Public Health. 1978;9(3):312-6.

60. Macnamara FN, Mitchell R, Miles JA. A study of immunity to rubella in villages in the Fiji islands using the haemagglutination inhibition test. J Hyg (Lond). 1973;71(4):825-31.

61. Lam SK. The seroepidemiology of rubella in Kuala Lumpur, West Malaysia. Bull World Health Organ. 1972;47(1):127-9.

62. Doraisingham S, Goh KT. The rubella immunity of women of child-bearing age in Singapore. Annals of the Academy of Medicine, Singapore. 1981;10(2):238-41.

63. Black FL. Measles active and passive immunity in a worldwide perspective. Prog Med Virol. 1989;36:1-33.

64. Yuan CF, Ng HT. Seroepidemiologic study of rubella in Taiwan's female population. American journal of public health. 1988;78(10):1366-7.

65. Miyakawa M, Yoshino H, Yoshida LM, Vynnycky E, Motomura H, Tho le H, et al. Seroprevalence of rubella in the cord blood of pregnant women and congenital rubella incidence in Nha Trang, Vietnam. Vaccine. 2014;32(10):1192-8.

66. Aksit S, Timocin A, Turpculu A. Rubella immunity in pregnant Turkish women. International journal of gynaecology and obstetrics: the official organ of the International Federation of Gynaecology and Obstetrics. 1999;66(1):33-4.

67. Wannian S. Rubella in the People's Republic of China. Reviews of Infectious Diseases. 1985;7(Supp 1):S72.

68. Schenzle D. An age-structured model of pre- and post-vaccination measles transmission. IMA J Math Appl Med Biol. 1984;1(2):169-91.

69. UN Statistics Division UNPD. World Population Prospects. 2012.

70. Abbey H. An examination of the Reed-Frost theory of epidemics. Hum Biol. 1952;24(3):201-33.

71. Mossong J, Hens N, Jit M, Beutels P, Auranen K, Mikolajczyk R, et al. Social contacts and mixing patterns relevant to the spread of infectious diseases. PLoS Med. 2008;5(3):e74.

72. Horby P, Pham QT, Hens N, Nguyen TT, Le QM, Dang DT, et al. Social contact patterns in Vietnam and implications for the control of infectious diseases. PLoS One. 2011;6(2):e16965.

73. Anderson RM, May RM. Infectious diseases of humans. Dynamics and control. Oxford: Oxford University Press; 1991.

74. Turnbull FM, Burgess MA, McIntyre PB, Lambert SB, Gilbert GL, Gidding HF, et al. The Australian Measles Control Campaign, 1998. Bull World Health Organ. 2001;79(9):882-8.

75. Heywood AE, Gidding HF, Riddell MA, McIntyre PB, MacIntyre CR, Kelly HA. Elimination of endemic measles transmission in Australia. Bull World Health Organ. 2009;87(1):64-71.

76. Gidding HF. The impact of Australia's measles control programme over the past decade. Epidemiol Infect. 2005;133(1):99-105.

77. Kearns MJ, Plitt SS, Lee BE, Robinson JL. Rubella immunity among pregnant women in a Canadian provincial screening program. Can J Infect Dis Med Microbiol. 2009;20(3):73-7.

78. Furesz J. Elimination of measles in the Americas. CMAJ. 1996;155(10):1423-6.

79. Haargaard B, Wohlfahrt J, Fledelius HC, Rosenberg T, Melbye M. A nationwide Danish study of 1027 cases of congenital/infantile cataracts: etiological and clinical classifications. Ophthalmology. 2004;111(12):2292-8.

80. Glismann S. Rubella in Denmark. Euro Surveill. 2004;9(4):12-3.

81. Ueda K. Development of the rubella vaccine and vaccination strategy in Japan. Vaccine. 2009;27(24):3232-3.

82. Narimah A. Rubella immunization in Malaysia--20 years on, and the challenges ahead. Med J Malaysia. 2005;60(3):267-8.

83. Ministry of Health. Immunisation handbook. Wellington: 2006.

84. Reid S. Evolution of the New Zealand childhood immunisation schedule from 1980: a personal view. N Z Med J. 2006;119(1236):U2035.

85. Palihawadana P, Wickremasinghe AR, Perera J. Seroprevalence of rubella antibodies among pregnant females in Sri Lanka. Southeast Asian J Trop Med Public Health. 2003;34(2):398-404.

86. Bottiger M, Forsgren M. Twenty years' experience of rubella vaccination in Sweden: 10 years of selective vaccination (of 12-year-old girls and of women postpartum) and 13 years of a general two-dose vaccination. Vaccine. 1997;15(14):1538-44.

87. Preblud SR, Serdula MK, Frank JA, Jr., Brandling-Bennett AD, Hinman AR. Rubella vaccination in the United States: a ten-year review. Epidemiol Rev. 1980;2:171-94.

88. Progress toward elimination of rubella and congenital rubella syndrome--the Americas, 2003-2008. MMWR Morb Mortal Wkly Rep. 2008;57(43):1176-9.

89. CAREC. Rubella vaccination. In: CAREC, editor. 1995.

90. CAREC. Rubella mass campaigns. 2001.

91. Anis E, Grotto I, Moerman L, Kaliner E, Warshavsky B, Slater PE, et al. Rubella in Israel after the MMR vaccine: elimination or containment? J Public Health Policy. 2013;34(2):288-301.

92. Levine H, Ankol OE, Rozhavski V, Davidovitch N, Aboudy Y, Zarka S, et al. Rubella seroprevalence in the first birth cohort reaching fertility age after 20 years of two dose universal vaccination policy in Israel. Vaccine. 2012;30(50):7260-4.

93. Tharmaphornpilas P, Yoocharean P, Rasdjarmrearnsook AO, Theamboonlers A, Poovorawan Y. Seroprevalence of antibodies to measles, mumps, and rubella among Thai population: evaluation of measles/MMR immunization programme. J Health Popul Nutr. 2009;27(1):80-6.

94. Nardone A, Tischer A, Andrews N, Backhouse J, Theeten H, Gatcheva N, et al. Comparison of rubella seroepidemiology in 17 countries: progress towards international disease control targets. Bull World Health Organ. 2008;86(2):118-25.

95. Robertson SE, Cutts FT, Samuel R, Diaz-Ortega JL. Control of rubella and congenital rubella syndrome (CRS) in developing countries, Part 2: Vaccination against rubella. Bull World Health Organ. 1997;75(1):69-80.

96. Robertson SE, Featherstone DA, Gacic-Dobo M, Hersh BS. Rubella and Congenital Rubella Syndrome: global update. Rev Panam Salud Publica. 2003;14(5):306-15.

97. Ouattara SA, Brettes JP, Aron Y, Akran V, Meite M, Sanogo I, et al. [Rubella in pregnant women in Abidjan (Ivory Coast)]. Bulletin de la Societe de pathologie exotique et de ses filiales. 1987;80(2):149-54.

98. Develoux M, Pecarrere JL, Ahounou R, Tinni A. [Anti-rubella antibodies in pregnant women in Niamey (Niger)]. Bulletin de la Societe de pathologie exotique (1990). 1991;84(5 Pt 5):509-12.

99. Cutts FT, Vynnycky E. Modelling the incidence of Congenital Rubella Syndrome in developing countries. Int J Epidemiol. 1999;28(6):1176-84.
